# Author Correction: Proof of concept for a new sensor to monitor marine litter from space

**DOI:** 10.1038/s41467-024-51739-2

**Published:** 2024-08-21

**Authors:** Andrés Cózar, Manuel Arias, Giuseppe Suaria, Josué Viejo, Stefano Aliani, Aristeidis Koutroulis, James Delaney, Guillaume Bonnery, Diego Macías, Robin de Vries, Romain Sumerot, Carmen Morales-Caselles, Antonio Turiel, Daniel González-Fernández, Paolo Corradi

**Affiliations:** 1 Departamento de Biología, Facultad de Ciencias del Mar y Ambientales, Universidad de Cádiz and European University of the Seas (SEA-EU), Puerto Real, Spain; 2 Institute of Marine Sciences (ICM-CSIC), Barcelona Expert Center, Barcelona, Spain; 3 ARGANS France, Sophia-Antipolis, cedex, France; 4 Universitat Politècnica de Catalunya (UPC), Barcelona, Spain; 5 Istituto di Scienze Marine - Consiglio Nazionale delle Ricerche (ISMAR-CNR), Lerici, La Spezia Italy; 6 Technical University of Crete, School of Chemical and Environmental Engineering, Chania, Greece; 7 ARGANS Ltd., Plymouth, United Kingdom; 8 Airbus Defence and Space, Toulouse, France; 9 European Commission, Joint Research Centre, Ispra, Italy; 10 The Ocean Cleanup, JH Rotterdam, The Netherlands; 11 ACRI-ST, Sophia-Antipolis, France; 12 European Space Agency - ESTEC, Noordwijk, The Netherlands

**Keywords:** Marine chemistry, Environmental impact, Physical oceanography

Correction to: *Nature Communications* 10.1038/s41467-024-48674-7, published online 14 June 2024

The original version of this Article contained an error in Fig. 5, in which the colour coded precipitation range legend contained wrong values. This has been corrected in both the PDF and HTML versions of the Article.

