# Supplementary material for: Proof of concept for a new sensor to monitor marine litter from space
Source: Nat Commun. 2024 Jun 14;15:4637. doi: 10.1038/s41467-024-48674-7 (PMC11178853; doi:10.1038/s41467-024-48674-7)
Supplement: Supplementary file 1 — Supplementary Information [file 41467_2024_48674_MOESM1_ESM.pdf]

Proof of concept for a new sensor to monitor marine litter from space

- Supplementary Information -

Authors: Andrés Cózar[1,*,†], Manuel Arias[2,3,4*,†], Giuseppe Suaria[5], Josué Viejo[1], Stefano Aliani[5], Aristeidis Koutroulis[6], James Delaney[7], Guillaume Bonnery[8], Diego Macías[9], Robin de Vries[10], Romain Sumerot[11], Carmen-Morales Caselles[1], Antonio Turiel[3], Daniel González-Fernández[1], Paolo Corradi[12].

Affiliations:

[1] Departamento de Biología, Facultad de Ciencias del Mar y Ambientales, Universidad de Cádiz and European University of the Seas (Sea-EU), 11510 Puerto Real, Spain.

[2] Institute of Marine Sciences (ICM-CSIC), Barcelona Expert Center, E-08003, Barcelona, Spain

[3] ARGANS France, 06904 Sophia-Antipolis cedex, France

[4] Universitat Politècnica de Catalunya (UPC), 08034 Barcelona, Spain

[5] Istituto di Scienze Marine - Consiglio Nazionale delle Ricerche (ISMAR-CNR), 19032 Lerici, La Spezia, Italy.

[6] Technical University of Crete, School of Chemical and Environmental Engineering, 73100 Chania, Greece.

[7]ARGANS Ltd., Plymouth PL6 8BX, United Kingdom

[8] Airbus Defence and Space, 31400 Toulouse, France.

[9] European Commission Joint Research Centre, Directorate D – Sustainable Resources, European Commission, Ispra, Italy.

[10] The Ocean Cleanup, Batavierenstraat 15, 3014 JH Rotterdam, The Netherlands.

[11] ACRI-ST, 06904 Sophia-Antipolis, France.

[12] European Space Agency - ESTEC, 2200 AG Noordwijk, The Netherland

[*]Corresponding authors. Email: andres.cozar@uca.es (A.C.); marias@icm.csic.es (M.A.)
[†]These authors contributed equally to this work.

This PDF file includes:

- Supplementary Methods
- Supplementary Discussion
- Supplementary Figs. S1 to S39
- Supplementary Tables S1 to S36
- Supplementary References
- Acronyms

Table of Contents

SUPPLEMENTARY METHODS **3**

OVERVIEW 3

S1. SUPPLEMENTARY METHODS FOR MISSION CONCEPTUALIZATION 4

S1.1. Critical review of EO4ML status 4

S1.2. Experimental plan 4

S1.2.1. Lab experiments 5

S1.2.2. Radiative Transfer Modelling experiments 6

S1.3. Mission Concept and way forward 13

S1.3.1. Specifying a potential EO4ML Mission 13

S1.3.2. Detection principle and radiometric requirements 13

S2. SUPPLEMENTARY METHODS FOR PROOF OF CONCEPT 17

S2.1. Definition of the PoC data processor 17

S2.1.1. Atmospheric correction 17

S2.1.2. Cloud masking for the PoC 19

S2.1.3. Land masking 22

S2.1.4. Detector footprint masking 22

S2.1.5. Filtered data 23

S2.1.6. Additional metadata 23

S2.1.7. Windrows Spectral Index detection 24

S2.1.8. Filament contextual identification 27

S2.2. Testing of the PoC processor 34

S2.2.1. Known issues 34

S2.2.2. Automated pre-screening functionalities 35

S2.2.3. Verification sites 37

S2.2.4. Initial supervision test of the PoC processor 37

S2.2.5. Validation 38

S2.3. Data processing 38

S2.3.1. Data acquisition 38

S2.3.2. PoC processor outputs 38

S2.3.3. Supervision of the PoC processor outputs 39

S2.4. Detection threshold for the PoC processor 40

SUPPLEMENTARY DISCUSSION **41**

S3. SUPPLEMENTARY DISCUSSION FOR MISSION CONCEPTUALIZATION 41

S3.1. Experimental Plan 41

S3.1.1. Lab experiments 41

S3.1.2. Radiative Transfer Modelling experiments 43

S3.1.3. Findings of the experimental plan 49

S3.2. Mission Concept and way forward 50

S3.2.1. Revisited applications and mission requirements 50

S3.2.2. Development Plan 51

S4. SUPPLEMENTARY DISCUSSION FOR PROOF OF CONCEPT 56

SUPPLEMENTARY FIGURES **57**

SUPPLEMENTARY TABLES **87**

SUPPLEMENTARY REFERENCES **116**

ACRONYMS **120**

Supplementary Methods

Overview

The aim of the present study was, on one hand, to define an EO mission concept specific to the sustained global-scale ML monitoring and, on the other hand, to test the feasibility of this concept. Accordingly, the present section is divided into two main parts, one with the methods used for the Mission Conceptualization (MC), and a second part dedicated to describe the methodology used for the Proof of Concept (PoC). The overall roadmap combining these two major objectives (MC and PoC) is presented in Supplementary Figure S1. Its completion spanned a period of over six years under three linked contracts with ESA.

To date, there is no mission concept definition for ML, although there were relevant joint analyses regarding its requirements, in which our working group participated[1,2]. Our purpose with the MC was to define main features and establish minimal base requirements for a potential EO mission founded on a dedicated spectral analyser instrument to monitor ML on the ocean surface (EO4ML). To do so, we carried out an analysis of the spectral and other physical ML properties potentially useful for space-based sensors, derive technical requirements, and contrast them against existing and near-future technologies, and plausible scenarios for ML observation at sea. The data for this exercise were generated in the experimental phase, consisting of laboratory, field, and modelling tests. Part of the results of this experimental phase, related to the spectral profiling of ML, was already reported by the working group in previous papers, both laboratory analyses[3,4] and at sea experiments[5,6]. Spectral information was used to cope with the needs of the modelling exercise, and so to determine theoretical specifications for the key aspects of the mission, particularly on the identification of candidate spectral bands and signal-to-noise ratios. Finally, the trade-off analysis on technical requirements and detection performance under real observation conditions resulted in the definition of an optimal EO4ML mission.

The PoC assessed the theoretical requirements against the specifications of existing sensors. A partial match was found with in-orbit Multi-Spectral Instrument of the EU Copernicus Sentinel-2 mission (S2-MSI), which opened an opportunity to assess capabilities of a potential EO4ML mission. This exercise required the development of a specific PoC data processor able to detect ML in S2-MSI multispectral images. Previous studies point to the so-called litter windrows (LW) as the most feasible target for detection from space[7-9]. However, knowledge of these sub-mesoscale convergence structures of floating material is very limited. LWs are generally overlooked due to their dispersion, small size and ephemeral nature. In this context, the working group launched a call for field research on LWs[10], which resulted in a series of contributions made in collaboration with other groups[11-14]. On this basis, the PoC carried out a massive processing of the time series of S2-MSI images available for the Mediterranean Sea in a High-Performance Computing (HPC) facility, in order to obtain broad-scale maps and trends to assess their consistency and usefulness for scientific and management purposes.

S1. Supplementary Methods for Mission Conceptualization

Marine litter (ML) is any human-made item that has ended up in the marine environment. The MC addressed plastic polymers as core targets. Plastic, as a synthetic material, has the advantage of being unequivocally linked to human activity, as well as being particularly persistent and abundant among ML[15]. Other abundant floating materials, natural or not, such as algae, driftwood or seafoam were considered as sources of interference for plastic detection, although additional spectral bands were also defined to specifically address the quantification of these materials in a more ambitious EO4ML mission. The process of Mission Conceptualization (MC) was composed of the following steps:

- a critical review of the state-of-the-art on scenarios and opportunities for EO4ML;
- an experimental set up to determine the detection principles and optimal scenarios;
- a technological assessment to identify the instrument and technical requirements.

Some contributions to these aspects were already reported in papers we have authored or co-authored. When pertinent, we will refer to such publications so readers can find full methodologies and extended results, avoiding duplication of previously published work.

S1.1. Critical review of EO4ML status

Our findings in this section were shared with those of other groups and reported in two joint papers[1,2]. Our analysis covered the following issues:

Understanding ML from the perspective of remote sensing: The analysis of existing knowledge was key to determine ML properties best suited to be explored from a remote sensing point of view. The analyses comprised composition, concentration ranges, and spatial and temporal variability, in order to get insight on spatio-temporal scales and patterns relevant to the ML problem, as well as physical characteristics to be potentially targeted[1].

Identification of ML scenarios of interests for the community: Any proposal for an EO mission must be tailored to the needs of the community of users and experts. To that end, we surveyed representatives of user communities and ML experts to identify the use cases of most relevance to them, including aspects for the definition of a technical solution such as time sampling, spatial resolution, and levels of ML characterisation required in each case[1,2]. Five scenarios were identified as being of most interest to the community.

Elaboration of preliminary Mission Requirements (MRs)*:* To define the technologies best fit for the five scenarios, user requirements and the knowledge of ML characteristics were translated into MRs. This was done by assessing needs in terms of GSD, revisiting time, spatial coverage, AOIs and objectives. Those relate to the required spatial resolution, measurement frequency, spatial scales of relevance, key target areas of observation, and specific aims (detection, quantification, monitoring, identification, characterization)[1,2]. Table S1 summarises the results.

Selection of available candidate techniques and technologies: Once the MRs were established for each scenario, they were contrasted against a set of existing sensing technologies, instruments and missions (Tables S2 to S6, Fig. S2). The instruments and sensors used in the exercise were limited to those having a more promising compatibility with the scenarios and observable ML properties[1,2]. Our critical technical analysis led to an optical multispectral mission as the most promising combination of technologies and capabilities that could comply with the scenarios identified by the community[1] (Table S7).

Assessment of the most suitable candidate scenario and mission: Based on the outcomes of the technological assessment, we evaluated the compatibility of the candidate technologies with the five observational scenarios (Table S8). We determined the best scenarios for an ML-dedicated mission concept to (i) detection and quantification of concentrations of ML at a global scale, and (ii) detection and monitoring of hotspots and accumulation zones. Our MC focused on these two scenarios, and the aim of the experimental plan was to discern which of the two was more feasible, or even if both could be achieved from a single mission concept.

S1.2. Experimental plan

The experimental plan aimed to empirically support the definition of the EO4ML mission concept, based on the best spectral techniques that could be devised for the mission. It consisted of two sequential parts:

- field measurements of plastic polymers, to obtain a reference spectral library and better understand the distinguishable optical properties of plastics that could be exploited by an EO4ML mission.
- a Radiative Transfer Modelling (RTM) to determine the detectability limits for a hypothetical instrument in orbit, the target spectral bands, bandwidths, as well as the optimal observing geometry.

The following sections detail how both experiments were carried out. Note that, for the case of the lab experiments, we reported a substantial part of methods and results in a previous paper[3], so the contents here indicated will be succinct.

S1.2.1. Lab experiments

The so-called Great Pacific Garbage Patch (GPGP) is considered the largest plastic accumulation zone in the open ocean[16]. From July 27th to September 19th 2015, the Ocean Cleanup conducted extensive sampling in the GPGP, aimed at better quantifying and characterizing ocean plastics[17]. Taking advantage of the large amount of plastic material collected on this expedition, we conducted a series of controlled laboratory experiments for the spectral characterisation of both raw polymers and plastic samples harvested in the GPGP.

Out of the floating marine debris collected with their surface trawls, 99% was made of plastic (both in number and weight). Fourier Transform Infrared Spectroscopy (FT-IR) analyses conducted on the marine harvested litter showed a major contribution of debris made of polyethylene (72.9% of fragments, 64.3% of lines) followed by polypropylene (27.1% of fragments and 34.3% of lines and filaments). In regard to plastic size, Lebreton and co-workers[17] estimated that the inner GPGP region contains around 6,000 tonnes of microplastics (0.5-5 mm; 680,000 pieces per km^{-2}), 10,000 tonnes of mesoplastics (0.5-5 cm; 20,000 pieces km^{-2}), 20,000 tonnes of macroplastics (5-50 cm; 690 pieces km^{-2}) and 40,000 tonnes of mega plastics (> 0.5 m; 4 pieces km^{-2}). In terms of plastic types, it was estimated that the inner GPGP has around 35,000 tonnes of hard/rigid plastics (665,000 pieces km^{-2}), 40,000 tonnes of ropes and fishing nets (21,000 pieces km^{-2}), 337 tonnes of pellets (14,000 pieces km^{-2}), and 35 tonnes of foamed plastics (228 pieces km^{-2}). Even though this experiment was focused on a single accumulation zone, it is to be expected that the plastic composition described here will also be applicable to other open-ocean accumulation zones. Previous characterizations of these regions also converge on the prevalence of PE and PP hard plastic objects, derelict ropes and fishing gear[15,16,18].

The Ocean Cleanup also estimated the percentage of sea surface covered by plastic debris for 7 different size intervals (0.05-0.15, 0.15-0.5, 0.5-1.5, 1.5-5, 5-10, 10-50 and >50 cm), assuming that the area covered by individual items in these intervals to be those of square with side length (*i.e.*, 0.1, 0.325, 1, 3.25, 7.5, 30, 100 cm). The average percentage of sea surface covered by plastic ranged from 0.001 to 0.01% inside the accumulation zone, with a major portion of the plastic coverage being represented by macroplastic objects (10-50 cm)[17].

These figures show that, even over highly polluted areas, the ocean surface physically covered by plastic litter is considerably low, in close agreement with other studies[19,20]. In our experiments, we analyse sea surface coverages ranging from 100% to 0.01% (Fig. S3). EO technologies shall have a special focus on the capability to detect a weak signal out of the background signal. However, at the date of this work, proper sensitivity studies using marine-harvested plastic were not carried out.

Objectives of the lab experiment

The main objective of the lab experiments was to obtain high-resolution spectral reflectance measurements from different concentrations and compositions of marine-harvested plastics in sea water using a Malvern Panalytical - Analytical Spectral Device (ASD) FieldSpec 4 spectroradiometer from the UV (350 nm) to SWIR (2500 nm) spectrum. Those were not available in publications to the scientific community at the time. Thus, their acquisition was of relevance for the study and in order to assess the potential remote sensing requirements based on 'real' ML collected by the Ocean Cleanup in the GPGPG. From the spectral information gathered in this experiment, we aimed:

1. To identify unique and optimal wavebands for oceanic plastic remote sensing and estimate the minimal signal-to-noise ratio (SNR) for relevant optical sensors.
2. To better assess compatibility with existing airborne[21,22] and satellite optical sensors to transpose airborne measurements into technical specifications for spaceborne systems.
3. To determine the minimum threshold of debris concentration for potential observation in spectral bands using the following composition:
    a. Raw new plastic, five different series of tests for HDPE, LDPE, PP, PS and a mix of PE-PP representative of plastic composition in the GPGP.

b. Marine-harvested plastic, from the GPGP, mostly made of PE and PP, four different series of tests for: hard fragment, fishing-related plastics (nets, ropes, threads), pre-production pellets and foamed plastics (mainly styrofoam).
c. Marine-harvested plastics with bio-fouled organisms, collected from the GPGP and currently frozen.
4. For the proposed different scenarios of plastic composition, to build a spectral reflectance library for variable concentrations ranging from 0 to 100% of sensor field of view or surface pixel covered and for variable sensor geometry from 0° to 45° nadir angles (to take into account potential Apparent Optical Properties, AOPs), using ambient light or solar lamps depending on weather conditions.
5. Based on the above, to identify the spectral variations of the plastic marine debris as result of aging effects for each type of polymer analysed in this study.

The temporal revisit interval and spectral and geo-spatial resolution for identification, tracking and quantification of marine floating plastics were explored by coupling laboratory and airborne spectral measurements to an RTM. We tested different configurations for ocean plastic concentrations, type of plastic debris and observation geometry. The results allowed for the prototyping of potential detection algorithms that could be made part of the EO4ML mission Concept provided within the project.

Equipment and Experimental Setup

Readers are invited to access the paper by Garaba *et al.* for details on the equipment used, the experimental setup and methods employed in this part of the experimental plan[3].

S1.2.2. Radiative Transfer Modelling experiments

Following the experimental phase, a radiative transfer modelling exercise was carried out in order to optimize the trade-off between technical requirements and top-of-atmosphere (TOA) sensing performance over a wider range of observing conditions. Simulation parameters were set to try to represent the observational conditions of litter over the sea surface in open ocean conditions. This was selected following the results shown in previous sections on ML characterization and feasibility from existing and future instruments.

Model methodology

The simulation experiment was based on the existing knowledge about the spectral behaviour of ML, which is rather limited. Our lab experiments allowed us to answer some of the key questions, but many remain unanswered and probably additional efforts will be required to solve them. Overall, the modelling exercise was formed of three components:

- Generation of a water-leaving reflectance at BOA.
- Radiative transfer problem from BOA to TOA.
- Identification of SNR.

Generation of a water-leaving reflectance at BOA

In order to simulate the spectral signature at TOA, it was necessary to know it in first place at BOA, or at z = 0 km. Defining the optical properties of the water-leaving radiance L_w^0 is not an easy task. To do this, the method described by Mobley[23] is outstanding to date.

L_w^z is the result of the directly reflected and the scattered emissivity with optical paths ending in the sensor situated at an altitude z. At the BOA level, the contributions consist of the reflection and scattering taking place at the very surface, plus all the upwards radiance as a consequence of the reflection and scattering within the water column. Water composition, depth and particle contents determine the spectra of the light leaving the water column and adding to the total observed reflectance. Other atmospheric contributions are not considered at this point, but they play a role in both the down-welling emissivity E_d and up-welling emissivity E_u.

Bi-directional Reflectance Distribution Functions (BRDF)

Regardless the light source, L_w^0 is frequency-dependent, and different behavior could be expected according to each considered wavelength. Indeed, for a given photon of any wavelength, there is a probability of being reflected, scattered or absorbed, depending on what medium it travels through. Under Lambertian conditions, these parameters are independent of the geometry of the observation, and probabilities are the same ones for all photons, despite the direction they come from. Eventually, it is the net effect that matters, and these various optical phenomena can be expressed as a value of reflectance or albedo.

Whenever this situation differs and there is a geometrical dependency, the problem becomes more complicated and parameters vary according to the incident angle of the photons, the direction of the surface with respect to the incoming light, and the position of the sensors. Solving this multiparametric model results in the so-called bidirectional reflectance distribution functions or BRDFs.

For a perfectly mixed ocean of infinite depth, it is often assumed that it behaves as a Lambertian surface, and for many applications this is a fair assumption. However, the assumption usually only holds whenever we have a low wind speed incising over the water's surface and when we are out of the glint area. If these factors are not preserved, then the surface does not behave as Lambertian and BRDFs shall be constructed.

In our case we wished to take into account the effect of the wind, which increases scattering and the radius of influence of the glint. We also aimed to assess the impact of glint, so again, there was an angular dependency that had to be considered.

The modelling becomes quite more complex if we consider the optical properties the particles in water could have. For instance, due to their shape, size and composition, those particles can have a noticeable effect on the values of L_w^0. These particles' inherent optical properties, or IOPs, can be identified with laboratory measurements via standard procedures used for any ocean color application.

Most common procedure to determine these IOPs consists in putting the particles in suspension within the medium of interest and analysing the behaviour of artificial light traveling through that medium. The objective of those measurements is to obtain a statistically meaningful behaviour associated with the integrated effect of the particles in suspension. Photons are bounced and scattered many times, thus increasing the chance for further absorption and geometrical dependencies.

In our case, we discarded measuring these IOPs due to two main reasons. First, we expected that most of the interesting or specific signatures for the polymers will be within the NIR/SWIR regions of the spectrum, while water absorption in these ranges is considerable, so particles suspended in water are very unlikely to generate a measurable impact. Secondly, IOPs are only meaningful under the assumption of a well-mixed medium, in which there is not a preferential directionality in the particle distribution. However, this is not the case for plastic polymers staying at the water column, and within the photic area of the water column, they tend to accumulate at the surface or very near to it[24,25].

Thus, the best option lies in the consideration of the bulk effect of a layer of plastic particles set in the surface or near to it. We tried to identify any potential AOPs that could show any particular angular dependency, during the field experiments of this document. Unfortunately, no specific angular dependency patterns were found, mainly due to some experimental limitations and the complexity of such measurements[3].

As a consequence, for the generation of the BRDFs for the different scenarios, we discarded any specific angular dependency for the plastic component of the sea water. We maintained, however, the angular dependencies that are known for the sea water fraction.

Seawater leaving radiance (L_w^0)

For the water contribution to L_w^0 , we used the classic parametrization from Cox and Munk[26], following the approach proposed by Nakajima and Tanaka[27]. This approach estimates L_w^0 using as inputs values of salinity, *Chl-a* and wind speed and direction. These values were used to estimate the reflective properties of the surface and ocean colour. Those are, of course, wavelength dependent, and results are also dependent on the geometry of the observation. An example using a variant of DISORT radiative transfer solver is provided by Jin *et al.*[28].

The choice for this parametrization is that allows for a fast estimation of the BRDF associated with Case I waters. Case I waters are typical of the open ocean, away from coasts and river mouths. *Chl-a* concentration in water ranges

from very low values (0.02 mg m^{-3}) in the oligotrophic subtropical gyres, up to high values, 5-20 mg m^{-3}, in coastal up-welling areas. Case I waters are those in which phytoplankton and associated materials (*e.g.*, detritus heterotrophic organisms and bacteria, excreted organic matter) control the optical properties. Case I waters are relatively simpler to model because only plankton influence optical properties and scattering and absorption by debris would correlate with *Chl-a* concentrations.

On the contrary, tackling Case II waters is more complex. They are found in coastal areas influenced by land drainage or suspended sediment, in addition to phytoplankton. Optical properties are typically controlled by three independent components, namely phytoplankton and their associated debris; dissolved organic matter of terrigenous origin (yellow substance, Gelbstoff, Gilvin or CDOM); and mineral particles and suspended sediments.

Case II water simulations require a dedicated effort and embrace far more possible different situations, as otherwise results would be rather biased towards a specific parametrization of these waters. Nonetheless, it has been already indicated that those types of waters correspond to scenarios where detection of litter is per se more difficult, precisely due to the presence of sediments in water, waves breaking, atmospheric aerosols, etc. Seafloor reflectance also would add to the problem whenever we find shallow waters, when determining the values of L_w^0 .

This optical classification of water, of course, is rather arbitrary, and usually it is hard to do a proper separation between both cases. Indeed, part of the ocean colour community advocates for deprecating it[29]. Nonetheless, around 60% of the total ocean's surface is considered as Case I waters[30], which could be further extended if we just consider also cases where *Chl-a* could be high but CDOM and sediments concentrations are low[30]. The geographical distribution of Case I waters includes all the oligotrophic regions like the subtropical gyres where the great plastic accumulation zones take place[16,18].

All in all, Case I waters are a very representative configuration for the radiative transfer simulation. It also allows for benchmarking a variety of observational situations plus identifying the potential performance that could be obtained for these waters. Additional assessment of more complex water compositions through modelling should require further effort.

Polymer-induced water leaving reflectance

As previously reported, the limitations of the performed measurements did not allow us to conclude that ML optical properties could have specific angular dependencies, at least for the floating ML fraction. Without conclusive information, we assumed litter behaves as a Lambertian surface.

This means that the reflection probability associated with plastic polymers will be equal to the measured reflectance under controlled conditions. Thus, during the computation of L_w^0, we assume that any photon interacting with plastic polymers will have this behavior.

Combination of contributions

From the previous paragraphs, the simulation exercise requires a methodology to combine contributions of both sea water and plastic polymers to L_w^0. This combination is related to the total fraction of plastic polymers included in a given pixel.

We assumed all the floating plastic litter is found at surface level, covering a varying percentage of the pixel surface. Down-welling photons have a chance to interact with the plastic, equal to the fraction of the surface covered. If this event does not take place, the photon travels normally to the sea water and follows the normal computation of scattering/absorption probabilities (Fig. S4).

A fraction of the incident light over the sea surface is reflected directly, and other penetrates water and is eventually reflected backwards, becoming water-leaving up-welling emissivity, E_u. These up-welling photons have another chance to interact with the plastic particles in the surface, being absorbed, scattered or reflected back to the water column, further increasing the chances for not leaving the sea water at all.

Unfortunately, we did not find specific studies designed to measure the reflective properties of plastic polymers under water, but there are reports of this factor playing an important role. Voss and Zhang showed that Spectralon® (which is made of a plastic fluoropolymer) has a higher reflectance value under water when viewing angles are below 50°[31]. This means a significant change of optical behaviour for a material that is highly Lambertian by all the standards, and that is commonly used as white reference for the calibration of spectrometers, as it was the case during our experiment.

Goddijn-Murphy and Dufaur also brought attention to the changes in the optical properties that could take place in the interface plastic-water, but their study did not go any further apart from some theoretical considerations[32].

It is to be expected that a change of optical density in the medium allows for sharper transitions between the interface plastic-air than in plastic-water. This was pointed out by Berger *et al.*[33], as they performed an interesting study about the BRDFs of different materials under emersed and immersed conditions, which essentially confirms previous observations[31]: the variations in the refractive indices associated with the change in the optical density of the medium are driving different results over the Fresnel equations driving the reflectance. This aspect of the radiative transfer modelling would require a dedicated effort, not addressed here. A good approach to follow is the methodology introduced by Berger *et al.*[33].

We opted to assume that the water side of the plastic particles behaves equally to the aerial side. The assumption likely diminishes the contribution of seawater to the total L_w^0 generated by the simulation, since the probability of an up-welling photon being absorbed by the polymer increases significantly with respect to reality.

The resulting BRDFs were generated out of the probabilistic combination of water-leaving radiance coming from seawater, using the explained Cox & Munk method[26], and up-welling radiance E_u as a result of the interaction with the plastic debris on the surface. Thus, roughness of the surface is considered as parameterized from the input wind speed, but this is not relevant for the plastic fraction, as we considered it as Lambertian in the exercise (*i.e.*, the specific slopes do not change the reflectance values). The proportion of contributions depends on the fraction of plastic coverage provided, and there are some non-linearities in the combination, as a consequence of the underwater reflection of photons when they reach plastic debris in their path towards the surface.

The challenge of radiative transfer

One particular aspect to consider in the simulation was the atmospheric contribution, both coming from gas scattering and absorption, as well as the influence of aerosols. These components could limit the detectability of ML. In general terms, we could expect an increase of the observed L_w^z in the blue region of the spectrum, as per photon scattering and selective absorption, and a significant reduction in some of the NIR/SWIR bands due to the water vapor and its absorption bands. In contrast, there are regions of the electromagnetic spectrum in which the atmosphere has a rather small effect in comparison even to visible bands. Should we find distinct spectral features for plastic polymers in these regions, we could expect better chances for detection. Nonetheless. transmittance is still below 80%, so certain loss could be expected as a result of it.

To address the effect of the atmospheric radiative transfer, we constructed the model considering the following factors:

- the radiative source, *i.e.*, the sun irradiation spectra;
- the gaseous composition of the atmosphere to simulate molecular scattering and absorption;
- the aerosol composition and distribution within the modelled atmosphere.

The variability of these factors is wide, depending mainly on the distance to the sun (or time of the year), latitude and the relative position of the point within the Earth's surface.

The Solar Source

We opted to use the full resolution solar spectrum provided by the Atmospheric & Environmental Research (AER) Radiative Transfer Working Group (Lexington). This source is the most common one being used nowadays for radiative transfer modelling, and it works efficiently for most applications. Whilst the data cover the full range from 200 nm to 20,000 nm, this dataset is precisely optimized for the infrared region, which makes it particularly valuable for our purposes.

Atmospheric composition and profile

To represent the gaseous element of the atmosphere, we applied the Air Force Geophysical Laboratory atmospheric constituent profile U.S. standard 1986 (AFGL-TR-86-0110). Once again, it is one of the most standard profiles for atmospheric composition, which also provides a good vertical grid that we used for outputting at different values of z, from 0 km to 120 km.

To parametrize the molecular absorption and scattering, we selected the REPTRAN model updated in 2014, which allows for a high spectral resolution[34].

Finally, for the aerosols, we used the Optical Properties for Aerosols and Clouds datasets (OPAC library[35,36]). We chose a relatively maritime clean atmosphere, characteristic of the open ocean environment under consideration here. This is equivalent to 50% of relative humidity in the atmosphere, and aerosols distributed within the 0-35 km atmospheric column. The simulations included descriptions for 10 types of aerosols.

Radiative Transfer Solver

The above elements define the physical characteristics of the intervening atmosphere and of the light source. Photons grabbed from the solar source interact with such properties, reaching the bottom of the model or surface under the atmosphere, and being affected by the corresponding BRDF generated. To obtain a physical meaning of these interactions, it is necessary to solve the radiative transfer equation. Taking into account the goals of this study, the 1D approximation was enough.

The photon path was modelled starting from the source, traveling down to the bottom surface, interacting with the surface and traveling back to TOA. There are multiple chances for a photon being scattered, absorbed or reflected during this path. The spectral signature at TOA is the combined effect of the spectral characteristics of the source, the physical properties of the atmosphere and the optical properties of the bottom surface.

To combine all these effects, we opted for a statistical solver called MYSTIC (Monte Carlo code for the physically correct tracing of photons in cloudy atmospheres), developed since 1997[37,38]. This solver emulates the trip performed by individual photons randomly sampled from the light source. The model is probabilistic, and its strongest advantage is that uncertainties can be derived as result of the variability observed in the multiple runs over the same model configuration. Moreover, the solver provides an estimation of the photon noise coming from the multiple occurrences of photons falling within a given frequency range. This approach provides information about the expected natural variability. The drawback is that the model is by far the slowest, as it is computationally intensive. In contrast, heuristic characteristics of the model provide the best match for the interaction with the BRDF generated at surface level. We took advantage of these characteristics later, to find out where signals can overcome the noise.

Identification of the Signal-to-Noise Ratio (SNR)

The main purpose of the identification of the SNR is to learn under what conditions signature of marine plastic litter could be observed. To do this, we were obliged to not only study the raw signal produced by the simulations, but also to assess how different the signal is from background noise. A Relative Signal-to-Noise Ratio (SNR_r) was computed as the comparison of the variability derived from multiple runs of the same scenario run by MYSTIC (combined with the corresponding photon noise), when considering different ML surface coverages and with respect to reference scenarios (free of litter). The aim was to calculate the variability of the signal of ML scenarios being significantly different from the reference scenarios. To do that, we started from a standard SNR definition for spectral measurements:

$$SNR_s(\lambda) = \frac{L_s(\lambda)}{\sigma_s(\lambda)}$$

being s the scenario at hand, L_S the observed radiance at scenario s, and σ_S the observed variability in the same scenario. Typically, to obtain a meaningful result, SNR_s shall be substantially large. In ocean colour, a minimum SNR of 400 is targeted in visible bands[39]. However, besides targeting bands in which the SNR is good enough for a sensor operating correctly, it is also important to identify what bands offer a specific signal related to litter that can allow for its detection against the seawater spectral background. This approach was also identified as a promising method to detect floating matter at sea[21]. It can take the form of regions of the spectrum where litter offers a stronger reflection or regions where litter absorbs more radiation. These features shall appear reflected in the SNR to be detected. One way to do so is through the SNR_r, which can be formulated as follows:

$$SNR_r(\lambda) = \frac{L_s^{litter}(\lambda) - L_s^{water}(\lambda)}{\sigma_s(\lambda)}$$

This expression reflects the SNR that can be found in the differences between a scenario of reference (no litter) and one scenario with a given fraction of litter coverage in the pixel. The most useful wavelengths, or bands, for litter detection are those in which the radiance coming from the floating litter is significantly different from the radiance coming from seawater (assuming plastic is the only floating material). The expression above provides negative values when the marine plastic polymers show more absorption than seawater, and positive in the opposite case.

Values of $|SNR_r(\lambda)| < 1$ means that litter does not generate a signal different enough from the seawater signal to provide a relative signal over the expected uncertainty, which would mean its relative signal cannot be distinguished from noise. Assuming a given number n of simulations, we defined the noise in this exercise (σ_{ref}) as the variability in the results for the given set of simulations. This variability shall be obtained as the RMS of the photon noise for each simulation (σ_{pn}), with the variance of L_w between the different runs (σ_w^2):

$$\sigma_{ref}(\lambda) = \frac{1}{n+1}\sqrt{\left(\sum_{i=1}^{n} \sigma_{pn}^2(\lambda)\right) + \sigma_w^2(\lambda)}$$

with

$$\sigma_w^2(\lambda) = \frac{1}{n}\sum_{i=1}^{n}\left(L_w^i(\lambda) - \underline{L_w}(\lambda)\right)^2$$

The definition of this relative SNR is equivalent to the definition of a χ^2 function as used in statistical inference to determine the maximum likelihood of the fitting of a given estimator. Mathematically, χ^2 compares the variance of the residuals between model and observations with the variance of the observations:

$$\chi^2(f(x)) = \frac{1}{n}\sum_{i=1}^{n}\frac{[f(x_i) - E(x)]^2}{\sigma^2(E(x))}$$

Whilst in inference we hope to have a value of χ^2 close to 1 so that model and observations are not distinguishable, here we search for values clearly distinguishable, *i.e.* substantially larger than 1. In other words, this means that if $|SNR_r(\lambda)|$ is less than or close to 1, the anomaly signal of the water-leaving reflectance cannot be separated from the noise. In practical matters, much larger values than 1 will be required for detectability purposes. This technique is, thus, a powerful criterion to identify spectral bands optimal for floating plastic detection, as well as to isolate the minimal fractions at pixel level that would be necessary to have in order to obtain a measurable signal.

For our experiment, the proposed computation of $SNR_r(\lambda)$ was done directly in terms of radiances, as sensors measure radiance and not reflectance. Nevertheless, the results are still valid, as the reflectance is obtained by normalizing the signal with respect to the light source, which will have no effect on this computation for a given scenario.

Experimental datasets and parametrization

The simulation of the different scenarios requires setting up various parameters along the elements of the radiative transfer model. The choice of these parameters is dictated by the following:

- the modelled scenarios need to be representative of real situations;
- different detectability thresholds need to be identified under multiple observational conditions;
- limitations imposed by computational resources need to be considered.

The first point is relevant to understand the applicability of the results and their validity for the potential uses. It is not only necessary to define the optimal observational parameters, but also to extend the ranges of these realistic values towards some extremes, in order to identify the limits of the results. Finally, it should be considered that the model simulations were heavy in terms of computational times and resources. This factor determined the level of granularity or number of bins addressed for each variable in the model.

Setting up the vertical grid

The number of vertical layers used in the model has no significant effect for the computation in itself, as the full path of the photons has to be solved to obtain a valid RTM simulation at TOA. Nevertheless, a multi-layer approach was selected, using as reference the layers already used in AFGLUS for the atmosphere model. The AFGLUS-based configuration adopts an uneven distribution of vertical layers intended to better resolve the parts of the atmosphere where variability takes place. Therefore, AFGLUS shows the following distribution of values of z:

- From BOA (0 km) to 25 km, bins of 1 km.

- From 25 km to 50 km, bins of 2.5 km.
- From 50 km to TOA (120 km), bins of 5 km.

Note that here TOA is defined at 120 km altitude, on the basis of the minimum altitude in which satellites operate, although satellites are often located at larger distances. However, the preliminary study showed that simulated simulations at different values of z asymptotically converge towards 120 km. Sensitivity of the results tended more rapidly to zero when reaching the highest layers of the atmosphere. This circumstance means that there is little gain by extending further the simulations, as values at high altitude are not significantly different. This holds true for the simulations but not from an instrumental point of view. Resolution of the instrument depends on the distance to the target and its comparative size with respect to the sensor. However, this engineering aspect (*i.e.* the potential effect of a spherical dispersion of the signal with increasing distance to the target) was unaccounted for in these simulations.

Based on the above, BOA was defined as the immediate level at z = 0 km and TOA corresponds to z = 120 km. These terms have this equivalence hereafter in the document.

Setting up the BRDFs

The generation of the BRDFs for both reference and littered scenarios was equivalent, with the only difference being the ML presence. Cox & Munk parametrization[26] relies in some geophysical variables to determine the behaviour of light within water column and surface, namely, chlorophyll concentration (*Chl-a*, in mg/m^3), salinity (S, in parts per thousand or psu), wind speed at z = 10 m (u_{10}, in m/s), and wind vector angle (u_φ, in degrees from South).

Chl-a and S are used mainly to estimate spectral absorption features of seawater and diffraction properties, whilst the wind speed and angle affect the roughness of the surface and how it scatters light. Because of the type of exercise proposed, we did not play with S, u_{10} or u_φ, fixing their values for all the simulations. The effect of changes in S is negligible as well as that of u_φ in a 1D model. In contrast, u_{10} can play a major role in reflectance, mainly in the glint area. Here, we decided to fix u_{10} to explore the effect of its variability in later experiments. Thus, these parameters have been set as follows, *Chl-a* = 0.01, 0.1, 1, and 10 mg/m^3, S = 34.3 psu, u_{10} = 1 m/s, u_φ = 0°. S was chosen as representative of Case I waters from the North Atlantic, matching the latitudinal point used for the geometry. u_{10} was a shy value related to a surface pretty smooth to approach best-case scenarios.

Geometry of observations

The geometry of observation determines the cross-section of the atmosphere that photons travel through, plus the relative angle between light source, surface, and sensor controls phenomena like sun glint.

Sun position and distance drive the intensity of the radiation, plus the maximum height in the sky of the light source which also has an effect in the optical path. These elements were fixed in the simulation scenarios. For the Sun-Earth distance, 1 AU has been chosen (equivalent to the equinox position between Earth and sun). For the point over Earth's surface, a latitude of 45°N and a longitude of 15°W were chosen, corresponding to the North Atlantic.

To test different lighting conditions, the sensor was located at zenith, looking downwards. This position remained fixed in the various scenarios. Regarding the Sun Zenithal Angle (SZA), values ranging from 0° to 75° and at intervals of 15º were tested, being 0° the zenithal position.

According to previous sections, not all plastic polymers are equally frequent in the ML. Particularly in surface waters, most floating litter is made of PE and PP, with PS being the third most abundant. These three polymers, accounting for 85% of the total plastic litter, were the focus of all our simulations.

A range of concentrations in the logarithm scale was taken for the different scenarios. Selected fractions of surface coverage (*f*) were of 0, 0.001, 0.01, 0.1, and 1 (0%, 0.1%, 1%, 10% and 100% in percent, respectively), with 0 being the reference scenario (no litter is present). This scale was used as a first order approximation to determine the order of magnitude of littering that should be present in a single pixel to achieve detection.

Note that obtaining a quantitative disentangling algorithm could likely be through a spectral inversion model and in an end-to-end simulator, activity falling beyond the scope of this work.

A full spectral range from 350 nm to 2450 nm was analysed to match the range used in the field experiments for the in-situ measurements of polymers carried out with the ASD. Whilst unique spectral features for plastic polymers are mainly found in NIR and SWIR regions, characteristics of plastics in the visible bands could be also potentially useful.

Indeed, a major fraction of the floating plastic debris is of white color[40], which could be used to discriminate plastic polymers from other concurring floating substances or materials (*e.g.*, *Sargassum*, foam, driftwood)[41].

To help define the optimal central wavelengths and bandwidths, the simulation was performed line-by-line and using steps of 1 nm. This choice allowed for a virtually full spectral characterization; however, it made the simulation more time-consuming and results much noisier. In this regard, smoothing filters could be later applied to the results.

Final considerations

Each of the 3,600 potential scenarios was simulated 10 times, including reference and littered ones. Each of those scenarios involved simulating 2,100 bands of 1 nm width, with 10^6 photons. Because this was very demanding in terms of RAM, the simulations were split in 4 intervals of 525 nm each, meaning that a total of 14,400 simulations were performed.

An average spectral profile of L_W was generated for each scenario, and the corresponding standard deviation was derived from the 10 runs per scenario. In the case of the reference scenarios, this process included the estimated photon noise for each band needed for the estimation of SNR.

To estimate $\sigma_{ref}(\lambda)$, we accounted for all potential combinations of the 10 runs per scenario, including both litter cases and reference scenarios. This means that $\sigma_{ref}(\lambda)$ was estimated using a total of 100 points (10x10) with respect to the mean value of the differences, as defined previously.

The average difference between reference and litter scenarios informs about what spectral bands are affected by litter presence and how much. Note that, in this case, we are not aiming for the classical remote sensing process, in which we hope to maximize contrast by combining absorption features of target and background with reflection features. Given the fact that water is a strong absorber of NIR and SWIR, it will be difficult to find spectral areas in which water reflects more than plastics. Instead, finding spectral areas where plastic has a noticeably larger reflectance than water can serve for the purpose of detection.

Finally, the SNR_r computation was used to determine those bands having a meaningful signal and potential candidates for a ML detection algorithm. In section S3.1.2 and section S3.1.3, a summary of these findings is provided.

S1.3. Mission Concept and way forward

S1.3.1. Specifying a potential EO4ML Mission

One of the key elements for the design of a mission concept are the so-called Mission requirements, already discussed in the present document. Based on such information, we devised a set of experiments and modelling exercises with the goal of narrowing down the options and providing a more realistic approach to a potential EO4ML mission.

The following sections translate our data into the definition of such an EO4ML mission. The specifications here provided are not necessarily definitive, but only a starting point that shall be revisited with further studies. Indeed, we have identified a number of activities that could help to better define the mission concept and approach a final instrumental setup, in particular with regard to the retrieval methods, which are at the core of determining the required sensitivity in the spectral bands for ML observation.

The proposed instrument is not the only one that could play a role in the ML remote sensing, but it is considered the most promising. A major limitation of the passive multispectral sensors is the presence of clouds, which block the transmission of electromagnetic radiation over most of the spectrum, from UV to SWIR. Microwave-based techniques (*e.g.*, Synthetic Aperture Radar, SAR) have no such limitation, although they present other important difficulties and caveats[42]. Here, we decided to focus on a passive superspectral approach; however, the ideal EO4ML solution would involve a combination of instruments. Given the potential synergies between available technologies, research and exploration of alternative methods should be addressed in future work.

S1.3.2. Detection principle and radiometric requirements

We aimed first to define the minimum surface coverage from which the presence or absence of ML in a pixel can be asserted. Potential indices were explored by combining spectral bands and determining the relationship between the

fraction of litter coverage and the accuracy required by each index for detection. In addition, we examined the link between the accuracy of spectral indices and radiometric requirements of the spectral bands. Eventually, we provided a very first estimate of the adequacy of current or future missions for the proposed detection approach. The overall approach is presented in Fig. S5.

The paradigm of detection is based on identifying anomalies in the image. These anomalies can be geometric (such as finding a pattern), radiometric (such as a given absolute level of radiance, a specific spectral signature, or a relative level of radiance, *i.e.* the contrast).

Detection was performed by applying a threshold on the raw or processed image, and the detection performance was achieved by compromising between the detection probability and the probability of false alarms. The two main challenges of ML detection are as follows:

- False alarms: ML can be surrounded by other similarly reflecting matters such as white caps, foams, algae and natural debris (driftwood, reeds and others).
- Background sources: a weak signal coming from low ML concentrations can be mixed with parasitic background sources such as atmospheric contributors. In detection terms, this leads to poor signal-to-noise ratio, meaning that even in the case of good SNR, detection might be prohibited.

As a consequence, the selection of the spectral bands, the radiometric performances and the dynamic range will be the driving requirements for an optical instrument dedicated to ML detection.

Definition of sample spectral indices for ML detection

To support the investigation of a mission concept, a set of spectral indices were explored according to the set of spectral bands that were identified as potential observing bands. These indices are not intended to be the final approach for the retrievals, rather than to help understanding the detection limits. Other indices and/or methods could be investigated in the future for a more optimal retrieval, including spectral unmixing. Nonetheless, a preliminary set of spectral indices was identified as the most relevant based on the knowledge acquired in the experimental exercise. We propose composite optical indices from the spectral characteristics of plastic litter with respect to sea water, using results for SZA=45º, as they appear the most promising in terms of ML detection according to the simulations.

The first proposal of normalized indices, T_1 and T_2, relied on the set of bands identified during the modelling (Table S9). These bands indicate the presence of the usual floating plastics and also correspond to local optima for atmospheric transmittance, *i.e.*, B1 and B2 are within a shallow peak of transmittance between 1400 and 1800 nm, while B3 belongs to the next cluster of peaks starting above 2000 nm. The indices are:

$$T_1 = \frac{B1 - B2}{B1 + B2} \qquad T_2 = \frac{B3 - B2}{B3 + B2}$$

where B1, B2 and B3 indicate in the formula the reflectance values in the associated bands. Both indices pivot around B2 because it is identified as a local minimum for the tested polymer, and still more reflective than water. However, the reflectance increases significantly in all the cases towards B1 and B3 that yield values that are well distinguishable from plastic-free seawater, whose reflectance is very close to 0 in these bands. A next step can be to further combine T_1 and T_2 to create more complex indices. A Plastic Marine Litter Index (PMLI), estimating the existing differences between B2 and the other two bands, could be defined as:

$$\text{PMLI} = T_1 + T_2$$

Table S10 summarizes the value of the indices for the three tested polymers and seawater free of plastics, using three different concentrations. Both T_1 and T_2 have a distinguishable value when litter is present. In the case of T_2, values for plastic presence can be more than 10 times higher than those for seawater free of plastics, which is a very encouraging result. This sensitivity remains even for very low values of concentration (0.1%). However, T_1 drops quickly, which presents some limitations. Nonetheless, such information could be useful for a potential spectral unmixing and to help to discriminate major components in the plastic fraction of a given pixel. By checking the ratios of each component of the index with respect to the reference (Table S11), it is possible to verify that T_2 is the most sensitive (in relative terms) component of the index to the presence of plastics.

Interestingly, the ratio T_1/ T_2 is also of value here, as these ratios show high sensitivity to ML presence, even at low concentrations. Nonetheless, limits for these ratios to be valid will rely entirely on the relative impact of the uncertainties on these bands during measurement. Note that, in case of sun glint, the values of these indices can be expected to remain confusing.

Additional bands could be required for further discrimination of ML with respect to potential false positives. For instance, the bands in Table S12 could be involved. They shall be complemented with those bands for atmospheric correction development over coastal areas and open ocean. These can be derived from Sentinel-2 and Sentinel-3 missions, for example, and their requirements would be similar for ML applications.

Moreover, several other bands will help in the effort of plastic detection and quantification. Table S13 includes another two bands that could be used to constrain ML retrieval, as well as to build dedicated spectral indices.

Relationship between plastic fractions and required index accuracy

The identification of the litter type and their quantification corresponds to a next step and would deserve a dedicated study on the subject. Here, as a starting point, we focus on the detection ability of the instrument. The ability to detect littered scenes (or distinguish them from clean scenes) was assessed through two values:

- the false positive (Fp), erroneously flagging a zone as polluted.
- the false negative (Fn), erroneously flagging a zone as clean.

These wrong assignments are linked to the existing overlap of the Gaussian distribution of the measurements around the true index value for each scene. A common uncertainty (σ_ρ) was considered for the measurements on both scenes. The closer the index values for clean and polluted scenes are, the smaller the uncertainty (σ_ρ) is required to be able to distinguish both populations. For that purpose, we propose to set a threshold value, $\rho_{threshold}$. The principle is that if the measured ρ is above $\rho_{threshold}$ the scene is flagged as polluted. If ρ is below $\rho_{threshold}$ then the scene is flagged as clean.

The tail area of the probability distribution over the threshold for a clean scene represents the cases for which the scene is wrongly flagged as polluted (because $\rho > \rho_{threshold}$). Similarly, the tail area of the probability distribution below the threshold for a polluted scene represents the cases for which the scene is erroneously flagged as clean (because $\rho < \rho_{threshold}$).

The Probability of an erroneous flag can be represented as a function of the threshold value. If $\rho_{threshold}$ is lowered the more scenes will be flagged as polluted at the cost of more false alarms. On the other hand, if $\rho_{threshold}$ is increased less scenes will be flagged as polluted, but the ones flagged will be more reliable.

We choose here to set the threshold detection at the mean value of ρ_{clean} and ρ_{litter}:

$$\rho_{threshold} = \frac{\rho_{litter} + \rho_{clean}}{2}$$

With this formulation, the probability of false positive is equal to the false negative one. Assuming as a rule of the thumb that we could accept to miss 10% of the polluted scenes, this means accepting a false negative of 0.1. Once this level and the threshold are set, the required accuracy can be obtained on ρ. Table S14 presents the required accuracy (one σ) to meet the false negative level for two concentrations of LDPE.

Relationship between index uncertainty and SNR for spectral bands

We derived the radiometric requirements for the selected spectral bands based on the previous discussion. We focused on the index T_I, however, the present approach remains valid for any other index, therefore we labelled the index generically as ρ.

Let's recall the typical definition of ρ:

$$\rho = \frac{B1 - B2}{B1 + B2}$$

The first step consisted in establishing the relation between the uncertainty on the index (σ_ρ) and the SNR of the involved bands.

$$\sigma_\rho = \left|\frac{\partial\rho}{\partial B_1}\right| \sigma_{B1} + \left|\frac{\partial\rho}{\partial B_2}\right| \sigma_{B2}$$

That is based, on ρ expression:

$$\sigma_\rho = \frac{-2}{(B_1 + B_2)^2} [B_2\, \sigma_{B1} + B_1\, \sigma_{B2}]$$

Assuming the uncertainty on both spectral bands (B1 and B2) is similar then $\sigma_B = \sigma_{B1} = \sigma_{B2}$

$$\sigma_\rho = \frac{-2\,\sigma_B}{(B_1 + B_2)^2}[B_2 + B_1] = \frac{-2\,\sigma_B}{(B_1 + B_2)}$$

Assuming signal levels B1 and B2 are close, the average value was considered as representative of the signal level in both bands, *i.e.*, (B1+B2)/2 = $B_{average}$ ~ B

$$\sigma_\rho = \frac{-2}{B_1 + B_2}\sigma_B = \frac{\sigma_B}{B_{avg}} = \frac{1}{SNR}$$

Compatibility with existing or future instruments

From the presented formulation, we obtained the SNR required for the selected spectral bands B1 to B3 (Table S15). These radiometric requirements can be compared to the performances of MSI on Sentinel-2 (Table S16).

Selected B1, B2 and B3 bands (respectively at 1615 nm, 1782 nm and 2031 nm) are not part of the S2-MSI band set. Therefore, we carried out a rough assessment of what would be the performances for such bands by interpolating the performance of MSI for the two first models in orbit. The SNR computation was scaled with the spatial sampling, the spectral bandwidth as well as the reference radiance (using the reference scene). Band B1 is the most reliable since it is close to band B11 on MSI (1610 nm). The two other bands are further away from MSI bands (Table S17); the associated results must therefore be considered carefully.

The adequacy of the MSI extrapolated performance with the derived SNR requirements for ML is presented based on the T_1 data for LDPE plastic type. Two concentrations were considered 1% and 0.1% as well as levels of acceptable false negative. In order to assess what could be the compliance of an improved instrument of the same type (multispectral imager), we assumed an improvement of the SNR by a factor 2 with respect to the current generation, consistent with current roadmaps on this type of instrument. In addition, note that we assumed here the same orbit as S2-MSI, *i.e.*, close to 800 km. Lowering the orbit would obviously improve the SNR performance while keeping the same spatial sampling. The relative evolution of SNR will follow that of the altitude (*e.g.*, lowering the orbit to 600 km would result in a 30% increase in SNR).

A first conclusion of this first assessment is that it would be possible, with a superspectral instrument similar to S2-MSI with dedicated ML bands, to detect scenes with plastic litter with a spatial sampling of 10 m for a 1% concentration.

Improving the spatial resolution to 10 m, while maintaining this level of detection, is possible with an improved instrument (typically the next generation of MSI). The radiometric requirements associated with a plastic concentration of 0.1% do not appear to be consistent with a reasonable rate of erroneous detections with an MSI-like instrument. This is mostly due to the low radiance of the scenes.

A further step could be first to refine the previous analysis (questioning and consolidating assumptions) and then to explore more and possibly more complex indices, *e.g.* involving more spectral bands in order to lower the required SNR.

S2. Supplementary Methods for Proof of Concept

The work done here revealed that S2-MSI has partial capabilities to observe floating litter. Whereas the spectral bands of this instrument are not ideal, and spatial resolution in some key bands is far from the optimal values, a partial match was evident. A number of existing papers have dived into different methods to exploit images from S2-MSI for plastic monitoring, quite often supported by Machine Learning[9,43-46].

However, in light of our previous results on the feasibility of an EO4ML mission, it is unlikely that S2-MSI can actually distinguish plastic by itself, rather than a family of floating substances with look-alike spectral characteristics in the S2-MSI bands. This conclusion is also supported by other studies[47,48], which point that accurate plastic detection with this sensor is unfeasible, and that many of the previous reports do not provide a comprehensive description of the impact of such limitations in their results. Even if we target here floating plastic as an indicator of ML, any plastic detection based on S2-MSI acquisition will be affected by other floating matter, so a proxy approach will be more suited to real-world applications when dealing with S2-MSI data.

In this section we describe how the knowledge gained in the optimal mission conceptualization was applied to define a specific spectral index for the S2-MSI of the EU Copernicus Sentinel-2 mission, based on the compatibility of the S2-MSI bands within NIR and SWIR regions. We then described the processor built to systematically identify those aggregations of pixels with positive spectral detection resembling filament-shaped patches (LWs). In the following, we report the post-processing supervision of the image clippings of each candidate filament automatically detected by the processor. Finally, we present how we implemented the detection processor to our ROI.

S2.1. Definition of the PoC data processor

S2.1.1. Atmospheric correction

The atmospheric correction (AC) schemes compensate for the effects of scattering and absorption by gases and aerosols in the atmosphere. Correcting the images from the atmospheric effects modify the magnitude and shape of the spectral reflectance signal as the additive atmospheric effects are removed. Unfortunately, AC impact on the detection of plastic targets is still not well understood. In 2022, Hu[47] highlighted that AC can be extremely damaging when trying to detect ML (and plastic litter), and strongly recommends to not apply or do it in a very limited way, due to the following reasons (sic):

- To retain most spectral shapes (as opposed to magnitudes) from the at-sensor (TOA) reflectance, and to avoid potential errors caused by AC (*e.g.*, aerosol assumptions).
- To avoid occasional false masking problems over bright targets (i.e., being falsely treated as clouds).
- The spectral distortions demonstrated by Hu[47] are due to pixel mixing (water and other endmembers), mixed band resolutions, and band-to-band registration errors, having nothing to do with AC.
- A full AC may induce additional errors due to selections of the dark targets and/or assumptions in spectral shapes of the dark targets or aerosol types.
- Different AC approaches may result in different surface reflectance spectra of the same ML-containing pixels[6], which makes the validation of the reflectance signal of mixed pixels at sea complex.

Given the relevance of the optical signature of plastic polymers in the longer wavelengths, the AC of choice had a significant effect on the classification scheme developed for the PoC processor. However, there is currently no robust uncertainty budget on the AC data processors, which hampers any optimization[49]. The only work that addressed this issue in the context of ML detection was done by Topouzelis and co-workers[6]. They compared ACOLITE and Sen2Cor atmospheric corrections, including also uncorrected data in the analysis. Topouzelis and co-workers concluded that differences between AC options are higher than the magnitude of the signal to be observed, so AC will play a major role in the overall performance of any solution. Despite this, ACOLITE seems to be the preferred option by the community, probably because it is the most pragmatic solution. It is noteworthy that ACOLITE does not keep the native band resolution of the S2-MSI L1c when applied, generating a regular output to 10 m spatial resolution, which is one of the causes of the spectral distortions referred to by Hu[47]. In the present work, we carried out a new and extensive comparison of AC approaches in order to identify the best approach for the PoC processor.

Atmospheric correction intercomparison exercise

We performed a consistency analysis of three atmospheric corrections, namely:

- Sen2Cor, implemented within the Sentinel-2 L2A ESA processing chain[50];
- ACOLITE, postulated by some studies as more interesting for ML applications[51];
- MEETC2, developed by ACRI group on behalf of ESA and that proved to produce a more consistent inter-pixel solution[52].

The intercomparison exercise was done considering the following assumptions:

- Plastic polymers have a consistently higher reflectance than sea water for the bands in the region 350 to 2500 nm under no-glint conditions.
- Detection of ML requires a contextual interpretation, *i.e.*, a spectral contrast between the LW and the surrounding surface of the ocean.
- An AC using information in NIR and SWIR reduces such spectral differences between water and LWs, thus making it harder to identify by any data processing.

According to that, the most interesting AC would be the one that maximizes the relative difference between confirmed LWs or artificial targets and that of the surrounding water in the bands of more interest for the detection. This can be mapped using the relative differences between the reflectance for the key bands for identifying LW targets and sea water:

$$diff = 100 \left(\frac{r_{target} - r_{water}}{r_{water}} \right)$$

The higher this relative difference, the larger the contrast. The corrections yielding the largest values are the ones more interesting to help identify LWs. This was one of the conclusions derived from our definition of an optimal EO4ML mission concept: seawater has no bands where water is more reflective than plastic under normal observational conditions.

The analysis was developed over 6 locations and LWs previously identified. MEETC2 systematically overperformed the other atmospheric corrections for our purposes, being followed by Sen2Cor, with ACOLITE yielding the least contrasts for the study cases.

The results were mainly constrained by band B8A. Indeed, this is the main band in many commonly used spectral indices such as NDVI, NDWI, FAI and others that use such indices as a reference to build detection algorithms. Table S18 shows an example of the scoring and results of the analysis. The bands had different responses to the same correction. Different atmospheric corrections performed better in specific conditions and bands, with MEETC2 apparently performing better than the other two.

Approach for the PoC

The results pointed to improved performance with MEETC2 correction. A few drawbacks were highlighted:

- MEETC2 is designed for Ocean Colour (OC) applications. Therefore, its main interest is to properly correct the relevant bands for such applications.
- ACs artificially alter the SNR of the bands affected, quite often increasing the noise level within the image for some of the bands. This is particularly true for pixel-based correction, where no consistency is expected between a given pixel and its neighbour, a problem substantially mitigated in MEETC2.
- MEETC2 operates over a 60 m basis resolution and uses such bands from L1C products. 60 m-family of Sentinel-2 products are an upscaling from the bands of 10 m and 20 m to 60 m by pixel merging (*i.e.*, 60 m resolution bands are of better quality than 10 m bands, at the price of reducing spatial resolution).
- Sentinel-2 products at L2A are processed with Sen2Cor, and no systematic product archive exists for the other corrections. Thus, a different choice of AC forcedly involves the additional processing step of performing the correction dynamically, with the corresponding overhead of data processing time (between 7 to 20 minutes per tile, depending on the specific AC). In addition, current ESA archives for L2A products only cover a fraction of the Sentinel-2 mission (2018 onwards), so using Sen2Cor is neither an optimal solution.

Because of the physical dimensions expected for the LWs, a 10 m resolution approach was preferred for their detection, and as discussed hereunder, a resolution over 10 m is mandatory to ensure a successful identification of LWs. Thus, MEETC2 would require an upgrade to 10 m resolution, activity beyond the objective of this study, and a

more systematic analysis of the output level of noise for the relevant bands over a broader set of study cases. This point could be investigated as a potential future improvement of the PoC processor.

In addition, the methodologies tested for LW detection are sensitive to noise levels within the bands employed for the detection. This is to be expected, since the signal is often very faint and is usually the result of pixels containing only a fraction of their surface area with LW. Thus, increasing noise levels is significantly harmful for the detection, both by augmenting the presence of false positives and reducing the chances of true detections.

Finally, as a pragmatic point of view, one of the requirements for the PoC processor was to have a great performance, so that large batches of Sentinel-2 tiles can be processed in reasonable periods of time. The use of an AC different from Sen2Cor imposes an additional time overhead that will cramp such efforts, limiting the generation of results.

Because of the above reasons, we decided to operate using only L1C data files without applying any atmospheric correction. As we targeted to detect anomalies at the sea surface, and not much aiming to quantify the observations, this approach suffices to comply with the objectives of the study. The downside is that, to accommodate for the use of L1C products, specific thresholds must be developed in accordance with TOA reflectance values for any spectral index.

This impact is not well understood and, another possible approach, not taken here but of value for future work, would be to consider only the Rayleigh effect contribution. That was the proposition of Hu[47,48], which deserves to be explored in future evolutions of the processor.

S2.1.2. Cloud masking for the PoC

A challenging aspect in the detection of LWs relies on the false positives generated by clouds. Sen2Cor´s cloud masking is a sophisticated process that involves multiple steps, comprising the computation of several indices, thresholds and even a Neural Network. Bands must be tested back and forth, and different decision criteria need to be used at different steps.

The Sen2Cor SCL algorithm classifies the pixels as categories, including thin cirrus (mainly driven by band B10), and high and medium probabilities of clouds. However, this algorithm is not good enough to properly identify the clouds, and multiple thin clouds are completely missed despite being very obvious in the True Colour image by eye (Fig. S6). This issue was reported by Tarrio and co-workers[49], who performed an inter-comparison exercise of 5 cloud masking algorithms commonly used for Sentinel-2 MSI imagery (including Sen2Cor). According to the authors, none of the algorithms achieves a 100% accuracy in the detection of clouds, and the results vary according to the observational conditions. Based on their selection of testing sites and cases, Sen2Cor was one of the algorithms showing a larger "omission error" (*i.e.*, false negatives), even if it was the best algorithm in terms of "commission errors" (*i.e.*, false positives). This suggests that Sen2Cor is a conservative algorithm that prioritizes true detection over pixel removal. For the same reason, Sen2Cor is the most sub-optimal algorithm for the application intended in the PoC, where quality shall prime to quantity.

The main issue with the clouds in PoC resided in the lack of accuracy in finding their edges (Fig. S6, bottom panel). Essentially, edges of clouds that are not properly masked could lead to "fake" LWs. To prevent this, state-of-the-art and bespoke cloud masking is required. To tackle this problem, the proposed a cloud masking algorithm that combines several existing masking alternatives, nesting the results. As highlighted by Tarrio and co-workers[49], there is no single algorithm sorting out all constraints. Therefore, after testing available algorithms, a combined algorithm was developed, which although still not perfect, yields a much better result than any individual algorithm (see Fig. S7 for details).

It is important to note that most of the explored cloud masking algorithms exploit data prior to performing any atmospheric correction (*i.e.*, over Sentinel-2 L1C products). In addition, according to the spectral bands design for S2-MSII, band B10 (1375 nm) is specifically incorporated for the detection of cirrus and thin clouds. However, this band is not reported in the L2A official products, which means that cloud masking shall be computed always starting from L1C, regardless if an atmospheric correction is applied or not later on.

Sen2Cor Cirrus detection

Following the criteria established by Sen2Cor, a first step is to identify cirrus using the B10 information from the L1C data files. This detection consists in a hard thresholding in the reflectance values reported in such band, particularly:

$$mask_{cirrus} = Boolean\,(B10 \geq 0.001)$$

The threshold value is relatively low considering that the data precision for S2-MSI reflectance is of 10^{-4}, implying that almost any significant reflectance in the band will be due to cirrus. This filtering is rather aggressive, removing large quantities of pixels with a bright nature, whereas over land or clouds (Fig. S7a). It has, however, much less impact over water pixels and LWs. Sensitivity to bright pixels also helps to remove information from the image that can be affected by detector artifacts or glint. Our analyses show how effective B10 is in detecting dense clouds and shiny surfaces, having little impact on water pixels (Fig. S7a). Still, this filtering is not enough to cope with many of the smaller and thin clouds and still has some halo effects.

MEETC2 cloud masking algorithm

The second method applied was based on the algorithm included within MEETC2 atmospheric correction[52]. This algorithm computes several spectral indices that are contrasted against specific thresholds, combining information from multiple bands. Thus, three indices are computed in this method:

$$NDSI = \frac{B03 - B11}{B03 + B11} \qquad DSI = \frac{B10 - B11}{B10 + B11} \qquad VI = \frac{B8A}{B03}$$

The Normalized Differential Snow Index (NDSI), which is also used by Sen2Cor algorithm, is based on the normalized difference between red band (665 nm) and SWIR band (1610 nm). The Drought Severity Index (DSI) index uses differences in reflectance between B10 and B11, while the Vegetation Index (VI) was estimated as the ratio between NIR (B8A, 865 nm) and green (B03, 565 nm). These indices were used along B04 (red, 665 nm), B8A and B11 reflectance values to perform three tests:

$$Test_1 = Boolean\big((VI > 0.4)\,\&\,(NDSI > -0.2)\,\&\,(B11 > 0.07)\big)$$
$$Test_2 = Boolean\big((VI > 0.1)\,\&\,(DSI > 0.01)\,\&\,(B04 > 0.3)\,\&\,(B11 \leq 0.07)\big)$$
$$Test_3 = Boolean\big((B8A > 0.35)\,\&\,(NDSI > -0.2)\,\&\,(B11 \leq 0.07)\big)$$

The resulting cloud mask is obtained when any pixel complies with any of the selection criteria above:

$$mask_{meetc2} = Boolean\,(Test_1 \mid Test_2 \mid Test_3)$$

This filtering is less aggressive over bright areas and leaves pretty much all water pixels as valid (Fig. S7b). Land regions are significantly impacted, mainly due to the filtering associated with VI values. As MEETC2 correction is designed for Ocean Colour applications, these results are expected. Nevertheless, the MEETC2 algorithm is not enough by itself to properly filter all the clouds, being particularly inefficient for thin clouds over water.

CDI cloud masking algorithm

The Cloud Displacement Index (CDI) algorithm was proposed as part of improvements of the Fmask algorithm[53]. It makes use of the three highly correlated near infrared bands available in S2-MSI that are observed with different view angles. Hence, elevated objects like clouds are observed under a parallax and can be reliably separated from bright ground objects.

The algorithm relies on estimation of "textures" generated by this parallax effect in those three a priori highly correlated bands. As the NIR bands 7 (783 nm), 8 (842 nm) and 8A (865 nm) are provided at different spatial resolution, the 10 m band is deconvolved using an approximated Sentinel-2 Point Spread Function (PSF). For land surfaces, band 8A and band 8 are more similar than band B8A and B07 because the first pair is spectrally overlapping. On the contrary, cloud tops are more similar in bands B8A and B07 because of the high parallax between bands B8A and B08. Hence, the separation is achieved by means of two NIR ratios, defined as:

$$R_{B8A,B08} = B08 \,/\, B8A \qquad R_{B8A,B07} = B07 \,/\, B8A$$

Land surface is spatially smooth in $R_{B8A,B08}$ whereas there is more spatial granularity in $R_{B8A,B07}$. On the contrary, clouds appeared very flat in $R_{B8A,B07}$, whereas there is much spatial variability in $R_{B8A,B08}$, due to the parallax effect. It is noted that the spatial variability in $R_{B8A,B08}$ is not confined to cloud borders, but is also apparent for the centre of the clouds, which are highly structured and thus parallax effects could readily be seen.

Texture measures are effective to highlight image contrast (*e.g.*, caused by misalignment). To obtain mathematical proxies of these textures, each ratio is convolved with a focal variance filter of a given width W to both $R_{B8A,B08}$ and $R_{B8A,B07}$, yielding the parameters $V_{B8A,B08}$ and $V_{B8A,B07}$.

A variance filter is chosen as it is a simple measure of texture, which is computed in a 1-pass implementation, being cost-effective compared to more advanced texture filters like multi-channel filtering or grey level co-occurrence matrices. As the internal cloud structuring in $R_{B8A,B08}$ resembles a wave structure with alternating low and high values, a filter width of W pixels is found to be optimal to perform spatial textural aggregation such that detectability in both troughs and crests could be ensured.

$V_{B8A,B07}$ shows opposite behaviour. Clouds typically have much lower spatial contrast than in $V_{B8A,B08}$. Land surfaces typically show higher or only slightly lower contrast than in $V_{B8A,B08}$. Following this, a normalized differenced variance ratio is computed to highlight the opposing nature of $V_{B8A,B08}$ and $V_{B8A,B07}$. This new index was hereby denoted as Cloud Displacement Index CDI:

$$CDI = \frac{V_{B8A,B07} - V_{B8A,B08}}{V_{B8A,B07} + V_{B8A,B08}}$$

The values of CDI are then filtered against a threshold to identify the cloudy pixels:

$$mask_{cdi} = Boolean\,(CDI < -0.35)$$

There are two adjustable parameters in this cloud masking, (*i*) the size of the spatial window, *W*, and (*ii*) the value of the threshold used in the CDI results. In our case, after some essay/error, a window $W=10$ was found as the best value for the spatial size, and a threshold of -0.35 in the CDI found as the optimal value.

Note that these are not the nominal values initially proposed by Frantz *et al.*[53], where authors selected a window of $W=7$ and a threshold value of -0.5. The choice of W was made because often there is no specific physical criteria for the choice of the window size in those cases. However, a size of 10 pixels is equivalent to spatial scales of 100 m, which has some sense of scale. The threshold was also increased for a more conservative masking. It was verified that lower values than the selected lead to the non-detection of larger fractions of thin clouds. Another aspect to consider is that this filter will extend from the edges of the clouds a certain distance, being this distance larger as the size of the window W increases. Values larger than 10 pixels may result in artefacts around the edges of the clouds, whereas reduced values are not as good as detecting them.

This filter proved to detect clouds considerably more effectively than the previous ones (Fig. S7c). It was also quite aggressive over land areas, but still preserved water pixels, as intended.

LaSRC cloud masking algorithm

LaSRC is one of the cloud masking algorithms included in the inter-comparison by Tarrio and co-workers[49]. LaSRC is one of the most efficient algorithms for cloud masking, showing a compromise between errors by commission and errors by omission. Unfortunately, the algorithm cannot be easily implemented for Sentinel-2 without introducing a considerably complex processing. It was integrated within the atmospheric correction of the same name, making use of climatological data coming from MODIS. Indeed, the cloud detection is part of the estimation of the aerosol optical thickness, which includes estimation of differences between Landsat-8 reflectance and MODIS climatologic reflectance values in certain bands.

We included one component of LaSRC for the PoC processor, which is based in the computation of a spectral index called $NDVI_{mir}$:

$$NDVI_{mir} = \frac{B08 - 0.5\,B12}{B08 + 0.5\,B12}$$

The novelty of this index is that it looks for spectral anomalies between band B08 (842 nm) and B12 (2160 nm). It uses the band with the longest wavelength within Sentinel-2 and, as for other indices used in cloud masking, it highlights variations of the reflectance values, this time comparing NIR and SWIR. This mask is obtained by computing the $NDVI_{mir}$ index and applying a threshold:

$$mask_{LaSRC} = Boolean\,(NDVI_{mir} < 0.5)$$

It was applied over those pixels with a normalized difference less than 0.5. This masking shows little impact on thick clouds. Instead, it is much more efficient to detect thin clouds than any of the previous algorithms (Fig. S7d). It was also better at spotting small clouds, being an appropriate complement to the other masks.

PoC cloud mask

Once the four proposed cloud masks are applied, the final mask results from the merging of all of them. This is done by multiplying binary the masks, *i.e.*, we masked a given pixel whenever it is detected by any of four individual masks:

$$cloud_{mask} = Boolean\ (mask_{cirrus}\ |\ mask_{meetc2}\ |\ mask_{cdi}\ |\ mask_{LaSRC}\ )$$

This cloud mask is stored and used prior to producing further steps in the processing chain. As expected, the merged cloud masking was more efficient than either method alone (Fig. S7e), although some small areas with thin clouds are still not properly masked.

It is worth mentioning that the PoC cloud masking may be too aggressive in some cases. However, it has the benefit of significantly reducing false positives associated with clouds, increasing the ratio of success in the LW detection.

S2.1.3. Land masking

In official S2-MSI L2A products, land masking can be achieved by using the SCL classification, in addition to some spectral indices to separate water from land. In particular, GNDVI index was used in addition to an SCL filter based on values between 3 and 5, inclusive (corresponding to vegetation and non-vegetation categories). This approach, however, proved to be inefficient and prone to cause some problems for various reasons. One of the main ones is that SCL classification is exclusive, *i.e.*, one pixel cannot pertain to more than one category. This is because SCL is computed from the spectral information in the image, and therefore, multiple categories over the same pixel are difficult to achieve, not always masking properly the data, and quite often having issues in the limits between classes.

For the present version of the PoC processor, a different approach was proposed, which exploits the functionalities of the ESA SNAP software[54]. SNAP software has many utilities executable for operational purposes, through calls to its modules using command-line accessible functions. One of those functions calls SNAP to generate an ad-hoc land mask of 30 m pixel resolution using information from the NASA Shuttle Radar Topography Mission (SRTM)[55]. This international mission provided digital elevation models on a near-global scale from 56°S to 60°N to generate the most complete high-resolution digital topographic database of Earth prior to the release of the ASTER GDEM in 2009. SRTM consisted of a specially modified radar system that flew on board the Space Shuttle Endeavour during the 11-day STS-99 mission in February 2000.

SRTM radar data includes a variety of products, being SRTM 3-arcseconds (90 m) and SRTM 1-arcseconds (30m) within the state-of-the-art of globally remote sensed topography. SNAP software called by a command line to the module 'gpt' is able to map SRTM 3-arcseconds into any Sentinel product, generating a land mask of 30 metres resolution. The mask is downscaled from 90 m to 30 m using a band as template, at the choice of the user. For this operation and application, B02 (442 nm, blue) is recommended and so was used in the PoC processor.

SNAP connected to an ESA database to identify the SRTM tiles that were requested. Such tiles were downloaded into the processing system and the mask computed using such information. This process can be sped up by pre-downloading all the SRTM tiles (30 GB) and storing them in the default folder that SNAP includes for such purposes. It also has the advantage that it will work even if there are problems with the ESA servers associated with SNAP.

The SRTM 1-arcsecond product was also accessible by SNAP. Dataset (approx. 270 GB) is accessible via NASA Earthdata portal (https://earthdata.nasa.gov/). We considered that handling high-resolution data will be significantly slower in comparison to the gain in improving the accuracy of the land mask generated this way. The error introduced by masking 30 m pixels was considered minor because it will affect principally to the immediate pixels along the coast. In fact, the detection of LWs in this narrow water strip adjacent to the coast is a huge challenge due to the surf frequently generated in this zone, and the fact that the seabed can become visible, which may trigger false positives.

The proposed land mask $land_{mask}$ effectively removed land masses from the image. The expected inaccuracy of the land mask at the 30m scale went in two directions: missing land, or covering water. In other occasions, the age of the data was sufficient for not considering variations of the coastal line, either by human structures or simply erosion/accretion. This type of mask had also the issue of not fully considering tidal effects.

S2.1.4. Detector footprint masking

An aspect that had to be considered was the effect of the limits between detectors of the MSI instrument. Sharp reflectance variations could appear in such frontiers, leading to false positives. Because the frontiers are parallel to the orbit swath, they take the shape of parallel straight lines, raising false positives in some occasions. The problem

became apparent when computing spectral indices, during which the normalization process quite often sharpened the image. They were the result of an across-detector variation of the general reflectance of the track that did not preserve continuity with neighbour detectors. This finding pointed towards a geometric distortion of the image, probably due to slightly different observational geometries for each detector.

Fortunately, Sentinel-2 L1C products prior to October 2021 contain a built-in mask in GML format associated with each spectral band coming within the product. This mask was used to identify these frontiers by means of plotting the polygon indicated by the arrays of coordinates included in the mask.

The main limitation of this built-in mask is that it provides the mathematical line defining the frontier between the detectors, but does not account for the frontier pixels contaminated by the edge effect. Thus, this mask had to be broadened to cope with those extra pixels. For the PoC processor, the polygons were traced and referenced to the image, and the resulting one-pixel lines were coarsened to 10 pixels radius to more effectively remove such problems, and resulting in a so-called df_{mask}.

S2.1.5. Filtered data

Once all the previous masks were processed, a single global mask (bad_{pixels}) was produced and applied to the product. The purpose of this operation was to mask all possible erroneous data prior performing LW detection, as explained in later sections. The merging was done by simply multiplying the resulting binary masks, or by Boolean selection of each filter, removing all pixels corresponding to clouds, land or faulty due to the footprint of the detectors:

$$bad_{pixels} = Boolean\ (cloud_{mask} \mid land_{mask} \mid df_{mask})$$

S2.1.6. Additional metadata

In addition to the masking, certain image parameters were calculated to support results validation and analysis of performance, which were critical in later phases of the processor. All these general parameters were computed from the masks generated in previous steps.

Portion of the image within the frame

Sentinel-2 tiles, before geometric correction, have a fixed size of 10980 x 10980 pixels (around 110 megapixels), and each Sentinel-2 orbit is projected over these tiles. This mode of operation can result in some tiles having only a fraction of the surface with valid data.

To enable a quick scan over output files and potentially discarding images with less than a given percentage of coverage, this figure was computed and stored:

$$\%\, coverage = 100\ \frac{n_{valid}}{n_{total}}$$

where n_{valid} is equal to the amount of non-zero value pixel in an image, using a band of reference at 10 m resolution (namely, B02), and n_{total} is equal to the total amount of potential pixels (*i.e.*, 10980 x 10980).

Land fraction

The use of the land mask allows for estimating the percentage of the image that is actually ocean. This fraction was estimated as the ratio between the number of pixels flagged as land over the total of valid pixels as valid:

$$\%\, land\_fraction = 100\ \frac{n_{land}}{n_{valid}}$$

where n_{valid} is equal to the amount of non-zero value pixel in an image, using a band of reference at 10 m resolution (namely, B02), and n_{land} is the total of pixels flagged as land by the SRTM land mask.

Cloudiness

A common parameter reported in optical data processing is the degree of cloudiness on the image. This is typically computed as the percentage of pixels flagged as cloudy versus the total number of valid pixels. In our processing, a small modification was done, computing the cloudiness as the percentage of cloudy pixels taking place only over

ocean waters and after discarding the detector footprints. In other words, cloudiness was reported only in relation to the valid pixels over water. This figure was computed as follows:

$$\%\,cloudiness = 100\,\frac{n_{cloudy(ocean)}}{n_{valid(ocean)}}$$

where $n_{cloudy(ocean)}$ is the number of pixels flagged as cloudy masked by land and footprints, and $n_{valid(ocean)}$ all the valid pixels over water, masked also by land and footprints.

S2.1.7. Windrows Spectral Index detection

An original spectral index was defined to support the spectral detection of LWs. The index, called Windrows Spectral Index (WSI), was intended to provide a classification of image pixels, with and without plastic-like spectral profiles, minimizing the effect of potential sources of interference. This comes on top of the extensive pre-filtering applied in the previous processing steps, also aimed at minimizing false positives.

The foundations of WSI rely on the knowledge accumulated during our previous laboratory and modelling experiments, namely:

- the in-situ measurements of plastic polymers using ASD;
- the radiative transfer modelling experiments;
- the Sentinel-2 spectral characterisation of agricultural plastic roofs as spectral reference;
- The Sentinel-2 spectral characterisation done over artificial ML targets deployed in the experiments in waters off Lesbos Island[5,6,56];
- the drone multispectral observations also acquired during the experiments referred above;
- the cases of windrows validated by image inspection over areas where in-situ observations indicated their presence, and known to contain plastic and/or other human-made litter, like the MARIDA database[7].

Note that the S2-MSI pixels employed here were identified in the aforementioned sources but collected at L1c (TOA) level. This means, for instance, that we gathered the same images and locations indicated in previous studies[7,8] but we did not apply ACOLITE AC to them.

The need for a new spectral index is also partly due to the limitations of existing approaches reported by Hu and coworkers in several works[47,48,57]. These works highlight the following issues:

- ML is composed of different materials and substances that can largely vary depending on the specific regions and circumstances that originate it, so it is very difficult to derive a particular singular spectrum based on satellite observations from a very limited set of locations.
- The lack of demonstrated efficient spectral unmixing techniques operating in S2-MSI data, at least for the major components of floating matter, precludes certainty of pixel-level classification for floating plastics.
- The use of S2-MSI has specific limitations due to both its spectral resolution and bands, and the presence of spectral distortions. Those are caused by varying spatial sampling, with a mixture of 10, 20, and 60 m bands being interpolated to 10 m, which results in important spectral anomalies.
- Current atmospheric corrections introduce significant spectral distortions and/or errors that can impede a proper ML determination. Therefore, such analysis is best performed at TOA rather than at BOA.
- There is insufficient knowledge regarding the sensitivity of current spaceborne sensors to the presence of floating matter, and even less for ML and floating plastics in particular.

The developments of the FDI index[8] and PI index[58], the two most broadly-cited spectral indices for plastic in the literature, did not take into account the above issues. In the present work, a particular approach was developed to provide a more generalised solution for litter detection, even if it imposes a loss of specificity, which will require a greater effort in data screening and post-processing. First, the approach will work at TOA and make use of existing knowledge on the spectral signal that ML has in S2-MSI measurements. Accounting for the potential effect of spectral distortions reported in the literature, we tried to maximize the detection of floating plastic by using its spectral singularities on the NIR and SWIR regions, avoiding the problem of the colour-varying dependence of floating litter.

The most promising solution to cope with ML monitoring needs requires exploiting the understanding of spectral anomalies on the sea surface[47], and working on the idea of a proxy (LWs) rather than on direct plastic detection. As derived from our radiative transfer modelling, plastic litter, and by extension ML, has a specific positive reflectance in the NIR/SWIR bands which can be exploited to separate it from seawater, as the later has a strong absorption on

these spectral regions. While separation from other classes will require more information, a priori, it would be plausible to use S2-MSI bands to detect floating litter on the ocean surface[47].

We approached this by analysing different classes of floating matter and/or looks-alikes in S2-MSI data that could generate false signals. The resulting index was less specific and susceptible to generate detections other than our primary target. Thus, we included additional layers in the processing chain to minimise false positives and restrict detections to LWs. To ensure our focus on LWs, we combine the spectral index with a contextual filter based on the geometry of such structures (section S2.1.8), and a further filtering was applied during post-processing (sections S2.2.2 and S2.3.3) to ensure that only LWs with high ML density were preserved for the PoC analysis.

Marine litter spectral characterisation

The dataset reported above served to identify systematic patterns in the spectral signatures of plastic litter and sources of interferences (Fig. S8). The spectral profiling using native S2-MSI images was the basis for the creation of WSI. While some pixel categories (*i.e.* ML and other ocean surface components) show similar characteristics in individual bands, it is expected that globally they will become sufficiently distinctive, at least for ML. For instance, overall litter reflectance is significantly higher than ocean water reflectance, along the entire spectral range from 350 to 2500 nm. This was already reported in previous studies[59] and confirmed by our experiments. There is a clear diving peak around 945 nm (B09) for ML, between two maximums at 865 nm (B8A) and 1610 nm (B11). Those three spectral features can be combined with additional bands in NIR for the isolation of target pixels. None of the other classes present such spectral patterns, even if "mud" and "thin clouds" categories share one side or the other. This spectral pattern was found in all datasets referred previously, being a consistent feature of ML that potentially contains plastic. Interestingly, this particular spectral pattern enables the unique detection of LWs in many cases, without need of additional external spectral information. This represents a clear processing advance, which has not been accounted for in previously published reports. Waters with a high sediment load may yield higher reflectance values in NIR bands, but they do not in SWIR bands. By playing with this particular behaviour, contrary to ML, it is possible to separate the two classes and prevent WSI from confusing sediment load with ML (Fig. S8). Likewise, seawater can be easily separated using purely NIR/SWIR bands, as well as different types of clouds that might have been left as residuals from the cloud masking procedure.

Normalized Spectral Index Confusion Matrix

To better address the best options to identify LWs by means of a spectral index, a spectral profile was reconstructed as shown for each potential spectral class, and pairwise normalized differences were computed for all bands, using all possible permutations. Those permutations enable to obtain a matrix of normalized indices of size *n x n* obtained from paired bands:

$$\text{NI} = \left(ni_{i,j}\right)_{nxn}$$

where NI is the Normalized Spectral Index Confusion Matrix, and the index ni is computed as:

$$ni_{i,j} = \frac{band_i - band_j}{band_i + band_j}$$

The values of the indices were then visualized in the shape of a confusion matrix to better interpret the spectral information associated with each class. These matrices contain all the potential normalized indices that can be obtained combining Sentinel-2 bands by pairs, helping to understand the bands playing a major role in each class. Note that all diagonal elements where band $i = j$ will be meaningless (value 0). Additionally, the matrices are negatively symmetric along the diagonal, as the same bands are used in the symmetric positions but in opposite order.

To learn on how these matrices result for different spectral end-members, we cropped pixels over various Sentinel-2 images including the following classes: seawater, thick clouds, thin clouds, sediment-rich waters (muddy waters) and ML. The latter was obtained from the Sentinel-2 images contained in the MARIDA dataset[7].

The results for ML showed that bands B11 and B12 when combined with band B8A provided the most information, followed by the combination with band B09. Bands B09 and B12 showed that the spectral contrasts increase for these bands for ML as we move towards NIR and SWIR bands, even if values are still high for bands in the visible region of the spectra. This is telling us that spectral sensitivity to ML detection is higher when using combinations of NIR and SWIR bands. This result matches perfectly the current knowledge about spectral features associated with ML and plastic litter[3,21,22,47], although not as much in-depth analysis in this regard has been previously provided.

However, to properly identify the combination of bands offering the best opportunity for detection, it is not sufficient to have characteristic features within the confusion matrix; such information must also be separable from the one associated to the other spectra classes. Comparing rows in confusion matrix, we confirmed that "mud water" pixels present overlapping behaviour in the matrix for the row associated with band B12, but not for the band B09, where ML stands out, and especially when combined with B8A. Equivalently, we verified that thin clouds share spectral features associated with the normalized indices for B09, but not for B12 where ML also stands out, especially when combined with B8A and B09, correspondingly. Thick clouds are particularly easy to distinguish, with no overlapping patterns except when combining B12 and B09, which could trigger some false positives if chosen. And practically no confusion with water pixels can take place when the indices are extracted from NIR or SWIR bands, which is an excellent result, as this implies separating floating matter from water is possible with a high degree of certainty.

The analysis concluded that the best a priori Sentinel-2 bands to detect LW are B08, B8A, B09, B11 and B12, namely 865 nm, 945 nm, 1610 nm and 2190 nm, respectively. Nevertheless, the normalized spectral indices only help to identify where the discrimination information lies, but the final solution does not necessarily involve the use of these normalized indices.

Building the WSI index

Based on the above selected bands and considering the spectral geometry, the presence of ML could be determined by the relative spectral slopes between B09 and B11, B08 and B07, and B8A and B09. Slope between B11 and B12 was excluded because it was also present in both types of clouds (Fig. S8), and therefore, it might induce false positives. B07 was added because it has a certain degree of significance according to the matrices and can be used to compute a slope of opposite signs to the other pixel classes when combined with B08.

From this information, the following criteria were used to define the index:

a. Try to ensure that the index is positive whenever ML is present, being negative otherwise;
b. Have into account the "poor" spectral resolution of S2-MSI, considering the spectral distance existing between the involved bands;
c. Avoid that overall absolute differences in brightness between bands can mask relative differences.

The last point above (c) is what explains the 0.5 factor used in the $NDVI_{mir}$ index for cloud masking. Without that factor, the normalized index with bands B08 and B12 would not yield suitable results, as B12 overall values are systematically higher than B08 reflectance, and thus, driving the normalization. Because of that, a not normalized solution was selected here, mimicking the approach of the known Floating Algae Index (FAI), which does not normalize the reflectance, and measure the geometry of the spectrum. Therefore, three different spectral slopes were used for the overall WSI:

$$\begin{aligned} wsi_a &= 10^5 \frac{B08 - B07}{\lambda_{B08} - \lambda_{B07}} + 1.25 \\ wsi_b &= 10^5 \frac{B8A - B11}{\lambda_{B11} - \lambda_{B8A}} - 1.25 \\ wsi_c &= 10^4 \frac{B8A - B09}{\lambda_{B09} - \lambda_{B8A}} - 1 \end{aligned}$$

These three slopes are expected to be positive when ML is present, whereas becoming negative in other cases. A factor scale and an offset are applied, so to set equivalent baselines to each component. Thus, the composite index results in a Boolean comparison:

$$\mathrm{WSI} = Boolean\left((wsi_a > 0) \,\&\, (wsi_b > 0) \,\&\, (wsi_c > 0)\right)$$

and thus,

$$mask_{ml} = Boolean\,(\mathrm{WSI})$$

A numerical version can also be built by combining the terms:

$$\mathrm{WSI} = wsi_a + wsi_b + wsi_c$$

In this case, WSI offers a continuous value, instead of a mask, that can only be used to fine-tune pixel classification over a given scene, as a strictly positive value of WSI does not imply each of the components is positive too, and a particular threshold must be found. However, it is more flexible and adequate for quantification of litter density, as done in this work to determine the sensitivity of the index to pixel coverage. With this approach:

$$mask_{ml} = Boolean\,(\mathrm{WSI} > threshold)$$

The value of the threshold can be obtained by computing both Boolean and numerical WSI values, and identifying the lower WSI numeral values yielding a positive (true) detection. For the case of the LW shown in Fig. S9, the resulting threshold was 3.9.

Output of the detection

To generate the raw detections of ML pixels, the PoC processor carried out all of the previous steps. WSI was computed in its Boolean form only over the valid pixels after applying the various masks, accounting for the cloud, footprint detector and land masks. The output of raw ML pixels was converted into a binary mask to be used by the deterministic object classifier detailed in the next sections.

S2.1.8. Filament contextual identification

One of the limits of detecting ML using Sentinel-2 is that it has to be aggregated in dense patches to obtain a reliable detection. The likelihood of detection and classification increases with the litter density in these patches. Interestingly, sub-mesoscale convergence structures, the windrows, can show litter densities up to 10,000-fold higher than in their surroundings[11].

Litter windrows (LWs) typically take filamentous shapes of a few metres wide and from tens to thousands of metres long. However, litter density along the LWs can be highly variable, often showing discontinuities[11,12]. In this section, we describe the deterministic method used to automatically detect filaments on the binary images after applying WSI for spectral identification of positive pixels. The WSI-derived detections contained noise, or false detections, as a consequence of the spectral limitations of the data processor used in the PoC. To better separate LWs from that noise, an additional contextual classifier was needed. Therefore, the final detections are the result of both spectral and contextual classification, which makes this exercise unique to date.

Our approach for the filament contextual classification aimed at identifying windrows having a minimum thickness, a minimum extent, and with a minimum distance between objects. In addition, if the windrows were found in spectrally noisy areas, noise reduction was applied to try to identify thick ones.

The binary image resulting from the spectral characterisation is represented by a 2-dimensional array (width, height), with the pixels being the cells of the array. Each one has X, Y coordinates and contains a *true/false* value. A *true* value indicates that the pixel is possibly pertaining to a LW, while a *false* value indicates that the pixel does not pertain to a LW. The goal was to identify *true*-pixel groupings having a filament shape, so as to separate them from the noise in the image background.

A full workflow diagram of this contextual classifier is provided in Fig. S10, and the following sections describe how it was implemented.

Definitions and conventions

Adjacency and pixel grouping

A grouping of pixels is a set of interconnected *true* pixels. A *true* pixel is considered interconnected if it has at least one directly adjacent, that is, if there is another *true* pixel among the 8 directly adjacent pixels (8-connexity). We also define the size of a pixel grouping as the number of pixels interconnected within the same set.

A grouping of pixels is considered isolated when it is not in a noisy area and is far enough away from other groupings of pixels. The distance between grouping of pixels is a relative distance which depends on the context of the image.

Bounding box

The algorithm returns as an output the bounding boxes list of the filaments identified. A bounding box is a list of coordinates that delimit the location of the pixels of a filament. In our case, such a box is a rectangle delimiting a

filament. It is defined by the coordinates (X1, Y1) of the upper leftmost point and the coordinates (X2, Y2) of the lowest rightmost point of the rectangle: (X1, Y1, X2, Y2).

Technical challenges

The identification of filaments had the following technical challenges:

- There were generally very few filaments in an image compared to the number of *true* pixels detected by the WSI. A very efficient algorithm for filament-shaped aggregations of *true* pixels is, therefore, needed.
- Some of the filaments were not ML filaments. To identify them, it was necessary to take the context into account. Therefore, the algorithm should be able to recognize shapes of filaments, but also to analyse the surrounding context.
- Filaments can be continuous, discontinuous and of different shapes.
- Filaments may be in noisy areas, which makes identification more difficult.

Noisy areas

Binary images can contain noisy areas. These are large areas containing many small clusters of pixels, less than or equal to 4x4 pixels in size and with no specific shape. These small clusters of pixels are not the physical objects sought in the PoC, being generally the result of the wave glint. This phenomenon occurs when sunlight is reflected off the ocean surface by the waves that are at the same angle as the sensor that looks at the sea. In S2-MSI images, sun glint generally happens in wave fields, as a wavy surface at 10 m resolution provides opportunities for geometries of specular reflection, which originates such local clusters. In the original image, the smooth areas of the ocean appear bright in color, and the rough areas are dark. Scintillating regions can have properties in common with the desired target, thus they are not completely filtered during the generation of the binary image.

Unwanted filaments

An image may contain filaments which do not correspond to LWs. This is explained by a multitude of objects which have properties similar to ML filaments. Clouds, land and noise were particularly problematic. The algorithm for generating the binary image should mask out all clouds and land, however, some of these elements remained.

Clouds and land were mostly eliminated. Unfortunately, this was sometimes insufficient because some edges of these objects lingered, creating unwanted filaments. They were often identifiable. Noise was only slightly eliminated. Noise has no specific shape, but in some areas, it appeared in the form of seeming filaments. They could be recognized because generally there were noise pixels scattered over great distances around them.

Identification of filaments

Filaments can be semi-rectilinear, wavy, circular or even having more complex and irregular shapes. In addition, filaments can be continuous or discontinuous. Therefore, the automatic identification of filaments had to set certain criteria, namely:

- the thickness of a filament must not exceed 6 pixels,
- a filament consists of at least 4 aligned pixels,
- there should not be any object within a certain distance to a filament (scale-dependent)

This last criterion is relative to the context. It reduces the number of unreal filaments that could be identified in case of insufficient masking in the binary image generation. For example, we detected that the cloud mask is not fully efficient, despite our efforts, and occasionally does not filter edges of thin clouds. These edges can yield false detections spectrally, with *false* pixels following a filament-like structure. Conversely, we wanted to be able to detect LWs located in noisy areas, so a trade-off had to be performed between the two needs.

Methodology

The identification module was organized in three complementary routines. The first one was based on the principle that filaments should be isolated from other objects. However, its sensitivity to noise made it insufficient in practice when there were objects nearby, or when noise surrounded a filament. Thus, the following two routines completed the filtering by dealing with the case of not isolated filaments (contextual filtering). For this, they reused the principle of routine 1 by adapting certain parameters. More precisely, routine 2 makes it possible to denoise the image and to remove certain larger objects, in order to improve the visibility of certain filaments. As for routine 3, its goal was to identify only certain long filaments, which would be obvious to an operator.

Routine 1: General case - Identification of isolated filaments

To deal with isolated filaments, the best option was to identify filaments at different scales. This method was based on the assumption that we are able to identify continuous filaments with a thickness of a single pixel. Then this same process allows the identification of thicker and discontinuous filaments at larger scales. The filaments were searched using 34 scales in total.

At Scale 1, the aim was to locate single-pixel thick continuous filaments, having an extent of at least 4 pixels and which are not too close to other objects or other parallel filaments. The process consists of successively eliminating all the *true* pixels from the image that do not belong to filamentous portions. At the end of this process, the bounding boxes of the remaining pixel groupings are the bounding boxes of the filaments identified. The search for the filaments was carried out in 4 stages:

1. Removal of thick clusters of interconnected pixels

A filament is a pixel grouping having a thickness equal to 1 pixel (or 2 pixels in some cases). To do this, we analyse the adjacency of *true* pixels:

- Any *true* pixel having more than 4 adjacent *true* pixels is deleted.
- Any *true* pixel that was adjacent to a deleted *true* pixel is also deleted.

2. Removal of thick clusters of interconnected pixels

To limit the complexity of the shape of a filament, *true* pixels adjacent to a *false* pixel were removed if they have a semi-cyclic shape. In other words, a *false* pixel must not have more than 5 adjacent *true* pixels. Likewise, a *false* pixel having 4 or 5 adjacent *true* implies that these *true* adjacent pixels should be removed, with the exception of the case of *true* pixels composing a continuous corner.

3. Criterion of distances between objects: Minimum distance

To minimize false positives, we ruled out parallel filaments too close to each other, which may appear usually under wave glint conditions. We also ruled out filaments that are too close to other clusters of pixels already identified.

To do this, we defined a minimum distance between the objects, and we deleted the *true* pixels that are too close. Thus, for each grouping of pixels, we checked around each of its *true* pixels that there is no *true* pixel of another grouping of pixels within a radius equal to the desired distance. If such *true* pixels are detected, the pixels close to these groupings were discarded (the groupings are therefore not necessarily deleted but cropped).

4. Removal of groups that are too small: Minimum width

To minimize spurious filaments of small size that could easily merge randomly within a noisy binary mask, we decided that a filament should be composed of at least 4 *true* aligned pixels. When the context is ambiguous, this limit may be increased. Let N be the number of pixels corresponding to the minimum width. Each bounding box of a pixel grouping must have dimensions greater than NxN pixels. Groupings that do not meet this criterion are deleted. Therefore, let W be the width and H the height of a bounding box. If $W \leq N$ and $H \leq N$, then this bounding box was discarded.

To identify filaments at an N scale, the original image is split into macro-pixels where each macro-pixel has NxN pixel size. For example, scale 2 means that each macro-pixel has a size of 2x2 pixels, scale 3 means that each macro-pixel has a size of 3x3 pixels, and so on.

We adopted the criterion that a macro-pixel is then considered to be *true* if it contains at least one *true* pixel. A macro-pixel is *false* if it contains no *true* pixels. The downside to this criterion is that the identification becomes highly sensitive to noise. On the other hand, it was necessary to identify very discontinuous filaments, which may be composed of spaced isolated pixels.

This process produces a new image of reduced size, composed by these macro-pixels. The previous identification principle was applied to this new image (Identification in Scale 1). The process repeats, producing a smaller image at each loop, faster at each time due to the reduction in the number of pixels. From scale 5 onwards, the computation time was practically constant and equal to the macro-pixel transformation time. This change of scale made it possible, first, to detect thicker continuous filaments and, second, to detect discontinuous filaments, as indeed, when the scale is adapted, the discontinuous filaments become continuous.

This processing had also a side effect on the detected shapes, because the larger the scale, the more precision is lost about the exact location of the original pixels and their alignment. This is why the elimination of the cyclic shapes is important, *i.e.*, it prevents weakly noisy areas from being identified as filaments.

Additionally, the maximum thickness of a filament can be controlled by adjusting the maximum number of *true* pixels associated with each macro-pixel. Thus, all macro-pixels with too many *true* pixels were removed. More exactly, if we denote the maximum desired thickness by *T* and if we consider a macro-pixel of size *NxN* pixels, then the macro-pixel is slightly enlarged in each vertical and horizontal direction so that it has a size of (N + 2xT) x (N + 2xT) pixels. If the number of *true* pixels associated with a macro-pixel is greater than (N + 2xT) x T, then the macro-pixel is deleted. In our case, we considered T = 6 pixels.

Finally, the minimum distance between objects and the minimum width of a filament depends on the used scale. The values used in the parameterisation are provided in Table S19.

The filament identification is performed on each scale of interest (in our case from 1 to 34). The next step was to bring the detections back to the scale 1 coordinate system and merge results. Let X1, Y1, X2, Y2 be the coordinates of a bounding box at scale *N*. The coordinates of this box at scale 1 would be:

$$X1' = X1 \times N \qquad Y1' = Y1 \times N$$

$$X2' = N \times (X2 + 1) \quad Y2' = N \times (Y2 + 1)$$

Then, we simply merged all the bounding boxes that intersect or include each other. For example, if two boxes intersect, their merging produces a single box, larger in size, *i.e.*, this is the box that encloses them. Once the results were merged, it was observed that filaments identified at a low scale can be relatively close to very large objects. Such objects can be poorly masked clouds or badly masked land parts, which would correspond to unwanted filamentous shapes. To remove most of these "unreal" filaments, a notion of relative size was added. It means that filaments near to a much larger object would be eliminated. To do this, each bounding box was enlarged by 60x60 pixels. If by enlarging the box the number of pixels has more than doubled, then the detection is removed. It was verified that this method allowed us to pinpoint continuous and discontinuous filaments of complex shapes.

Routine 2: Improving the visibility of certain filaments, denoising, then identify again

The method described in Routine 1 was effective in identifying various shapes of filament, both continuous and discontinuous. However, the same operation in macro pixels was sensitive to noise and it only worked for isolated filaments. For the case of pixels very close to a filament (with a distance lower than the defined minimum distance), we made the following assumptions:

- these pixels were considered as part of the same object. The filament may then be too thick, or an invalid adjacency may appear, not being considered valid;
- the portions of the filament which were close to these pixels were cropped. If the remaining portions are then not long enough, then the filament was also considered invalid.

To overcome some of these unwanted effects, this routine re-calculates detection independently of Routine 1. Therefore, we combine two independent sub-approaches of denoise to improve the visibility of some of these specific filaments. The image was therefore modified to redetect the filaments, using the same principle as in Routine 1 but modifying certain parameters (scale, minimum distance, and minimum width).

Subroutine 1: Removal of small isolated groupings of pixels

As we mentioned, a filament may not be identified properly if there are small groups of pixels nearby. When switching to macro-pixels, cyclic structures may appear, or the adjacency of *true* pixels may be greater than 4. To circumvent this problem, we locate these areas by removing isolated groupings of pixels and then by looking for the filaments again with the previous method on the modified image.

1. Removal of isolated groupings

The removal of isolated pixel groupings could not be performed in any area of the image. If a large area contains noise due to glint, removing isolated groupings could create false discontinuous filaments. In fact, there may be a big spacing between the groupings of pixels in these areas, but this spacing was not uniform throughout the noise zone. Thus, removal took place when it was in areas with few pixel groupings. The process was as follows:

- We started by looking for groupings of pixels with a size lower than 10x10 pixels. For each of these pixel groupings, we checked around them, within a radius of 12 pixels, that there were no other pixels except those of the considered grouping. If this condition was *true*, the pixel grouping was removed from the image.
- It was then verified that the suppression had not taken place in noisy areas with large spacing. To do this, where a grouping of pixels was previously deleted, it was checked within a radius of 30 pixels that only this grouping of pixels was deleted. If not, the deleted pixel grouping was reintegrated into the image.

2. Filament identification

Once the image was free of small isolated grouping of pixels, filament identification routine was applied again, but only from scale 5 to 34 and by replacing values to a minimum distance of 4 and a maximum width of 5.

Subroutine 2: Removal of very small groupings of pixels in noisy areas

Following the same principle as during the previous removal of small isolated groupings of pixels, this process aimed to denoise the image in order to identify slightly thick filaments in noisy areas. This process was as follows:

- We started by looking for large areas of noise (like those affected by glint). For this, the image was split in macro-pixels of size 200x200 pixels. If a macro-pixel had at least 7 groupings of pixels of size lower or equal to 2x2 pixels, it was considered to probably contain noise. If a macro-pixel had at least 7 adjacent macro-pixels which probably contain noise, then that macro-pixel had noise.
- In the previously identified noise zones, the small groupings of pixels with size lower or equal to 4x4 pixels were removed. Small isolated groupings of pixels smaller than 10x10 pixels and not having another nearby pixel within a 6-pixel radius were also removed.
- Outside these noise zones, small groupings of pixels having a size lower or equal to 2x2 pixels were removed.

Then, we applied again the removal of isolated groupings and the filament identification. This time, as the denoising was stronger, we looked for the longest filaments to avoid false detections with the parameters given in Table S20.

Routine 3: Special cases - Identification of "obvious" filaments having objects nearby

The Routine 2 denoised the image to improve the visibility of slightly thick filaments. However, denoising also generally eliminates discontinuous filaments. Likewise, if objects larger than 4x4 pixels are distributed around a filament, the filament could not be identified. The Routine 3 was not intended to identify all potentially still missed filaments, but to identify specific "obvious" filaments, as the remaining cases were quite rare or too difficult to detect without high risk of false identifications. A filament was considered "obvious" if it was long and semi-rectilinear or if it was long and almost continuous.

This Routine 3 was carried out in two subroutines. Firstly, we looked for the filaments which are long, semi-rectilinear and not very thick in areas with little noise. Secondly, we looked for long and almost continuous filaments, regardless of whether the zone was noisy or not.

Subroutine 1: Detection of long semi-rectilinear filaments

We started by identifying mid-long objects according to the principles of Routine 1 and using the parameters in Table S21. By deactivating the distance criterion, we identified potentially missed filaments in low noise areas. Unfortunately, this process also selected other objects that are not filaments. Indeed, the change of scale allowed linking together the groupings of pixels close to a distance of N pixels (if we are at the N scale), however, there are configurations where this connection can create false filaments (this phenomenon occurs most frequently in noisy zones having a shape of clouds of points). Therefore, two complementary schemes had to be applied to differentiate the “obvious” mid-fine long filaments from other objects.

Approach 1: Search for long semi-rectilinear filaments with a thickness lower or equal to 3 pixels

The first approach identified very fine, semi-rectilinear long filaments. It used Principal Component Analysis (PCA) in the case where the *true* pixels of an image are represented in a plane, and they are considered as a 2-dimensional random variable. In this way, the PCA will determine the 2 axes that best explain the dispersion of the object to be analysed, interpreted as a cloud of points. It will also order them by explained inertia, the second axis being perpendicular to the first.

In filaments, the first PCA axis is much larger than the second (perpendicular axis) when the pixels have a semi-rectilinear shape. In addition, the intersection point of the two axes corresponds to the barycentre of the *true* pixels.

Thus, we have a rectilinear filament when the ratio of the smallest axis by the longest axis is close to 0. However, this method only works if the pixels compose a point cloud. There must be enough points (at least 4) and there must not be a compact set.

After application of the multi-scale identification, we obtained the bounding boxes of the selected objects. Those were used later to crop the images and obtain snippets, which were later on used during the post-processing.

To apply the PCA, we have first to be assured that most of the pixels of the studied thumbnail do not constitute compact sets. Most of the pixel groupings of the considered thumbnail must not be too thick for the method using PCA to work. This way, 98% of the *true* pixels in a thumbnail should not belong to areas that are too thick. In our case, the maximum thickness did not exceed 3 pixels. In addition, the ratios used with PCA differed according to the maximum thickness.

To determine if the considered thumbnail contains a rectilinear filament, a PCA was applied to the sub-thumbnail located in the centre of the thumbnail. This sub-thumbnail has dimensions 100x100 pixels. We also verified that this sub-image had at least 4 *true* pixels before applying PCA. If this was the case, we calculated the ratio and applied the following criteria:

Case 1 - Maximum thickness less than or equal to 2 pixels

- If the ratio ≤ 0.2, the thumbnail contains a semi-rectilinear filament.
- If the ratio ≤ 0.3, we must use a larger sub-thumbnail, of size 175 x 175 pixels and recalculate the ratio. If the new ratio is still less than 0.3, then the thumbnail contains a semi-rectilinear filament.

Case 2 - Maximum thickness between 2 and 3 pixels

- If the ratio ≤ 0.15, the thumbnail contains a semi-rectilinear filament.

Approach 2: Search for long semi-rectilinear filaments with a thickness between 2 and 3 pixels

The Approach 2 complemented the Approach 1 by identifying other "obvious" semi-rectilinear long filaments with a thickness of between 2 and 3 pixels. Approach 2 analysed the thumbnails that the first solution considers as not containing any filament (which is not necessarily the case). This method was similar to Approach 1 and also involved PCA. Most pixel groupings in a thumbnail should be between 2 and 3 pixels thick.

The true pixels of a filament are a small area of a given thumbnail. Therefore, the thumbnail was first enlarged, *i.e.* by taking w as the width of the thumbnail and h the height of the original thumbnail, the new size of the thumbnail becomes (w + 3 × 25, h + 3 × 25) = (w + 75, h + 75). The thumbnail was then split into macro-pixels where each macro-pixel has a size of 25×25 pixels. A macro-pixel was considered *true* if it contained at least one *true* pixel. Thus, if we have:

$$\frac{number\ of\ true\ macropixels}{total\ number\ of\ true} < 0.5$$

where the total number of macro-pixels is equal to (w+75) / 25 × (h+75) / 25, then the thumbnail potentially contains a filament. To be sure, we limited results to semi-rectilinear cases using the ratio calculation using PCA over the entire thumbnail, *i.e.* if ratio ≤ 0.15, then the thumbnail contains a semi-rectilinear filament.

Subroutine 2: Detection of quasi-contiguous long filaments

Case 1: very thin filaments

Once the search for long mind-fine filaments was performed, a second step required the application of a search for long quasi-continuous filaments. To do so, we repeat the same process as in Routine 1. However, the smaller the scale, the less the identification is sensitive to noise and the less there were false identifications. Thus, it was feasible to identify fine (less than 4 pixels), quasi-continuous filaments longer than 15 pixels with the adjusted parameters given in Table S22.

Case 2: thicker filaments if presence of big sets of pixels

The scales 1 to 3 applied above were not able to identify quasi-continuous filaments thicker than 3 pixels. To identify thicker filaments, it was necessary to use larger scales. However, these scales were sensitive to noise. The solution to

draw inspiration from the denoising in Routine 2, with the difference that we removed all the large objects in order to keep only long, continuous portions of filament. In this way it was possible to identify the thick, quasi-continuous filaments even if they are surrounded by large objects, which Routine 2 was not able to do. As this process can be computationally expensive, four iterations are performed and results are then merged. An iteration consists of:

- removing all objects with a size lower or equal to a certain value.
- identifying the filaments on the image so denoised.

The four iterations removed respectively filaments larger than 13x13, 17x17, 21x21 and 25x25 pixels, respectively. At each iteration, filaments were then detected as in Routine 1, but only from scale 6 to 13, and with a minimum distance of 0 and a minimum width of 10 for each scale.

Limits

The described method identified and individualized most of the filaments in an image. However, in the case of non-isolated filaments, there were some limit cases where filaments were not detected. This especially happened when noise, nearby objects or portions of a filament had a size not supported by Routines 2 and 3. In summary:

- the method may not identify discontinuous filaments that are very close to each other;
- discontinuous filaments with a large spacing between each portion can be detected poorly or not at all if they had objects nearby;
- a discontinuous filament constituted of very small portions and having large objects nearby can be undetected;
- a discontinuous filament constituted of very small portions and which is located in a noisy area can be undetected, especially if its portions have the same size as the noise.

It was challenging to identify the previous cases because binary images can also contain filaments that are not LWs. Normally, the binary image should not contain or should contain few such filaments. However, the method we use aims to be robust in preventing these false identifications. Below, we present three cases where the masking algorithms performed particularly poorly, in order to anticipate potentially problematic contexts.

Clouds

In the S2-MSI spectral data, clouds share some properties with ML. Their spectral features can be similar. To avoid identifying clouds as windrows, they were masked out. However, the edges of clouds were in some cases very difficult to mask out, which generates false filaments. In this regard, it is essential to use a notion of distance in Routine 1. Similarly, denoising cannot be carried out on objects of any size, as otherwise it can yield false identifications. The method was robust against poorly masked clouds when the false filaments were in areas with a high pixel density. It was unfortunately common to find cloud edge filaments in areas with low pixel density. Under these conditions, the filament identification method was unable to determine whether they really were filaments. To avoid these false windrow identifications, the masking algorithms must therefore be as efficient as possible.

Land

In some areas, land can also be a source of false identifications. If the land is masked incorrectly, false filaments can appear. As happened for the clouds, the masking algorithm must be very efficient to prevent this kind of false identification. In our PoC, land masks obtained via radar images, such as SRTM data were particularly efficient.

Noise

It was difficult to eliminate noise without degrading the filaments on the image. Although it was rare, noise can generate false filaments. This was particularly true near the coast, due to the stronger wavy pattern of the surface. Denoising must be applied with caution in such cases.

Performance

The method used for automatically identifying filaments has the advantage of being parallelizable because we could treat the different scales independently. The total execution time of the contextual classifier on a 4-core computer was:

- About 1.5 minutes if there was relatively little noise in the binary mask.
- About 4 minutes if the noise level was high (denoising is a very demanding step).

Routine 1 had the advantage of being easily modifiable to identify isolated filaments according to various criteria. Indeed, it allowed us to simply control the distance between objects, the minimum extent, the desired thickness, etc. Moreover, in the case where we only searched for perfectly isolated filaments, the runtime for the 34 scales on a 4-core machine was only 20 s.

Routines 2 and 3 allowed for detecting "obvious" filaments in specific context cases. However, there are still cases where the method does not detect filaments. These were cases where the filament was very discontinuous, with spaced points, noise or objects similar in size to the portions of the filament. These cases were nevertheless rare.

Therefore, the performance of the filament identification module depended significantly on the quality of the binary image, *i.e.* the more the filaments were isolated, the better false identifications were avoided and the more visible and detectable the filaments were. In practice, the filtering methods used to identify filaments meant that aggregations with a total length of less than 70 m were discarded.

Large part of the difficulties with the spectral classification come from the lack of adequate spectral information in the S2-MSI. Eventually, with dedicated spectral bands (Table S23), it will be possible to disentangle the contributions of the main litter components to the remotely-sensed reflectance. An optimal sensor would allow a loosening of the contextual filter here applied because of the noise in S2-MSI images. A proper account of small litter patches should result in a significant increase in the number of total detections.

S2.2. Testing of the PoC processor

To check the operation of the proposed retrieval chain, we performed some essays over six test cases in order to:

- estimate the preliminary accuracy of the spectral classifier and of the automatic windrow identification module;
- identify any potential fine-tuning of thresholds and/or processing steps that could be required for a more general solution;
- detect any potential limitation/problem that should be addressed before launching the mass processing;
- start estimating the potential scientific added-value of the PoC processor for research and management.

For each of these test areas, the full time series of S2-MSI L1c images were collected at the time of the exercise and processed with the PoC processor for their individual assessment. Note that the validation process was iterated several times in order to fine tune parameters of the processor before the final full processing.

It is worth mentioning that some of the detected LWs were located in areas where Ocean Colour could indicate the occurrence of a front, while others were not, implying a different originating mechanism.

S2.2.1. Known issues

On the downside, there were a number of situations where the processor did not perform perfectly, which are described below.

Wave glint

The issue of the so-called wave glint was present and led to false positives in some areas. This was mitigated by the denoising of the binary mask introduced previously and also by including a search radius around noisy areas so as to avoid identification of fake windrows in those. However, this is still found sub-optimal and requires improvement.

One of the proposed solutions could be to look at the spectral property of wave glint pixels and try to identify specific features that could discriminate between those and true detections. However, this method has not been yet tested and it is a concept to further explore.

Residual clouds

Even if the cloud masking introduced in the data processor could be considered part of the state-of-the-art of cloud detection over water masses, it was not perfect. Small thin clouds were not yet fully identified and they induced some false positives. This problem was less worrisome than the case of the wave glint, but the shape of those cloud edges

mimics windrows so they can raise false identifications. It is one of the factors limiting the lower length of filaments that the PoC processor was able to report, and should be specifically addressed in the future.

As a mitigating strategy, the ML mask was further filtered by checking the presence of bad pixels in a given distance from the pixel. In particular, a region of 100 m around each positive pixel was checked, and if a given bad pixel was found, the detected pixel was rejected. This method significantly reduced the issue of undetected clouds, and also compensated for some residual errors in land mask and footprint detector masks. However, it has the drawback of occasionally removing valid *true* pixels.

Misaligned detector footprint

We found, at the time of the PoC running on the datasets, that the polygon given in the Sentinel-2 products to detect the footprint of the detectors was not always accurate enough. They are obtained by purely geometrical computation, which is not always aligned with their impact in the reflectance values at surface level.

This issue was mitigated by considering a radius along the detector footprint so as to mask data there, again at the cost of removing some potentially valid pixels.

Limitations of the deterministic object classifier

Whereas the current performance of this functionality was good enough for the purposes of the PoC, there is clearly room for improvement. The classifier is able to find most of the true windrows but still misses some, and occasionally fractions some of them in separated windrows, whenever the filament contains some large gaps in the ML mask generated with WSI. This could be improved in future versions, by exploiting the results of the mass processing to generate a label dataset that can be employed by Machine Learning algorithms, which may help to obtain a seamless window identification of better characteristics than the current one.

S2.2.2. Automated pre-screening functionalities

As a result of the above issues, a number of fake windrows could be detected. To support the validation of the data processor and the manual screening of outputs, a series of filters were implemented on the PoC processor to identify these unwanted filaments. This post-processing was based on the manual assessment of the results generated by the consecutive runs over the verification sites. The manual assessment provided the most common cases of false positives, which were used to design automated discrimination methods.

The following paragraphs describe the automated filters that are applied within the PoC processor. For a filament being considered as *true* by the PoC processor, all filters must be passed. Otherwise, it will be flagged as *false*.

Tile-edge effect false positives

In some cases, we observed that the upscaling of all S2-MSI bands to 10m spatial resolution caused undesired artefacts at the edges of partial images (*i.e.* the satellite provides images that are cropped to specific tiles). When the orbit intersecting a tile does not overlap it entirely, there are null sections in the image. The boundary between the null data and the valid data suffers from this issue. To discard fake filaments originated by this effect, we simply created a Boolean mask with the criteria valid/non-valid pixel, using the default values detailed by ESA in the data product description. Once the mask was generated, we performed a dilation of the mask 8 times. A dilation consisted of propagating the false values to the neighbour pixels. Repeating this 8 times, we removed an edge around these damaged borders. We then measured the fraction of valid pixels in the filament before and after the dilation process. If the number of valid pixels was still higher than 35% of the original value, we retained the filament, otherwise it was flagged as a *false positive*.

Wave glint false positives

As indicated, S2-MSI images suffer from wave speckling, which depends on sea state and the geometry of the observations with respect to the sun. This induces a line of glint that appears on the WSI as positive pixels. As these waves are linear, the contextual classifier takes them as filaments. Fortunately, this kind of false detections is composed usually of multiple fake filaments in the same snippet, close to each other. Therefore, performing some analysis on the snippet associated with the filament is possible to detect and flag them. To do this, we used properties of images based on information theory. As these *false windrows* are structured information, it is possible to compare the level of information they introduce in the snippet vs a random noise structure. Thus, we computed the Shannon's

entropy of the snippet over a Normalized Difference Water Index (NDWI) equivalent of the multispectral image H_{NDWI}:

$$\text{NDWI} = \frac{(B3 - B8)}{(B3 + B8)} \qquad H_{NDWI} = -\sum_{x \in NDWI}^{n} p(x) \log p(x)$$

H_{NDWI} is calculated by convolution of the image using the Shannon's entropy definition as a filter of a certain radius applied to each pixel of the image. In our case, we chose a radius of 10 pixels, using a circular shape as template (*i.e.* no pixel included in the convolution will be to a distance $d > 10$). Shannon's entropy H relates to the probability function of a given value within the value distribution of the dataset, or in our case, the resulting NDWI image. We then compared its value in the image with its standard deviation, so to compare the mean value of information in the resulting H_{NDWI} image with its dispersion:

$$H_{NDWI}^{norm} = \frac{H_{NDWI}}{\sigma(H_{NDWI})}$$

A single filament vs a background yields a much lower level of information than the case of multiple filaments in one single snippet. Consequently, the mean value will be generally higher in the case of false positives caused by wave glint than in real ones. On the other hand, the dispersion of the local information measures the heterogeneity of the general background of the image, which tends to increase the overall information level. Thus, H_{NDWI}^{norm} is de facto a texture indicator. Setting a threshold, it was possible to separate valid filaments from detections associated with wave glint. The threshold was set from the H_{NDWI}^{norm} values for snippets affected by wave glint that we manually identified during the test runs of the PoC processor. Most of them were associated to $H_{NDWI}^{norm} > 22$, hence using this threshold to identify false positives by wave glint.

Cloud edge false positives

Other of the origins for false detections we found in the assessment were the edges of thin clouds, which are also confused by WSI as potential ML pixels. As these false positives tend to appear in the neighbourhood of clouds, we also introduced a filtering strategy similar to what we did for the tile-edge effect. We grabbed the cloud mask we produced in the normal flow of the PoC processor, and selected the area matching the snippet. Then, we performed a dilation of the mask 30 times, and applied the mask to the snippet. If the valid fraction of the pixels in the filament after applying the new mask was below 5% of the original number, we flagged the filament as false.

However, this method was not always able to detect this issue, so additionally, and similarly to what we did for the wave glint cases, we found out that the structure of the information within the snippets affected by this could be used to separate them from the valid cases. In this case, the problem was the opposite, *i.e.* the detections done in cloud edges tended to be very wavy and much less uniform than the case of wave glint filaments. As a consequence, the values of H_{NDWI}^{norm} were much lower. Following the same procedure, we inspected the values for a set of snippets affected by the edge of thin clouds, and identified them during the manual inspection. We found that snippets with $H_{NDWI}^{norm} < 5$ were mainly associated with cloud edges, using this threshold in the post-processor to flag them.

Foam and boat track false positives

We also identified cases where foam present in trailing waves generated by ships led to false positives. Those were particularly difficult to separate from real filaments by other means, so we applied a two-step approach to identify them.

In the first step, we computed the mean values of three indices, namely NDVI, NDWI and DBI, for each of the snippets containing filaments. The inspection of the cases found during the manual assessment of the test sites yielded that a filament was likely false if $\underline{\text{NDVI}} < -0.3$, $\underline{\text{NDWI}} > 0.5$ and $\underline{\text{DBI}} \geq 0.15$. We used such values to flag false positives caused by this reason.

In the second step, we verified that the standard deviation of the B02 and B08 for the pixels identified by WSI in the filament could be used to further identify the issue of boat tracks. Whenever $\sigma_{B02} > \sigma_{B08}$, the filament is likely associated with a boat track. This is explained because the boat track induces an unnatural variability in B02 that is not matched in the B08 because of water absorption. Should the filament be true (*i.e.* containing actual floating matter), variability in B02 would follow variability in B08.

S2.2.3. Verification sites

Three batches over six selected test sites were chosen for the verification, as these were known Sentinel-2 tiles where LWs had been previously identified. The sixth one is different, as corresponds to field experiments with artificial targets[5,6], which has been used often for development and validation of EO4ML techniques.

- Po River: LWs were identified in the mouth of the Po River in Northern Italy as part of these other efforts, including in-situ observations. Additionally, during the period of the validation of this region a transect of the area observed by Sentinel-2 was surveyed by a research vessel. The researchers aboard were able to visually verify the presence of LWs in the area, later identified by the PoC processor. The majority of the detections in this tile were made in the southern part of the AOI, which is consistent with the circular ocean currents in the area.
- Calabria: As with the Po River location, there were previously several LW detections off the coast of Southern Italy, close to the region of Calabria, making it a good candidate for further filament detections. There are multiple freshwater inputs to the ocean in this area, resulting in a large number of detected windrows.
- Crete: Another area with previously identified LWs, the Greek island of Crete also has ocean dynamics which favour the aggregation of floating ML. Close to the city of Retino, multiple windrows were detected.
- Granada: The waters off the coast of Southern Spain, close to the city of Granada and Punta del Cerron, were another known location for LWs from a field campaign carried out by the University of Cadiz. Several longer filaments were detected during the validation of products over this region, where the processor identified them as multiple individual filaments due to the fragmentation of the filament over its length.
- Rome area: Multiple freshwater outlets close to the city of Rome led to an increased frequency of LW identification within this region.
- Lesbos Island: The processor was tested on tiles containing artificial plastic targets deployed as part of the Plastic Litter Project (PLP) 2018, 2019 and 2021 campaigns performed by the University of the Aegean[5,6,56]. The targets in PLP 2018 were composed of plastic bottles connected with thin twine, plastic bags, and thin fishing net. For the PLP 2021 campaign, the targets were composed of plastic bottles held in place with a mesh barrier, and a large High-density Polyethylene (HDPE) sheet. In both campaigns, this allowed for a target of sufficient size to be visible in the S2-MSI.

The processor was able to identify the plastic pixels on the campaign targets in both dates. In the PLP 2021 campaign image, there were additional pixels identified in the product by the PoC processor, but these would only be flagged as a LW in the full processing run if the pixels were arranged in the form determined as a filament.

S2.2.4. Initial supervision test of the PoC processor

After the setup of the PoC detection processor was completed, the processor was tested on the test batches to determine the accuracy to which it was able to classify LWs. After the results of each batch were analysed, adjustments were made to the processor algorithm to improve its accuracy at classifying *true* LWs and rejecting false positives. The processor was then run on the next batch to test the improvements.

The processor outputs from each test were classified based on their accuracy. Snippets of each candidate filament were split into categories of *true* LWs and *false* positives, based on the human supervision by a team of 6 operators. Processor performance was then judged on whether the processor was able to accurately determine true snippets from false positives. This was used to generate statistical data on the accuracy of the processor upon processing each batch.

The first batch was run on 1,633 products from 2017 run over the 5 sites under consideration (fake-target experiments of Lesbos Island were excluded from these routine tests). These results are provided in Tables S24 and S27. The Receiver Operating Characteristics (ROC) statistics showed that this first batch had poor results for the automatic screener. Only 50.92% of *false* positives were identified as such. The identification rate for *true* windrows was 87.77%, meaning that in the instances where filaments were present, the chance of the processor identifying them was high. After improving the capability of the processor to identify *false positive*, the processor was run on a second test batch, consisting of 2,575 products across the same 5 test sites in the year 2018. After validation, the results of these consecutive tests are given in Tables S25 and S27, respectively.

After improving the post-processing screening methodology, the processor was much more accurate in identifying *false* positives, with an 81.47% accuracy value (increasing 30.55%). The accuracy of identifying *true* windrows was slightly reduced to 83.05%, but this trade-off for the improved accuracy in identifying *false* positives was deemed acceptable at this stage.

The next round of improvements focussed on further increasing the accuracy of *true* windrows identification while maintaining the high accuracy of the screening methodology for filtering out *false* positives. The final verification batch consisted of the same products used for the second verification test. This allowed for a more direct comparison of the effects the processor methodology changes were having on the processor performance. The statistics derived from the third verification batch are given in Tables S26 and S27.

The third and final verification test resulted in an improvement for both the sensitivity and selectivity of the processor compared to the second test, with a true filament identification rate of 86.47% and a correct *false* positive screening rate of 87.81%. These results were considered as acceptable for the full exercise and adjustments in the algorithm were frozen for the full run.

S2.2.5. Validation

There is a significant lack of quantitative field data to validate satellite observations of ML, both in terms of composition and coverage. Given this data scarcity, the consistency of the satellite observations in this work was largely analysed on the basis of the spatial and temporal relationships of LW detections and environmental drivers (wind, rainfall, runoff, population, MPW) (see "Spatio-termporal drivers" in Methods). Nevertheless, apart from this analysis, an effort was made to test the robustness of the satellite observations directly with ML data. Available field data come mainly from two sources, firstly, marine debris accumulations tagged on S2-MSI images (MARIDA dataset[7]), and secondly, experiments deploying artificial ML targets at sea[5,6,56]. While artificial targets are compatible with S2-MSI acquisitions and include on-site quantitative information on ML composition and density, the open-access MARIDA dataset was manually curated and lacks such information. Both datasets were analysed in the present work (Figs. S9 and S11).

Because we did use MARIDA dataset to determine different spectral classes and constrain the definition of WSI, it would be inappropriate to use the same data for validation. Instead, we used the fake-target experiments[5,6,56] to test the detection performance of the PoC spectral index (WSI) (*e.g.,* Fig. S11).

S2.3. Data processing

Once implemented and tested, the PoC processor was used for the mass processing over the ROI, in order to obtain broad-scale maps and trends to assess their consistency and usefulness for scientific and management purposes. A total of 288,166 S2-MSI images (150 TB of data) corresponding to 411 tiles over the Mediterranean Sea were processed (Fig. S12), covering the period from 4 July 2015 to 21 September 2021 (75 months).

S2.3.1. Data acquisition

Sentinel-2 products to be used as input data for the processor were sourced from the Luxembourg Space Agency (LSA) Data Centre. This organization archives all Sentinel-2 products which are made available and enables access to them through a virtual machine terminal to their system.

All water-containing tiles of the Mediterranean Sea were requested for the PoC processor to run on the lifetime of Sentinel-2 A and B at the time of the study. These were provided across 7 batches due to the memory taken up by such a large number of products. While there were regions where tile coverage overlaps, the output filename nomenclature was written to account for this and allow for easy identification of duplicate filaments.

S2.3.2. PoC processor outputs

Candidate LWs detected by the processor within a Sentinel-2 tile were automatically catalogued and the results were saved into an output file. The output contained the masks and snippets folders, as well as a netCDF product detailing the metadata of all the recorded filaments. The full list of recorded variables is given in Table S28. This file can be accessed using an appropriate netCDF reader or script, targeting specific properties or extracting all of them into an alternative format. The mask folder contains *.png* products with black and white representation of the pixels identified as containing ML by the processor. The image with the suffix *bm_1* contains only the pixel positions relative to the full tile. The *bm_2* product additionally contains the squares overlaid on the arrangements of identified pixels the processor has identified as filaments. The snippets folder contains *.png* images providing quick-looks of the identified filaments in the Sentinel-2 product. These were sorted by the processor's built-in classifier as Good, Bad, and Suspect. Good filaments are those the processor classified as *true* positives, Bad filaments are those the processor classed as *false* positives, and Suspects are those the processor was unable to classify. The final folder in the snippets directory

is the Spectrum folder, which contains the average spectrum for each pixel within each snippet in the folder. This spectrum is across the wavelength bands present in the Sentinel-2 MSI, with the averaged spectrum present at the top, and the standard deviation of the spectrum in each band.

S2.3.3. Supervision of the PoC processor outputs

The final assessment of PoC processor outputs was performed once the processor ran on all tiles, which yielded 104,286 output netCDF files for a total of 708,742 filaments. The validation was performed by the supervising team of six researchers on each individual candidate filament, sorting them into *true* or *false* positive categories based on the set of guidelines provided to them. It should be noted that the automatic graphical output of the PoC provides snippets including four images for each detected filament. The typology of these images was chosen to be as useful as possible for detecting artefacts in the detections. In particular, the image clipping for each filament automatically detected by the PoC processor included:

- the TCI image of the reported filament;
- the same as above but with a mask of red pixels applied to the pixels reported as ML using WSI index;
- the NDWI image for the same bounding box;
- the WSI_C component of the WSI index, as reference.

The total of 708,742 candidate filaments detected by the PoC processor in the Mediterranean Sea was supervised. The output of the processor was organized according to the S2-MSI tile (of the 411 that were processed) with which they were associated. Each operator was assigned a list of tiles (both for the test runs and full run) to label each detected filament as a *true* or *false positive*, having to reorganize the filaments in separate folders (True and False).

To determine whether a candidate filament was true or false, operators used a catalogue of fake filaments that were identified by a pre-screening of the results and curated by their spectral signature and sources. Six different contributors to false positives were recognized in the supervision, *i.e.* aeroplane exhausts, cloud edges, wave glint, ship trails, human-made structures and image stitching (Figs. S34 to S39). The corresponding clippings were archived in an open repository to support the development of a fully automated classification (www.oceanscan.org). A first version of an AI-based classifier trained with the manually labelled filaments has already been completed, with an accuracy of 97% classifying true filaments, and 96% classifying false positives.

To measure accuracy of the classification and detect potential biases, operators were provided with some repeated tiles, so we could compare the performance on the classification by inter-comparing separated classification over the repeated tiles. We found out that some operators were inclined towards rejection, whereas others towards acceptance. Taking a conservative approach, we revisited the tiles reviewed by the operators with a positive bias to perform a stricter selection and to minimize the inclusion of errors in the classification.

For the final run, all the filaments flagged as *true* positives were provided to an independent experienced operator who performed a second check. The supervision team classified all the 708,742 LW candidates, of which only 14,374 were found as *true*. This shows the harshness of the scrutiny and how selective we were in the filtering.

To test processor performance, we used double supervisions, performed by different operators, on data subsets of several validation sites. Statistical analysis of the snippet data from the full processing run over all Mediterranean tiles is provided in Tables S29 and S30. Differences in the statistics generated over the full dataset and those generated at the testing sites were mainly related to the precision of the processor. This was reduced to 13.66% from the 39.86% displayed in testing. One of the reasons could be the large increase in open-water tiles present in the full dataset compared to the validation dataset. Open-water tiles were not expected to contain as many identifiable filaments, so many of the filaments found by the processor in these areas were either false positives or true negatives, increasing the proportion of these counts. This explanation was further supported by the difference in the proportion of filament classifications between the validation and full processing run (Table S31). In this later case, we found a decrease in the proportion of True-Positives due to the introduction of more True-Negative detections associated to the additional open water tiles present in the dataset. The small change in the proportions of False-Positive and False-Negative identifications between the initial validation and full processor runs indicates that the limitation of the processor to correctly identify true filaments or correctly eliminate false filaments had a small effect on the larger dataset, with a less-than-1% change in the figures of the two runs.

Of the six sources of false positives in the filaments automatically identified, the most numerous across all tiles were cloud edge false positives. Surface glint was less common; however, it was detected in large areas of tiles where it was present, resulting in a large proportion of false positives in sunglint-affected tiles. Man-made structures were

detected more frequently on coastline-covering tiles, with the same structure being detected multiple times across several tiles.

The corresponding clippings of false positives and true filaments were archived in an open repository to support the development of a fully automated classification.

S2.4. Detection threshold for the PoC processor

To estimate the minimal coverage fraction that the current version of the processor is able to detect LWs on S2-MSI images, we made use of a LW confirmed in Bay of Omoa (Honduras) and included in MARIDA database[7]. The method applied followed the procedure described in section S2.1.7, briefly:

- WSI was computed using its Boolean form to identify the pixels that contained litter within the image.
- Secondly, we computed WSI in its numerical form, and found the maximum and minimum values within the pixels composing the filament, as per the mask resulting from using WSI in its Boolean form. In other words, we retained the numerical WSI values of the pixels that comply with the criterion for the WSI mask, and focused on the minimum and maximum values.
- In a third step, we isolated the spectral profile of the LW pixel offering the highest value and took it as 100%-pixel coverage. This was visually confirmed, as this LW in Omoa Bay is large enough to contain 'pure' ML pixels. Then, we selected a nearby pixel but separated enough from the filament to be considered as 0%-pixel coverage, *i.e.,* a 'pure' water pixel, and isolated also the associated S2-MSI spectra.
- Using those two spectra as reference, we built a linear spectral calibration curve going from 0% to 100% at intervals of 0.1% by linear mixing of the two profiles (e.g., Fig. S9b).
- WSI in numerical form was calculated for all the resulting synthetic curves obtained in the previous steps (e.g., Fig. S9b.
- Lastly, this information was crossed with the lower WSI value found in step 2 to find the minimal coverage (in %) needed to trigger a detection in WSI in its Boolean form (Fig. S9c).

This method resulted in a minimum coverage above which the PoC processor detects ML presence of 21% (Fig. S9). Interestingly, this threshold coincides with the estimate derived from experiments with artificial plastic targets on Lesbos Island (20%[5]). The calibration curve could be used to map WSI in numerical values to fractions of the pixel covered by ML for this particular case (Fig. S9a).

SUPPLEMENTARY DISCUSSION

S3. Supplementary Discussion for Mission Conceptualization

S3.1. Experimental Plan

S3.1.1. Lab experiments

During the laboratory analysis, we generated files containing the spectral reflectance of the plastics as obtained by the ASD spectrometer for all the scenarios[3]. Since the ASD spectrometer measures reflectance, minimal pre-processing was required to get spectral signatures. The reflectance spectra database could be explored to investigate effect of multiple factors (*e.g.*, biofouling, plastic ageing, angle of observation)

Indeed, it has already been compared with existing datasets using similar techniques, materials and procedures[22]. In this way, confirmation of outcomes could be achieved and assurance of scientific quality of the measurements was provided. Systematic errors appearing in the comparison allowed for applying correction to the data to harmonize the results with existing libraries (*e.g.*, spectral libraries for polymers provided by the University of Connecticut).

Results of the experiments

Effect of sample colour on spectral reflectance

The bulk spectral reflectance properties of the samples exhibited variabilities related to the apparent colour of the objects over the wavelength range from UV to SWIR (Fig. 7 in Garaba et al., 2021[3]). The 100%-pixel coverage of HDPE pieces with different colours (orange, blue, white, purple, green, black) had spectral reflectance lower than 80%-pixel coverage HDPE of only white pieces. For the white pieces, a nearly flat but decreasing signal was observed in the visible spectrum, whilst in the multi-coloured measurement the bluish, greenish and reddish peaks resulting from different colours were salient. Disregarding the 100% coverage signal, a near linear trend at each wavelength correlating pixel coverage and spectral reflectance was found (Fig. 4 in Garaba et al., 2021[3]).

At the measured nadir angles, slight differences in the spectral reflectance magnitude were noted, suggesting that the reflectance of the samples at different wavelengths was heterogeneous, although the correlation between reflectance and pixel coverage was relatively consistent (Fig. S3). It is possible that the orientation of the samples with respect to the position of the sensor and light source influenced spectral reflectance measurements. The relationships between object size, orientation, and optical properties have been previously discussed but are not yet fully understood[3,60,61].

Dry and wet plastics

In general, the dry raw samples showed higher reflectance compared to the wet samples (Fig. 6 and Table 2 in Garaba et al., 2021[3]), which is consistent with prior studies[21,22]. The presence of a water layer on the samples resulted in a significant decrease in the reflectance of the object because water itself is a very strong light absorber.

PS had the highest reflectance likely because the samples were bright white targets, while LDPE showed the lowest reflectance largely due to being transparent targets (Fig. 7[3]). There was a general positive correlation between the measured spectral reflectance and the percentage pixel coverage by the plastics (Fig. 8 in Garaba et al., 2021[3]). At the lower pixel coverages, outliers suggested a possible limit in detection for the specific material.

All the wet samples floated except for the LDPE which was slightly submerged. This could explain why the reflectance was lower than the other samples over the whole wavelength range. Different objects made of HDPE were tested in the dry and wet experiments. White bottle tops were used in the dry measurements whilst blue and white cut-out pieces were used for wet conditions. Although a decrease in reflectance due to presence of water could be expected, we must consider that the magnitude of reflectance is also dependent on the different colour of the targets, as previously observed. Dry LDPE at 100% had higher reflectance, which can be explained by its transparent colour acting as a specular reflective surface. However, once underwater or submerged water, LDPE absorbs most of the light hence lower reflectance. It is worth mentioning that in some polymers, and in LDPE in particular, thickness may play an important role, due to the transmittance properties of this material. This aspect, however, was not investigated in this study.

Detecting new plastics would therefore be possible but wet and clear LDPE litter would require a sensor with a high signal-to-noise ratio, especially if it is submerged. New plastics are more water-repellent, which means if they can float, the detectable reflectance will not be greatly affected by absorption of light by water. During the experiments,

samples were kept wet by using a spray bottle. PS showed the least reflectance loss due to presence of water, especially at 850 nm (Fig. 6 and Table 2 in Garaba et al., 2021[3]).

The anisotropy of the spectral reflectance of the raw plastics was confirmed in the experiments. The change in reflectance can be attributed to the orientation of the samples relative to the viewing angle. Some of the challenges to fully understand the effects of anisotropy for the plastics is the roughness of the surfaces, the inherent optical properties of the polymers, and the potential contribution of the surrounding optically active materials to the bulk spectral reflectance observed. At different wavelengths for each polymer, the spectral reflectance varied with the observation angle (Fig. 6 and Table 2 in Garaba et al., 2021[3]).

The spectral reflectance measured for the different plastic samples is clearly dependent on multiple factors. For example, dry HDPE at 850 nm showed reflectance varying from 0.408 at 0° nadir viewing angle to 0.506 at 30° nadir viewing angle, but decreased at 45° nadir angle to 0.498. However, dry LDPE reflectance decreased from 0.106 (0°) to 0.028 (45°) at 100%-pixel coverage. The expected decrease of reflectance when dry samples are wet was not always observed (Table 2 in Garaba et al., 2021[3]). The Absolute Percentage Differences (APDs) in LDPE actually increased (turned positive, suggesting increase in reflectance) with nadir observations angle from -64% at 0° to 78% at 45° at 850 nm this correlation was also noted at 1215 nm and 1732 nm. However, this was not the case for HDPE, PP and PS. These polymers showed rather minimal changes in APDs with the varying observations nadir angle as well as a general decrease in APDs (negative, suggesting loss of reflectance in water).

Spectral measurements of the wet marine-harvested plastics were completed at 100%-pixel coverage which were recalculated to 76-100%. For all the materials tested in the experiments, the wet samples had a generally lower reflectance compared to the dry samples. Raw PS and marine-harvested foam showed reflectance > 0.1 at all wavebands. This could be attributed to the fact that most of these samples were highly floating, and that the white colour common in these targets contribute to increase the total bulk reflectance.

Marine-harvested plastics

The degree of biofouling of the plastics was investigated by also measuring the reflectance after cleaning the samples and at times by performing further FT-IR analysis before and after cleaning. Spectrally, we did not observe large differences for these samples. It was noted that detection of hard-type plastics at pixel coverage < 20% could be challenging, as the reflectance was higher than expected, which could be an effect of smoothing and of a weak signal reaching the sensor.

Reflectance of the wet marine-harvested samples decreased with an increasing observation nadir angle. Unlike for the raw materials observed outdoors, it could suggest that the assumed diffuse light conditions indoors were not similar and comparable to those of ambient sunlight. An apparent result was that the reflectance of all samples at 0° nadir was highest, most noticeable at 850 nm and least at 1732 nm.

Apparent pixel coverage

In addition to the above procedures, all the measurements (including pixel coverage) were recomputed with the help of the RGB images obtained for each test. The aim was to correct the results and data according to the true coverage of the FOV. The fractional pixel coverage was computed as the ratio between sample area and background area within the sensor swath, derived from sample outline in the RGB images.

Possible extensions and improvements of the lab experiments

The field experiment imposed several challenges that become lessons learned, as well as possible extensions of the exercise done here. We list them below, for their consideration in future work:

- The experiment provided new insights in terms of spectral measurements of plastics.
- Special care should be taken when taking indoor measurements. By using a suboptimal light source and power source can introduce noise which is challenging to parameterize. Yet, outdoor measurements can also prove challenging due to unstable atmospheric conditions and a limited time window of the suitable solar elevation. It is recommended to collect outdoor measurements in a geographical location with long hours of sunlight and stable atmospheric conditions.
- The black background material could be improved by extensive measurements of available materials such as Krylon Black® spray paint, light absorbing black materials or Acktar® black coating as background.
- A higher nadir angular resolution could help improve our understanding of the spectral reflectance dependence on viewing angle.

- The variability of the sample positions could be reduced by using fixed frames to hold the sample preparations.
- More insights on the effect of water depth can be obtained by varying and recording the depth of the water layer covering the samples, for example, using a fixed frame to adjust the water layer (depth).
- Geometry analysis could be restricted to common satellite nadir viewing angles to further understand how waves and orientation of the plastics relates to spectral reflectance.
- To improve the quality of photographs, a non-polarizing filter could potentially mitigate reflections on the water.
- Indoor measurements should be conducted after an extensive characterisation of the dark room and light source.
- Ocean bio-fouled samples could be cleaned, and the spectral reflectance measured for better understanding how biofouling affects light properties of plastics.
- Raw materials could be exposed to controlled doses of UV light to explore the effect of photo-degradation on optical properties. In this regard, a potential experimental setup was already proposed by Efimova and co-workers[62].

S3.1.2. Radiative Transfer Modelling experiments

Rationale of the modelling

The experimental parameters for the modelling was selected according to what was deemed as the best potential target for ML observation from space, *i.e.*, ocean waters with low content on sediments, not affected by land aerosols, and at depths larger than Secchi disk depth to prevent contribution from the sea bottom.

A priori, it could be possible to achieve measurement of ML by using plastics as a proxy, as it composes a vast majority of the total ML, particularly in surface waters[15]. In terms of ML concentrations, the highest accumulations are found on the coasts and nearshore seafloor[15]. However, ML, and even the plastic fraction of the ML, is a very heterogeneous mix, which imposes strong limitations in how the problem should be approached.

The problem of targeting plastic litter from space is even more challenging in nearshore waters and on beaches. There are a few reasons for that, like the wide range of variability of additional variables influencing the remote-sensing reflectance, such as the Coloured Dissolved Organic Matter (CDOM) associated with productivity of the nearshore waters (and/or by input from rivers), sediments in suspension, aerosol optical thickness (AOT), etc. Moreover, increasing heterogeneity in the composition of the ML also increases towards shore[15]. While floating plastic litter in offshore waters is predominantly made of low-density polymers (PP, LDPE and PS), the diversity of items and polymers increases towards the coast[63]. The maximum diversity of polymers is often found over beaches. This is in addition to the wide variety of background conditions in the shorelines, including but not limited to whether the coastal area is rocky or sandy, the size of the grains and stones, the composition of those, the slopes of the coast, etc.

Considering all these aspects, we concluded the best chances for detection are concentrated over waters not too close to the coast, and where floating litter could form dense accumulations or patches. Studies on the macroscale distribution of plastic debris are widely reported in the scientific literature[16-18,64]. We know less about how litter accumulates in the surface at mesoscale and submesoscale, where most EO optical sensors operate (below 1 km in spatial resolution). Under certain conditions, the activation of submesoscale convergence zones on the ocean surface can lead to the aggregation of floating ML in significantly high concentrations (*e.g.*, LWs[11]). In fact, studies performed with S2-MSI prove that it is possible to detect metres-sized ML accumulations when the conditions are suitable. One of the key caveats of this approach, however, is that we do not know how often these accumulations take place, and how representative they are of the total presence of ML. Diffuse contamination could be hard to detect unless a considerable improvement in sensitivity of sensors takes place. However, as part of this simulation, we were able to compare the impact that very small concentrations of litter can have over the water-leaving radiance, which could be well within the range of natural variability or background noise, preventing any detection despite improvement in the technology. Bottom line clearly lies on the SNR defined by the photon noise versus the ML signal.

The last point worth mentioning is about the spectral information. Identifying ML with optical sensors will require the use of bands able to classify unambiguously what is plastic from other components (*e.g.*, floating seaweeds, foam and white caps, sediments, phytoplankton plumes, etc). Existing studies over plastic polymers point towards NIR/SWIR bands as optimal for plastic detection and identification. Not in vain, FT-IR methodologies are currently the state-of-the-art in terms of plastic identification, and they were used for long by the industry for such tasks, especially for rubbish classification and urban wastes processing plants. Henceforth, it makes sense to check these regions of the spectrum to try to find plastic-specific features that could help in the discrimination exercise.

Nonetheless, we may anticipate that using these regions will be a limiting factor for what technologies could achieve in terms of detection. Hyper-spectral technologies may be of interest in EO4ML, as the bandwidths can be narrow enough to discriminate between polymers, but at the cost of losing sensitivity. In principle, it will be more appropriate to go for a super-spectral solution with bands well located over the spectral features specific for the most common polymers in the ML. Garaba and Dierssen showed how the spectral features over raw plastic polymers are considerably close to each other[22]. This was also supported by the retrieval algorithm proposed by Goddijn-Murphy and co-workers[32,61]. However, when trying to apply this theoretical model over field measurements obtained with an ASD, they found that it was not possible to do proper estimations of the polymer composition and/or concentrations[32].

Regardless, in the present simulation we proposed a hyper-spectral line computation to obtain results comparable to those provided by an ASD. These results could be particularly useful to take into account the effect of the atmosphere or the observational geometry, aspects not fully considered in the laboratory measurements.

Objectives of the modelling experiments

In this modelling exercise, we limited the analysis to a set of optimal and sub-optimal case studies, which help to delimit the best chances for a potential EO4ML mission. We also explored different drivers that could be of relevance for any future mission, like impact of wind speed, observational geometry, and *Chl-a* content.

Identifying the minimum concentration

By checking through the modelling outputs, we expected to have a first estimate of the minimum detectable concentration. Given that plastic polymers have different reflective/absorption capability, various composition scenarios were accounted for.

Optimal distance to target

Another aspect to resolve was the maximum amount of intervening atmosphere allowed to preserve the plastic signature within the water-leaving reflectance. The simulation tested this upper threshold by considering different altitudes or distances to the target.

Best geometry for observation

The existing issues with sun glint contamination within the NIR/SWIR bands may prevent any effort for observation. Using S2-MSI images, we found that sensors with high spatial resolution suffer from speckling pixels containing glint outside the usual geometry, as a result of the sides of the wave slopes, leading to a probability of creating specular situations with respect to the sun. Research done in this matter concluded that no fully efficient solution currently exists to tackle this pixel-sized glints. It is, therefore, relevant to consider the geometry of the observation and investigate the impact it could have in the results. An EO4ML mission should also have in mind strategies to minimize this problem by, for example, playing with the Relative Azimuthal Angle (RAA) to minimize glint.

Isolation of spectral bands

As part of the exercise, we expected to identify the best spectral bands or regions that could be employed in a theoretical mission. Traditionally, band selection for features identification is made on the knowledge of the spectral signature of the target, quite often by combining bands where target absorbs/reflects differently than the background, and use a combination of them to construct optical indices to classify the image, in first instance, and quantify it, in second instance.

In the present approach, we opted to carry out an exercise allowing the bands identification through the results of the simulation. The bands identified by laboratory analysis could be non-optimal for an EO application, because outdoor environmental conditions and propagation through the atmosphere are not taken into account.

Additional environmental parameters

We also aimed to provide a first approach to understand the role some parameters have into the resulting water-leaving radiance. The main ones under our current scenario are chlorophyll concentration and wind speed. Both affect how light is reflected by the water's surface and its absorption.

Results of the modeling experiments

Scenarios of reference

The reference scenarios allowed analysing the background signal to be expected in the scenarios with ML. Fig. S13 shows a summary of results for one of the scenarios matching the Case I waters, identified as optimal for the simulations. In this case, L_W diminishes as SZA increases. The observed reduction of L_W for longer wavelengths matched both the lower intensity of the source and the higher water absorption and scattering. The case of pure sun glint (SZA=0º, as we set VZA=0º), however, is a trivial solution of the model that is not useful for our purposes. Because of this reason, results at SZA = 0° were discarded for the analysis of the tested polymers. It is noteworthy to mention that this pattern is only valid for the case shown here (low wind speed), as the ocean will be a quasi-Lambertian surface. Under such a situation, as SZA increases and we depart from the specular direction, L_W values will be smaller systematically.

One important aspect of the reference scenarios is that they showed how SZA, RAA and consequently VZA, matter for the potential observation of marine plastic litter. Light in the visible spectrum is significantly more intense than in the NIR or SWIR, which means that observed L_W is significantly lower at longer wavelengths. This also makes more obvious the negative impact of glint on detection. Litter present in pixels mixed with free water surface could be overlooked under specular view conditions, due to the higher contribution of the glint to the total reflectance than that of the litter itself. RAA could also be used to further minimize the sun glint impact. However, a trade-off between light intensity and glint must be taken into account.

The total uncertainties obtained for the simulations are shown in Fig. S14. Those regions of the spectra where molecular and aerosol scattering are larger have also larger uncertainties, as expected. Atmospheric absorption windows around 1375 nm and 1875 nm, are also yielding significantly larger errors, as expected too. However, it is important to note that the relative impact of these uncertainties can only be measured as the relative weight they have with respect to the signal itself. Thus, noisier bands must be considered in relative terms, considering the level of noise vs. the level of the signal. In other words: what matters is the SNR and not the absolute noise level.

Not surprisingly, results are somewhat too optimistic if we examine the SNR resulting from the reference scenarios (Fig. S15). This is due to a few elements missing from this exercise, mainly the lack of propagation of uncertainties associated with the receiving sensor and a proper estimation of the glint-induced noise. The results at SZA = 15 º show the strong impact of sun glint in the signal. These low angles have a significantly larger contribution of directly reflected radiance than water-leaving emissivity, leading to larger SNRs, particularly in the longest wavelengths. The origin of this effect has to be on how σ_{ref} is computed, which is a combination of photon noise and of the standard deviation of the 10 test runs done for each scenario. In the case of SZA=15º, the latter is significantly more stable and of lower value than as we increase SZA towards wider angles. This is explained by the role aerosols have on the variability of each run. Being this a probabilistic model, the longer the optical path is, the more likely that photons get dispersed by aerosols and absorbed by the atmosphere. This reduction of photons with also a larger dispersion probability leads to larger variability between runs of the scenarios for the larger SZA values. Further proof of this effect comes from the behaviour of the total uncertainty for SZA=15º as a function of the wavelength. With a shortest optical path, molecular scattering and absorption are more relevant, as aerosol scattering is lower. With molecular scattering being more relevant for shorter wavelengths, uncertainties decrease from VIS to SWIR. This effect is translated to the SNR (Fig. S15).

This is a relevant outcome, as it indicates that water-leaving signals under sun glint conditions will be contaminated damping the signal induced by ML in the NIR and SWIR regions of the spectra, where plastic litter is expected to have a distinctive signature. For that reason, wider observational angles will be required. Importantly, optimal results were found when SZA> 45°. In terms of a future mission, a setup of a Phase Angle (PA) = 45º. Typical values of SZA=30º and VZA=15º as used in OC missions will meet this requirement outcome[39]. In the simulations we obtained a PA=45º using SZA=45º and VZA=0º for a downward looking sensor, which in geometrical terms of the observation are an equivalent setup. Henceforth, the results presented for litter scenarios will correspond to an SZA equal to 45°.

Another aspect to notice, which further confirmed the results for the reference scenarios, is the presence of the atmospheric absorption windows. They appeared as deep troughs in the SNR, from 1365 to 1415 nm, and from 1815 to 1950 nm. The strong absorption in these intervals is well known, being excluded for ML detection. They may be, however, used for other purposes, like cirrus detection in S2-MSI (band B10 is set on 1375 nm). No doubt any potential mission based on spectral data shall have additional bands for the sake of atmospheric correction or for discriminating false positives.

Plastic polymers

Our results covered the three most abundant plastic polymers in ML, namely PE, PP and PS. They compose more than 70% of litter present in coastal waters, and run up to 85% or more in offshore water[65]. The simulations used SZA values of 45°, covering surface plastic coverages (*f*) of 0% (reference scenario), 0.1%, 1%, 10%, and 100% (pure polymer spectra). Note that discussion on results below is excluding the analysis of visible bands. They are sensible to the actual colour of the plastic items under observation, and thus, not specific for plastic polymers. However, their use could be important for discrimination of other floating matter that might share spectral features with plastic in NIR and SWIR regions (*e.g.*, floating seaweeds).

Low Density Poly-Ethylene (LDPE)

LDPE showed an increase of radiance in the visible region as *f* increases (Fig. S16). This finding may be explained by the fact that raw polymer spectra were obtained from white items, which significantly increased reflectance in such bands. We also found higher levels of noise in NIR and SWIR regions as *f* increased. Indeed, plastic items reflect and scatter light in these bands, whilst water is more absorbent, causing an apparent reduction of the noise levels.

Regarding SNR_r, there was a strong signal in visible bands (Fig. S17), as expected. SNR_r decreased towards longer wavelengths due to the higher noise with respect to L_W. Different SNR_r peaks were found at 826, 912, 972, 1103, 1160, 1326, 1501, 1589, 1612, 1642, 1782, 2031, 2080, 2160 and 2330 nm. Some of them remained for different *f* values, but others became less obvious, and hence, less useful.

The most characteristic peak was at 1326 nm, around which a relatively wide band was defined. This peak was sensitive to the fraction of LDPE (f_{LDPE}), decreasing for f_{LDPE} at 1% and 0.1%. This means such a band could be used not only for detection, but potentially also for quantification. First peak in NIR was found around 826 nm. It was relatively weak with respect to the overall signal magnitude in that spectral region, but it could still be used for detection purposes. Other peaks of interest were around 912 nm and 972 nm, with distinguishable features even at low f_{LDPE}. On the other hand, bandwidths are very limited and there was a strong influence of the atmosphere in the observed signal, sharply decreasing towards TOA. The peak at 1160 nm could also be used, but quickly loses sensitivity at low f_{LDPE}. In the range of 1450-1700 nm, there are a few interesting spectral features, particularly SNRr peaks at 1501 nm, 1589 nm, 1612 nm and 1642 nm, with the last two corresponding to narrower bands. These last two peaks were even present at the lowest f_{LDPE} (0.1%), being suitable candidates for detection and quantification. Finally, a weak peak was found around 1782 nm, and other candidate bands at 2031 nm, 2080 nm, 2160 nm and 2330 nm. Here, a potential wide bandwidth could be used to define such a spectral region and increase sensitivity in its observation. However, this region showed low SNR_r, becoming hardly visible at low f_{LDPE}.

The results provided insight into why certain levels of detection can be achieved with S2-MSI data. Its bands B8 (at 832 nm, range from 779 to 886 nm) and B11 (at 1610 nm, range from 1565 to 1655 nm) are set into areas with a certain degree of sensitivity to the presence of plastic. SNR_r in band B11 could be low enough to be monitored properly, so detection capability may depend on whether it is within the sensitivity levels of the sensor. Also, band B8 in S2-MSI is probably too broad and unspecific for proper monitoring, being likely also driven by other components in the surface water. Even if both S2-MSI bands could be used, there is a strong limitation to spectrally distinguish litter from other components. However, a narrower band around 832 nm, with the appropriate sensor sensitivity, has potential and could be considered, as well as the use of dedicated spectral bands in the VIS.

Polypropylene (PP)

PP showed abundant spectral similarities with LDPE, as previously reported[3,22]. Figs. S18 and S19 show that the main differences were related to the height of some peaks. For PP, the best positions were found at 826, 912, 972, 1103, 1159, 1326, 1502, 1535, 1573, 1642, 1782, 2031, 2080 and 2150 nm. SNR_r peaks at 826 nm, 912 nm, 972 nm, 1159 nm, 1326 nm and 1642 nm positions matched with LDPE peaks, but somewhat stronger for PP than for LDPE, probably due to the larger transmittance in LDPE than PP. Likewise, there was a stronger peak around 1782 nm, being now apparent at low f_{PP}, leaving room for the possibility of specific PP detection. Some other peaks were somewhat displaced. Thus, the maximum at 2160 nm for LDPE was around 2150 nm in the SNR_r spectra of PP, showing also better definition than in the LDPE spectra.

Other peaks, however, were no longer as evident or meaningful as for LDPE. The maximum at 1612 nm was not so apparent, being now shifted to 1573 nm. A new peak appears for PP at 1535 nm, but the one at 2330 nm is no longer visible. Again, a band in the 1450 - 1700 nm could cover information for various polymers. This fact lessens the capability to discriminate spectrally between polymers, although this could hopefully be done with a high spectral resolution and if enough SNR is reached at instrument level.

Polystyrene (PS)

There were, once more, many spectral similarities in terms of SNR_r between PS and LDPE-PP, together with some important differences (Figs. S20 and S21). For PS, the main peaks were found at 826, 915, 972, 1098, 1217, 1324, 1500, 1528, 1573, 1782, 2031 and 2251 nm.

Peaks at 826 nm, 915 nm, 972 nm, 1159 nm, 1324 nm, 1500, and 1782 nm were common to the three polymers, but some dissimilarities were apparent in other wavelengths, opening opportunities for spectral differentiation. Bands at 1612 nm and 1573 nm were specific for LDPE and PP, not being present in the PS spectrum. On the other hand, PS showed a distinctive peak at 2251 nm. Likewise, the band at 2080 nm was present in both LDPE and PP but not in PS. Additionally, the band found for PS at 1217 nm did not appear in the other two. In other few cases, some of the bands showed a stronger peak in some of the common bands. For example, the strong signal of the peak at 2030 nm for PS, quite close to a peak observed for PP, was particularly noteworthy. A band in such a spectral region appears as a good candidate to detect floating plastics. The new local and well-defined maximum at 2251 nm will be potentially useful to identify pixels with high PS coverage, as SNR_r was low.

Possible approaches for the EO4ML mission

Based on our results, it would be feasible to develop a superspectral mission devoted to the detection and monitoring of floating plastic debris in the ocean. The results showed significant values of SNR_r at pixel coverages (f) of 10% for the three polymers analysed, LDPE, PP and PS. Spectral differentiation from respect to background could be also achievable at coverages around 1%, but this capability would be pretty much lost at coverages in the order of 0.1%. This implies that monitoring floating microplastics is beyond reach for the current concentrations in the ocean, even if those are just on the surface, as per setup of this modelling activity. The minimum threshold of 1% found in our analysis is orders of magnitude above the marine microplastic concentrations[20]. It is important to highlight that our lab experiments also showed this specific limitation, with signature below 1%-pixel coverage lost due to the background noise. Lab and modelling results converged on the same conclusion. A realistic scenario should rely on the monitoring of dense accumulations of plastic debris on the ocean surface, which uses a combination of adequate spatial resolution and sensor sensitivity for the selected spectral bands.

The spectral differentiation of main plastic polymers could be achieved by means of a proper selection of the spectral bands and spectral unmixing techniques. This capability will require the definition of some narrow, and somewhat numerous, bands to prevent the overlapping of peaks within the same bandwidth, which shall be put against the requirements for sensitivity. On the other hand, the three polymers analysed here shared features in different spectral regions. These overlaps could be used to define relatively broad bands for a generic detection of plastics material and try to increase the instrumental SNR. This implies the sacrifice of the capability to discriminate between polymers in order to achieve detection at lower concentrations or with higher spatial resolution.

Another important aspect to be considered is the possibility to use ancillary bands in the VIS-NIR to discriminate or rule out false positives. While, for instance, driftwood, phytoplankton and floating seaweeds may contribute to some of the peaks identified for plastic polymers in the VIS-NIR[47,48,57], it must be noted that much of the ocean plastic litter is white or of pale colours, as result of production choices but also due to discoloration by weathering[40]. This suggests that bands in visible light could be used to discriminate plastic polymers from other concurring elements, which could also have similar spectral features in NIR and SWIR. For instance, red, green and blue bands could help to discriminate between floating seaweeds[41], land-sourced organic debris (*e.g.*, reeds) and plastic litter. White caps, generated under strong wind conditions, would still remain a problem, although the possibility for discrimination against additional bands could be possible.

Regarding the changes in the spectral signature from BOA to TOA, both SNR and SNR_r decrease with altitude, as expected. However, this effect was not enough to mask the most relevant peaks (for the experimental parameters of this modelling effort), suggesting that observation could be achieved at orbit level. Exact altitude for the mission will depend on the final GSD and the sensitivity requirements at sensor level. It is important to note that the SNR_r peaks, derived from the modelling exercise, took this into account. Some local peaks were apparent at altitudes below TOA, but disappeared or were irrelevant upon reaching TOA. The present assessment could be repeated with a more precise estimate of the expected sensor altitude. Note that, however, small loss was predicted beyond 32.5 km. This opens up the opportunity of using a platform other than satellites, provided they can meet other technical requirements. Examples of this could be the High-Altitude Pseudo Satellites (HAPS).

All in all, results indicated that ML monitoring can be achieved from space, given the right choice of bands and pending on the instrumental assessment. This monitoring, however, will have some limitations, especially in terms of

observable concentrations. Only dense plastic accumulations will be observed under a medium spatial resolution (1-10 m). However, if enough sensitivity is provided at higher spatial resolutions, detection on individual objects could be feasible (*e.g.*, 10% plastic coverages over 25-cm spatial resolution would, in principle, provide observable anomalies in the measured L_w). Hence, such applications could also be open to more regional or dedicated missions with VHR targets. Of course, if sensitivity is increased, even smaller plastic coverages could be observed. Results at 1% coverage are still meaningful, although SNR_r was low in general terms, which makes detection difficult. "Well-developed" LWs may show plastic coverage of this order of magnitude[11]. Some SNR_r peaks were shared by the three plastic polymers analysed, LDPE, PP and PS. In other cases, they were exclusive to a single polymer. Measuring L_w polymer-specific pikes could help not only to detect, but possibly also to identify, plastic materials composing the observed reflectance. This strategy will be referred to as a fine approach. Unfortunately, many peaks were close to each other, with differences of less than 10 nm in NIR or visible, or 40 nm in SWIR. A mission to identify plastic types would require narrow polymer-specific bands, yet this usually limits sensor sensitivity or GSD. For the fine approach, we identified 19 potential bands (Table S33 and Fig. S22), which should be complemented with additional bands in visible and NIR to enable observation of non-plastic targets that could confuse the detection, as well as any band needed for atmospheric corrections. Alternatively, a broad approach could merge nearby bands, being more efficient in detecting the pool of floating plastics but having more difficulties to classify them. A set of 10 potential bands were identified for the broad approach (Table S34 and Fig. S23), to be complemented with ancillary bands as indicated for the fine approach.

The definition of the bandwidth for both the fine and the coarse (or wide) approach was made on the basis of the following criteria:

- To cover the pike in order to maximize the integral of the spectrum within the defined width, but before the signal decays;
- To avoid spectral overlapping between selected bands, in order to prevent the issues described in previous papers[48].

Our definition of bandwidths must be further refined in future work, *e.g.,* by modelling pikes as Gaussian curves and including 95% of the integral of the Gaussian curve within the defined width (*i.e.* width would be equal two 2·sigma in the Gaussian curve). Ultimately, the exact width will depend on the spectral response of the sensors and the need to achieve a SNR minimum, so it will depend on the technology and should be further re-evaluated during a Phase 0 of the mission development.

Optimal set of spectral bands proposed for EO4ML

Our results were compared with some other existing missions, in particular, with S2-MSI and Sentinel-3/OLCI. This analysis helped to identify the additional bands that could be used in an EO4ML mission to perform (*i*) atmospheric correction, (*ii*) aerosol correction, (*iii*) false positive discrimination and (*iv*) plastic detection and quantification. A total of 23 bands configured in a super-spectral sensor (or complement of sensors) suffices to achieve a successful mission. It is important to mention that we do not propose a specific retrieval algorithm here; the set of proposed bands (Table S23) will provide the necessary information for a spectral separation of classes within a pixel using spectral unmixing techniques or band ratio indices. Additional work in an end-to-end (E2E) simulator would be also required to investigate the optimal retrieval algorithm.

Another aspect to consider is the fact that some of the identified optimal bands can be significantly impacted by water content in the atmosphere. However, some of the proposed bands (B11 and B13) can be used to estimate such atmospheric contributions and remove them from all bands by means of an inverse model, for example. Thus, a dedicated atmospheric correction, with consideration to these aspects, could make possible the use of otherwise contaminated bands, as well as improve the signal in others.

For our EO4ML proposal, we consider that the coarse solution (Table S34) is more likely to meet the SNR requirements and spatial resolution needs than the fine solution. However, we decided to readjust some of these widths to reduce overlapping between them. As mentioned above, those bandwidths shall be refined in further studies, according to technological compliance to target SNR values. Here, we allowed for overlapping for the case of bands dedicated to different purposes. For instance, bands B07, B08 and B10 overlap with B09, however they are used for atmospheric characterisation and correction, whereas B09 is proposed for plastic retrieval.

Importantly, proposed band B15 is useful for the identification of plastic polymers, but shows limited atmospheric transmittance. Because of this, atmospheric correction will be critical, as it will be necessary to properly exploit such bands. Note that the choice of bands may also support other types of applications, like ocean colour and biodiversity

studies. Moreover, the set of bands could be suited for monitoring other water pollutants and indicators, such as oil leaks or harmful algal blooms (HABs).

It is noteworthy to mention that some of the bands we propose are close enough to the atmospheric absorption windows, which means that the signal measured with them could be hampered by atmospheric effects. In particular 1325 nm is a very promising band but quite close to one of the aforementioned windows. For that reason, special care shall be taken when considering such bands for a mission. Nonetheless, under the idea of using spectral anomalies for ML detection, such bands will still yield information, as the water signal would be equally impacted by such a problem and thus, anomalies will still be relevant over concomitant observations in these bands.

S3.1.3. Findings of the experimental plan

The spectra of raw and marine-harvested polymers did not show significant differences, likely in part because of the specific set-up and samples used. Whilst some marine-harvested items were biofouled, their spectral signature was only significantly affected if a major fraction of the plastic surface was covered by biofouling, which occurred in a small fraction of samples. Indeed, heavily biofouled items tend to sink by ballasting. In addition, it should be noted that biofouling organisms are usually attached on the submersed part of the floating objects, as UV light and dryness prevent the development of many life forms on the emergent part.

Obtaining distinguishable signatures of plastic litter with pixel fractions below 1% was highly challenging, a result further confirmed by the modelling simulations. The reason was the large effect of the experimental photon noise compared to the target signal.

Plastic colour played an important role in terms of reflectance level, especially in the visible bands, but an effect on NIR and SWIR was also observed in the lab measurements. This dependency can be associated with the pigments used to colour the polymers, but this was not confirmed. The fact that floating plastic is predominantly white or close to be, it is an opportunity to support discrimination between plastic and concurring materials (*e.g.*, floating seaweeds, driftwood).

No clear dependency of the observation geometry was concluded from the lab measurements, pointing towards the presence of Lambertian properties of the polymers when dealing with light. Geometry of observation in the modelling did have effects, as result of the trade-off between light intensity and glint. A 3D scattering could be expected as a consequence of the shape of the pieces, which will have a net effect mainly in the TOA. This particular aspect should be further studied.

There was a strong effect of layers of water over the plastic pieces, in agreement with the previous studies[22,32]. However, such strong attenuation could not be explained by very thin layers of water, as per the experimental setup performed by Garaba and co-workers[22]. Similar behaviour was found in our lab measurements. On the other hand, according to the literature, it is likely that the refractive index at the plastic-air interface is quite different from that at the plastic-water interface, which would largely affect the angular dependence of light source and sensor position. Our measurements with wet polymers at varying angles showed similar behaviour than those reported by Voss and Zhang[31], which points to this particular issue. Further research in this field would be helpful to improve detection of subsurface plastics.

Modelling exercise was focused in Case I waters, which potentially present the best conditions to detect floating plastics. The three most frequent floating polymers were studied (LDPE, PP and PS) to understand the changes in their spectral signature from BOA to TOA with pixel coverages (f) of 0.1%, 1% and 10%. The exercise involved the development of combined BRDFs for seawater and floating plastics. We found detection possibilities from 1%-pixel coverage upwards.

As pixels in the simulation are size-agnostic, the detection success for pixel coverage at 1% and 10% should be revisited according to the SNR specific for any candidate sensor. The current results were based on a SNR_r index that measures the difference between the signature of clean ocean and litter scenarios. This approach relies on signal recovery through spectral anomalies obtained from subtraction of the background signal. A similar approach was proposed for ML[47], and recently applied for retrieval of *Sargassum* sub-pixel concentrations[66,67]. Nevertheless, as enough sensitivity is achieved, GSD could be adjusted accordingly to increase mapping capabilities.

Detection and monitoring low-level concentrations of plastics per pixel (whether from microplastics or macroplastics) proved to be unfeasible because of the minimum concentrations required to obtain a measurable signature. Thus, any

potential satellite mission will have the focus on the observation of plastic aggregations of extraordinarily high density, like those described on the ocean surface at the submesoscale domain[11]. The level of detail that can be achieved will depend on the GSD that can be found compatible with floating plastic detection.

No special loss of signature in the relevant bands was found when propagating signature from BOA to TOA, although at TOA level, the number of potential bands diminishes as result of the atmospheric absorption and scattering.

A set of spectral bands with potential for floating plastic detection was defined, pending on instrumental assessment and retrieval algorithm definition. The abundant number of bands offers diverse options for floating litter detection and monitoring. It should be noted that classification may be difficult to achieve, as not all bands will yield enough SNR, or they may require GSDs that are not compatible with the targets.

Geometries of the simulations have resolved that glint presence contaminates potential observations to the extent that distinguishing glint from plastic polymers can be unfeasible. This effect could be mitigated by including specific absorption bands for the polymers. Nonetheless, and similar to ocean colour, a PA between 40° and 60° shall be favoured, as it provides a good combination of low glint levels but enough light intensity. Glint can appear at higher spatial resolution as a consequence of the slopes in the sea surface caused by wave trains (usually < 100 m). Additional deglint techniques could be required in these cases.

Modelling results helped to understand why S2-MSI can detect certain accumulations of floating ML. Bands at 832 nm and 1610 nm were between those yielding relevant SNR values for litter observation, although specific band central wavelengths, bandwidths and GSD from S2-MSI are not optimal for plastic observation, plus there is evidence of spectral distortions within the S2-MSI measurements coming from the unmatching spatial resolution between the bands making difficult the discrimination between spectral contributors to the reflectance[47]. In addition, lack of more SWIR bands applicable to this purpose in S2-MSI renders it difficult to distinguish between plastic litter and other spectral end members.

Atmospheric correction is one of the key points to tackle in the future. As per this experiment, most of the relevant signal from plastic polymers was found in NIR and SWIR bands, which quite often are used in ocean colour applications to perform vicarious atmospheric corrections and/or calibrations: the technique would effectively remove signatures in these spectral regions to correct information in the bands used for ocean colour. As a consequence, a different atmospheric correction approach shall be found for floating plastic observation.

S3.2. Mission Concept and way forward

S3.2.1. Revisited applications and mission requirements

A starting set of applications was identified from the input of the scientific community with interest in ML. In addition, a set of mission requirements for each of those applications was defined. The strategy was focused on cross-checking mission requirements and applications, with special attention to the most realistic solution according to the assumptions and results obtained.

In this section, we revisit the applications and requirements, and compare them against the technological approach and performance. Thus, potential applications are related to the capabilities of the proposed EO4ML mission concept to find out what this theoretical mission could cover.

Proposed mission specifications

A summary of the mission specifications is shown in Table S35. Some of them could be adjusted to further extend the applicability of the EO4ML mission, however, they are a good starting point to address this issue in the future phases of the study. It is worth mentioning at this stage that many of the mission specifications were derived from S2-MSI. This was intentional, as we consider that a mission of such characteristics can achieve many of the objectives for a specific EO4ML mission. Nonetheless, different aspects could be reconsidered or evaluated again once more information becomes available.

Some of the parameters that could be subject to revision are the altitude of the satellite, which for the time being is set at the same orbit of Sentinel-2. But there are reasons why reducing the altitude could be suitable. On one hand, it could help to improve the SNR, which would help to reduce the spatial resolution of the instrument and thus improve the applicability and validity of the measurements. Also, a lower orbit would mean that time used in each orbit would

decrease, although at the same time the effective swath would be reduced and more motion-induced image blurring would need to be compensated

S2-MSI is significantly impacted by glint, as this was not an open-ocean mission. As explained in this document, sun glint can be a source of considerable problem for the effective detection of accumulations of marine floating plastic. Some parameters could be also revisited to minimize the glint.

Applications covered by the proposed mission

Considering the requirements and the capabilities of the proposed mission, we can establish which of the proposed applications could be potentially covered. One of the key elements to take into account is that initial estimations set the required sensitivity in 1% of the pixel coverage. This translates to objects or equivalent areas of 4 m^2 and 1 m^2, for the 20 m and 10 m resolution options, respectively. These thresholds impose strong constraints for detecting sparse objects on the ocean surface. However, if the spatial resolution were to reach 5 m, objects or equivalent surfaces of 0.25 m^2 could be detected. That would be possibly enough to detect plastic buoys drifting in the ocean. Nonetheless, such spatial resolution would require additional technological development to preserve the requirements in terms of SNR.

Table S36 lists applications and key mission requirements for the different proposed spatial resolutions. It would seem none of the possible applications is compliant. However, there are certain levels of acceptance in some of them that would require further revision. For instance, “Detection and monitoring of hot spots and accumulation zones” could be fully achieved by shortening the revisiting time of the instrument. In fact, in this particular case, it is already covered over 30° in latitude, and assuming two twin satellites in orbit (as currently is the case for Sentinel-2). The same applies for “Monitoring of river mouths as main input flow for ML”, in which the limiting factor is the revisiting time[68], but a threshold level could be reached by using multiple platforms, for example.

In the case of the “Detection and identification of large ML items at the ocean”, the same limitation applies in terms of revisiting time, although that main constraint lies in the lower limit of detectability. We estimate that this could be achieved at spatial resolutions of better than 5 m, assuming that detectability is the only criterion.

For the application “Detection and quantification of concentrations of ML at global scale”, most of the parameters are favourable. However, the instrumental sensitivity that is required does not fall within acceptable values. According to our results, concentrations of ML below 1% are almost indistinguishable from the background photon noise, rendering this application not feasible.

The “Detection, monitoring and quantification of ML at shores and beaches” application has not been properly explored here, as its complexity was considered too high for the resources and time availability. Preliminary analyses showed that satellites would have serious difficulties in detecting plastic on beaches, due to the strong radiometric contamination that sand, gravel and rocks produce. Indeed, the main advantage of using NIR/SWIR bands is the strong absorption of water in these bands, in comparison to the plastic polymers, which puts on the table the strategy of using spectral anomalies for detection and eventually quantification. This advantage, however, does not exist over beaches, as these other materials can be significantly more reflective than plastics, and the combination of wet and dry surfaces adds additional complexity. Other technologies could operate well in these environments (*e.g.*, drones or VHR satellites operating in VIS).

S3.2.2. Development Plan

The present section provides a roadmap for technology developments aimed at raising the maturity of ML remote sensing techniques, leading from the mission conceptualization to higher TRL value. It also identifies those areas where additional effort could result in significant improvements of the proposed mission concept.

Pathway for a breadboard

One of the main objectives of the present work is to demonstrate the observability of ML from space. In the perspective of building observational requirements for a future mission, the main outcomes of the study were:

- Focusing on ML surface concentrations in the ocean, we identified multispectral optical imaging as one of the most promising techniques, implying the need to optimize the instrument for ML spectral bands.
- Based on a thorough experimental exercise, we provided the candidate spectral bands for the detection and identification of floating plastic polymers most commonly found in ocean waters.

- We found that a concentration of 1% of floating plastic can be detected by a superespectral instrument, with a GSD of 20 m (with current S2-MSI like mission) and down to 10 m (with the next generation of MSI).

The present study is the first comprehensive effort in performing the iteration between (*i*) acknowledgement of the ML problematic, (*ii*) identification of scientific needs, (*iii*) mission requirements, (*iv*) observation requirements and (*v*) preliminary performance assessment. This iterative process is the natural path followed by science space missions, as done for Ocean Colour missions or Green House Gases Monitoring missions, for example. Several such iterations will have to be carried out before the implementation of a ML remote sensing mission. It will be necessary to confirm scientific objectives, analyse the feasibility in-depth, and better assess the reachable performances, until the mission and technical requirements can be definitively fixed.

An interesting conclusion from our analyses is that current observation techniques provide a good starting point for testing the detection capability of the floating ML. In the short term (*i.e.*, unless further analyses demonstrate other requirements), the current technical roadmaps, in particular for detectors, meet the needs. The next generation of multispectral instruments would be well suited for an EO4ML mission, provided that the appropriate bands and post-processing methods are worked out.

The preliminary requirements derived from the present study do not permit (or justify) the identification of development activities for the instrument technologies. It is reasonable to be confident that the current roadmaps will be able to provide natural candidate technologies for such a mission. In particular, two technologies will be key to the future of the mission, detectors (VIS / SWIR), and spectral filters (VIS / SWIR).

The investigations on the indices used for ML detection should be further pursued. We proposed here some promising indices, but more and possibly more complex indices (*e.g.*, involving more spectral bands) could likely lower the required SNR or detect fainter concentrations than 1%. Moreover, the retrieval need not to be constrained to spectral indices. Spectral unmixing techniques could be particularly useful, in particular if a large number of bands is involved. Synergies with the Ocean Colour community, which has performed similar work in the field of plankton observation, for instance, are feasible.

The simultaneous exploitation of field information could be beneficial for the consolidation of the processing techniques, for the testing of the detection methods (*e.g.*, candidate indices), or for the refinement of false-alarm removal methods, for example. Three European hyperspectral missions have started operating in recent years: (*i*) the DESIS instrument (DLR Earth Sensing Imaging Spectrometer, on the ISS, with a GSD of 30 m and a spectral resolution of 2.55 nm and including 235 spectral bands spanning from visible to near-IR); (*ii*) the PRISMA mission (ASI PRecursore IperSpettrale della Missione Applicativa) with 249 bands ranging from 400 to 2505 nm and 30m of spatial resolution; (*iii*) and EnMap mission, with 242 channels in the range 420 to 2450 nm and 30m resolution. These missions offer opportunities for testing ML detection methods directly in the spectral bands of interest, probably limited to high concentrations of floating plastic (due to spatial resolution). ML aggregations near shores and in river deltas is an interesting research field. They are well-known areas of interest, ML concentrations can be considerably high, and data availability is relatively high (with S2-MSI in particular). Ultimately, like GHG monitoring missions, identifying and surveying ML sources could be of interest for the possible implementation of polluter-pays policies.

Once the requirements are consolidated, industry could refine mission and instrument design and performances. A relevant trade-off will be to compare multispectral and hyperspectral instruments against these new requirements, in light of the feedback from the experience on the latest missions.

The present study opens new ways to advance the remote sensing of ML, as well as raises some interesting questions about the spectral characteristics of marine plastics. It provides insight into the limits of the existing capabilities, and what needs to be improved in order to achieve an optimal observational system. The present section tries to cover the main topics that could be further explored in coming activities and that are of particular interest to advance remote sensing of plastics in the ocean and aquatic environments as a whole.

Requirements for basic research

During the experimental and modelling setups, two main areas required particular emphasis:

- the identification of the lower limit of plastic concentration in a pixel that is feasible to detect with the current hyperspectral analysis capabilities;
- the understanding of the changing spectral properties of the plastic polymers when sub-submersed or just covered by a thin water layer.

Lower limit of detectability

Using a controlled experiment, we determined the smallest concentration of plastic detectable by an ASD. Hyperspectral measurements in this exercise were performed using an ASD in bands of 1 nm (interpolated from the true spectral resolution of the sensor) from 350 to 2500 nm, covering from VIS to NIR and SWIR spectral regions. The measurements were carried out over dry and wet samples of various polymers (*i.e.*, HDPE, LDPE, PP and PS) in concentrations from a 100% coverage of the FOV to 0.01% coverage of the FOV. Both raw materials and sea-harvested samples were analysed. Measurements were carried out with sun light in an exterior setup.

The expectation for this experiment was to determine the limit of coverage in the FOV required to trigger enough signal in the ASD. However, results were rather inconclusive at the smaller coverage fractions. Residual reflectance after subtracting the signature from the background for 0.1% and 0.01% of coverage was almost identical, with 1% of FOV coverage being the case yielding the minimal measurable signature. Even so, at such values, most of the spectral features observed at larger fractions disappeared, being thus the real limit for the ASD found between 1% and 10% coverage of the pixel. The results were even less favourable when measuring wet samples, as the water significantly absorbed light and reflectance values were reduced.

After a first analysis of results, we speculated that the experimental setup could be not good enough to reduce the photon noise in the scene, as a result of indirect light due to reflections in the surrounding ground, the measuring device and the platform. Indeed, the background used in the experiment was not really 100% non-reflective. Thus, if we did not quantify this background noise, we would not be able to provide a range of validity for the lower concentrations.

Regardless of this constraint, we found that the theoretical limit for a meaningful detection would be set in a pixel coverage of 1%, over a set of analysed plastic fractions of 100%, 10%, 1%, 0.1% and 0% (this last one as reference). As in the results of the lab experiments, when simulating the propagation of the spectral signature of the plastic polymers at TOA level, results below 1% showed that plastic presence could not be distinguished from the photon noise. Results of simulations were yielding that the relative SNR of plastic with respect to the reference was below 10 for most of the bands at a fraction of 0.1% of the pixel, which in practical terms means that signal and noise were of the same magnitude, *i.e.*, indistinguishable. This was not mainly due to the levels of aerosols used in the simulation (it was low, as an ideal, optimal scenario was used for that), rather than the proper noise of the light source and of the molecular scattering. Therefore, this seems to validate the results obtained during the field experiment. However, we still need to empirically confirm the 1% limit, and this could compose an activity to be performed.

A repetition of the lab experiment with better controlled conditions would be necessary to:

- consolidate results and discard potential human errors or limitations in our experiment (*e.g.*, try to reduce noise associated with the measurement environment and light source);
- try to determine a more accurate limit for practical detection (*i.e.*, for concentrations below 10% of the FOV);
- ideally, bring into the game potential sensors that could be candidates for a remote sensing mission, or at very least, closer to the characteristics we could expect from existing instruments;
- go further in the characterization of the uncertainties in the measurement, information that would be of great value for future characterization of the spectral signature, simulation exercises and in the definition of the specifications of an instrument specifically devoted to target ML.

Knowledge of the instrument is key in this matter, as it will also help to discriminate between inherent noise and noise coming from external factors. The outcomes of new experiments could be used to improve the modelling exercise, as well as being fundamental for the future steps of the mission. In our opinion, it seems that “closing” the problem within a laboratory setup is a necessary step that was just partially achieved, and thus, requires further attention. Previously, we made some additional recommendations based on the run experiment that could also be considered for this task.

Signature under wet conditions

Our experiments demonstrated the relevance of the wetness of the polymers on their spectral signature[3]. Spectra for wet polymers varied according to the viewing angle and the polymer itself. The impact of the viewing angles was not so evident in the runs with dry polymers, as those yielded differences that were significantly neutralized when adjusting the real percentage of coverage of the FOV according to the varying geometry. However, in wet samples, these values changed significantly, also showing different effects depending on the polymer.

The simplest explanation for this behaviour and for the complexity of the resulting BRDF in the studied polymers could be found in the known hydrophobic properties of plastic polymers. When plastic polymers are wet, there is potentially a gap between water and plastic surfaces that could significantly increase the refractive indices. The results we observed with varying angle points indicated that the geometrical properties of the polymer surface also matter, and that the measurements vary significantly with the viewing angle, in contrast to our findings with dry polymers, where no such behaviour was observed.

Existing evidence suggests that there is a dependency of the spectral measurements on the above aspects, which are not currently covered by any research to our knowledge. These aspects are, however, key for the proper understanding of spectral signatures and how sunlight interacts with marine plastics in the environment. Available observations prove that much of the floating litter found at sea is quite often under wet conditions or in subsurface layers, very close to the surface. Thus, resolving the real attenuation of reflectance associated with these situations is worth an additional research effort. In this regard future objectives could be:

- Improve our understanding of the BRDFs associated with dry polymers and compare them with results under wet conditions.
- Estimate more accurately the attenuation in reflectance caused by this effect and identify the most optimal viewing angle to minimize such attenuation.
- Potentially, check how aging of plastics alters their wetness degree and relate this with the attenuation effects that could be observed in the previous two points.
- Optimally, identify a potential empirical model to correct signature of wet plastics to compensate for such absorption, which could eventually form part of a forward model to invert plastic signature observed at TOA by the sensors.

Expanding the modelling capabilities

A set of aspects of the modelling should be also revisited to close the existing gap between the theoretical modelling presented in this paper and the capabilities of the current instruments. The main limitations of our modelling exercise could be addressed as follows:

- Determine with more accuracy the impact of different aerosols, which was done in a simplistic way. Only marine aerosols were considered in our exercise, whilst for coastal applications it would be of relevance to repeat some of the simulations with more complex aerosols.
- Properly address the mixing of polymers in the spectral signal, which was not done and could be explored with spectral unmixing methods. This aspect would be key to develop algorithms capable of differentiating polymers types and quantifying them.
- Provide a finer binning to the % pixel coverages addressed in this lab experiments, which was done only in orders of magnitude and might require a finer grid in the fractions below 10%. This is an important area for fine-tuning the limit of detectability.
- Better understand the impact of wind conditions over the ocean's surface in terms of sun glint and lighting conditions. In our modelling exercise, wind speed was fixed, but this could be revisited to identify till what point the sun glint could be a constraint.
- Check the signature of floating algae (*e.g.*, *Sargassum*), accumulations of marine phanerogam leaves, foams driftwood and other organic debris coming from land. From the current experimental setup, it could not be determined to what extent they can cause false positives. Modelled cases of floating ML aggregations (*e.g.*, LW) containing different matter mixes should be analysed, possibly trying to limit the number of cases to a statistical classification of ML aggregations in terms of content (versus geographical areas, sea state or season). Such a classification would need validation with in-situ surveys, particularly those covering the sampling of the so-called LWs.
- Investigate the impact of the uncertainties associated with the different parts of the model in order to understand their overall contribution to the results.

In addition to those elements, we identified a number of activities to expand the current model:

- Introduce an end-to-end simulator capable of transferring field measurements from artificial ML aggregations in order to simulate what they would look like with existing instruments (*e.g.*, S2-MSI, PRISMA, EnMap, Hyperion or VHR sensors). This could be based purely in simulated BOA information (based in spectral libraries and water-leaving simulations) instead of in field measurements, if those were not available.
- Similar to the above but for a theoretical instrument, which could support technological development before trying to physically breadboard. A good option could be to use the characteristics of the coming Copernicus Hyperspectral Imaging Mission (CHIME) satellite and to assess what this instrument could offer in the field

of ML. This could also be valid for other multispectral rather than hyperspectral instruments, which also has some advantages.
- Investigate the development of reflectance indices to detect, and potentially quantify and classify, the floating ML. Preliminary results based on an initial set of indices were already produced for the present work.

Based on the knowledge gained from our modelling exercises, we drew out a few overarching goals to support the further development of an EO4ML mission:

- Better understanding what are the errors and limitations of the models according to the existing knowledge. This will help to refine the applications of a potential EO4ML mission. Modelling, together with information from field and lab experiments, should aim to minimize the randomness of the choices done in the path to higher TRLs.
- Improved assessment of capabilities for monitoring ML from space from existing sensors. Here, we did extensive effort in the use of S2-MSI datasets. This effort could be expanded to existing hyperspectral missions and other coming multispectral missions. These assessments will help the engineering component to better identify what technological push might be needed.
- Refine the spectral lines and bands as well as the spectral indices for ML detection from space, including a better understanding of the potential false positives. This development would be particularly useful for prototyping the software that will be needed to pass from L1b/c TOA reflectance values to L2 data.

Of course, modelling efforts would also benefit greatly from in situ data. Validation of techniques and determination of uncertainties were some of the most difficult aspects we faced in this work. The deployment of artificial targets to be observed by remote sensing platforms, from drones to satellites to airplanes, is a worthwhile way to advance these tasks. Similarly, there is a strong need for more oceanographic surveys in coordination with remote sensing platforms.

Known caveats

The present work essentially addressed spectral methods, with emphasis in multispectral sensors, identified in our initial analysis as having the greatest potential for ML remote sensing. Alternative remote sensing technologies, *e.g.* active techniques such as SAR and LiDAR, while addressed in this study, were not evaluated in detail. Our area of interest was the global ocean surface, from nearshore to offshore waters. We did not confirm applicability for beaches, due to the complex background in these areas. Probably, other approaches such as (sensors on drones can offer better chances in beaches, as even VHR satellites have shown limited capabilities over these areas[69]. Beaches are one of the regions of greatest interest, due to the ML impact on activities such as tourism as well as on the natural ecosystems.

Regarding the observable range of ML size, we concluded that the best opportunity relies on the identification of sub-mesoscale aggregations of floating plastic items. The observable size of these aggregations will be limited by the SNR that can be afforded and the size of the pixel. S2-MSI may offer current limited capabilities for its observation. We still know little about the relative contribution of microplastics and macroplastics to the LWs, however, given the relevance of macroplastic items in terms of plastic coverage of the ocean surface[15,17], macroplastic fraction could probably also be the largest contributor to these aggregations.

S4. Supplementary Discussion for Proof of Concept

The case of the North Adriatic

The North Adriatic presented the highest LWD in the Mediterranean Sea. This is a confined basin surrounded by areas with large populations and the rainiest watersheds, drained by a low-dammed river system[70]. Accordingly, extensive ship-based surveys reported maximum concentrations of floating macro-plastic items in the Adriatic Sea[71,72]. Satellite observations delimited the innermost section, adjacent to the mouth of the Po and other rivers draining the Alps, as having the largest LWD. As a result, while Mediterranean lagoons appeared as water bodies prone to litter accumulation, the enclosure of the Venice lagoon (northwestern Adriatic shore) acted as protection against the influx of ML from the Adriatic Sea (Fig. S33b).

The distribution of floating ML is expected to be shaped by pollution sources and surface transport. This interplay was nicely illustrated with the LW tracking in the Adriatic Sea. Long-term LW detections showed how the Western Adriatic Current (WAC) spread floating litter from the Po southwards alongside the Italian coast (Fig. 3 and 5). Yet, this LWs field varied significantly over time (Fig. S26). LWD peaked twice, in spring and summer, diverging from the rest of the Mediterranean (Fig. 8b and Fig. S32). The particular LWD maximum in the dry summer could be explained by the snowmelt in the alpine watersheds, which leads to a singular summer peak of river discharges in the northern Adriatic[73]. Compared to spring maximum, summer distribution of LWs remained more retracted in the north, coinciding with a WAC weakening[74]. Winds generate powerful surface currents in the Adriatic[75], leaving also their imprint on the LWs field. Strikingly, North Adriatic surface waters were systematically cleared every year during the wind speed peaks in December and January (Fig. S26).

Exploring possible applications with the S2-MSI sensor

LW detections in the Mediterranean Sea were mainly related to recent inputs of litter from land-based sources. The main limitation of the current detector is probably its low sensitivity, we need to process many images to get a few detections, especially if the study site is small. But even so, the possibility of sustained LW monitoring from space opens up great prospects for application.

In this section we explore possible applications in real cases. In the **Bay of Biscay**, for instance, a fishing vessel devoted to LW clean-up from May to August 2018[12]. It was able to recover 240 kg of litter per day with no external guidance to search LWs. Based on 6 years of sustained monitoring with S2-MSI in this Atlantic region, we found that LWD usually peaks in April and October, and realized that the area just north of the clean-up area presented the highest LW abundance (4 fold higher than in the habitual clean-up area).

Remote sensing also allows pinpointing the most potentially littered sites, sometimes overlooked by ship-based assessments. Mediterranean lagoons as well as other semi-enclosed water bodies often showed abundant presence of LWs. This was the case, for example, of **Mar Menor** in Spain or **Bardawil Lagoon** in Egypt (Fig. S33a and Fig. S33d). A case in point is the **Ambracian Gulf** in Greece, noted for hosting a critical marine mammal population[76] and flagged here as having one of the highest LWD in the Mediterranean (LWD_{mga} = 30.7 ppm, Fig. S33c). A singular finding was the accumulation of LW next to the ship waiting area to cross the **Suez Canal**, which emerges as a potentially relevant source of floating litter (Fig. S33d).

A further application can be illustrated with the **Tiber River**. Following its transit across Rome, the regional government installed floating barriers in October 2019, trapping more than 6 tons of litter in 18 months of activity[77]. However, the barriers failed to prevent the largest LW pulse detected in waters off the Tiber delta during the rainy March 2021. LWD was 30.1 ppm in this month, which represents half of all LW detections over the 6-year observation period. The exceptional amount of plastic litter flushed in March 2021 was featured by local news[78]. Excluding this particular event, LWD off the Tiber during the period with barriers was 38% lower than in the period without, suggesting a significant effect of the barriers under low and moderate river discharges. As a reference, we can use the giant bar screen installed across the Huveaune River in Marseille (France)[79]. This rigid screen was also ineffective in periods of heavy rainfall, but it prevented 65% of the waste flow to reach the sea (including periods of heavy rainfall in the account).

SUPPLEMENTARY FIGURES

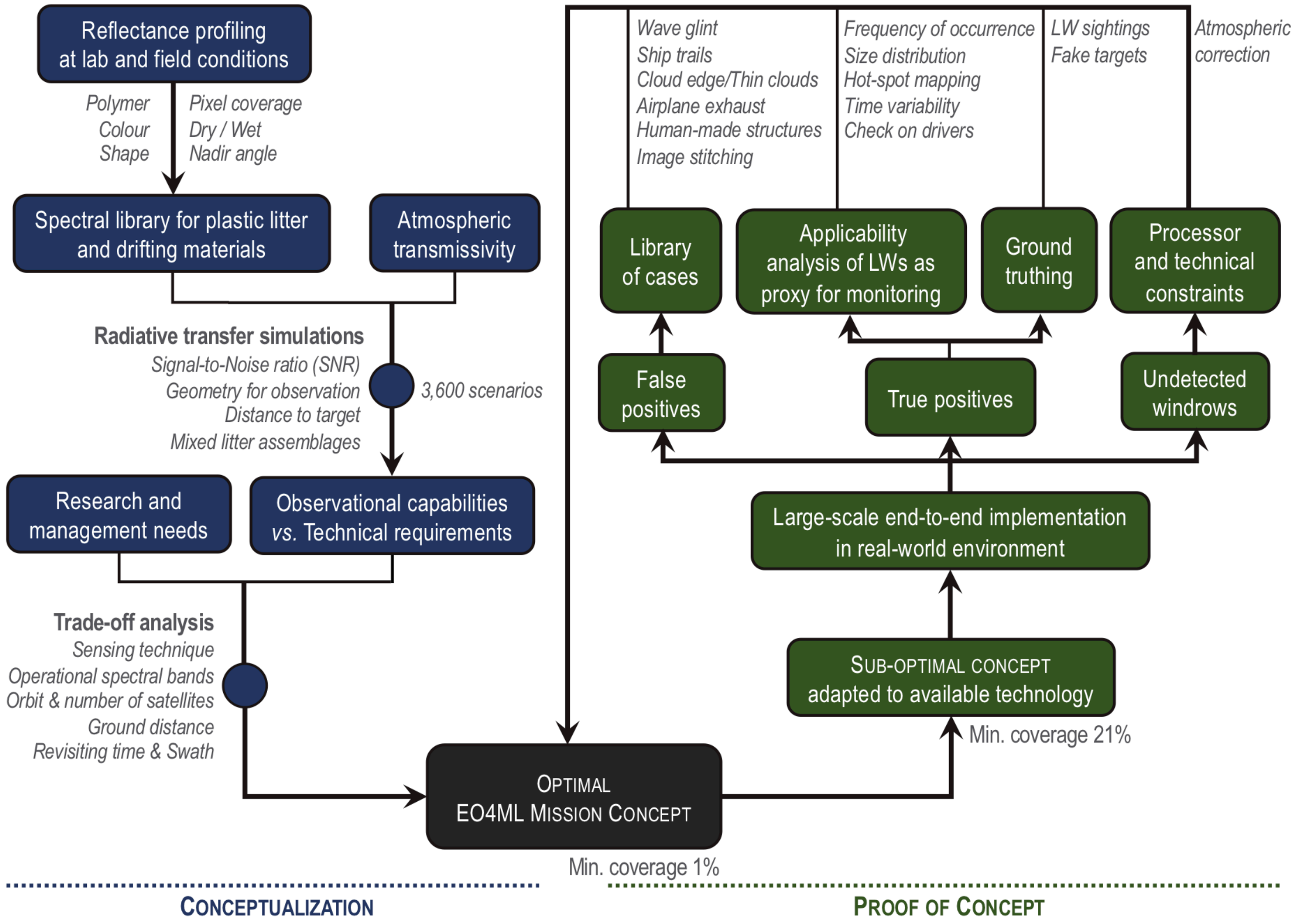

Fig. S1 | Roadmap followed in the present study for the definition of the optimal EO4ML mission concept and the proof of concept. The minimum observable coverage estimated for the optimal mission concept and the proof of concept area are expressed as fraction of surface cover into the pixel.

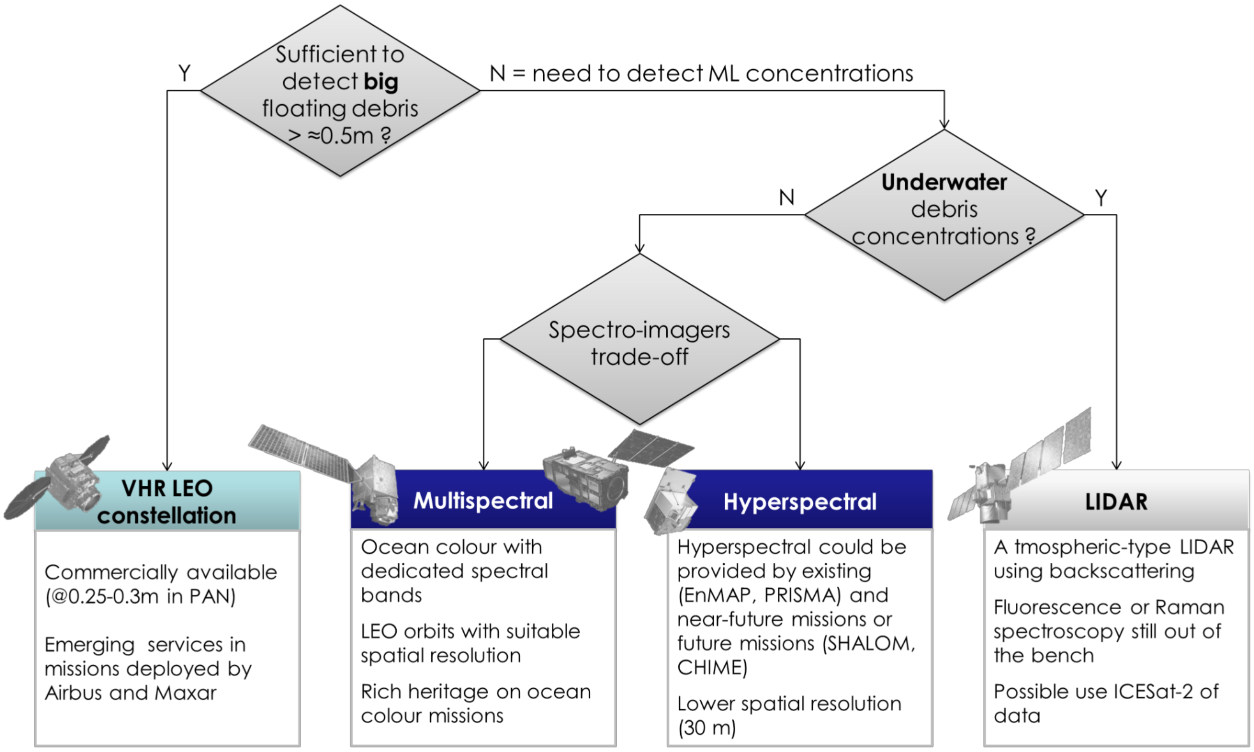

Fig. S2 | General decision tree for the sensor type according to mission requirements. Satellite images given for illustration only.

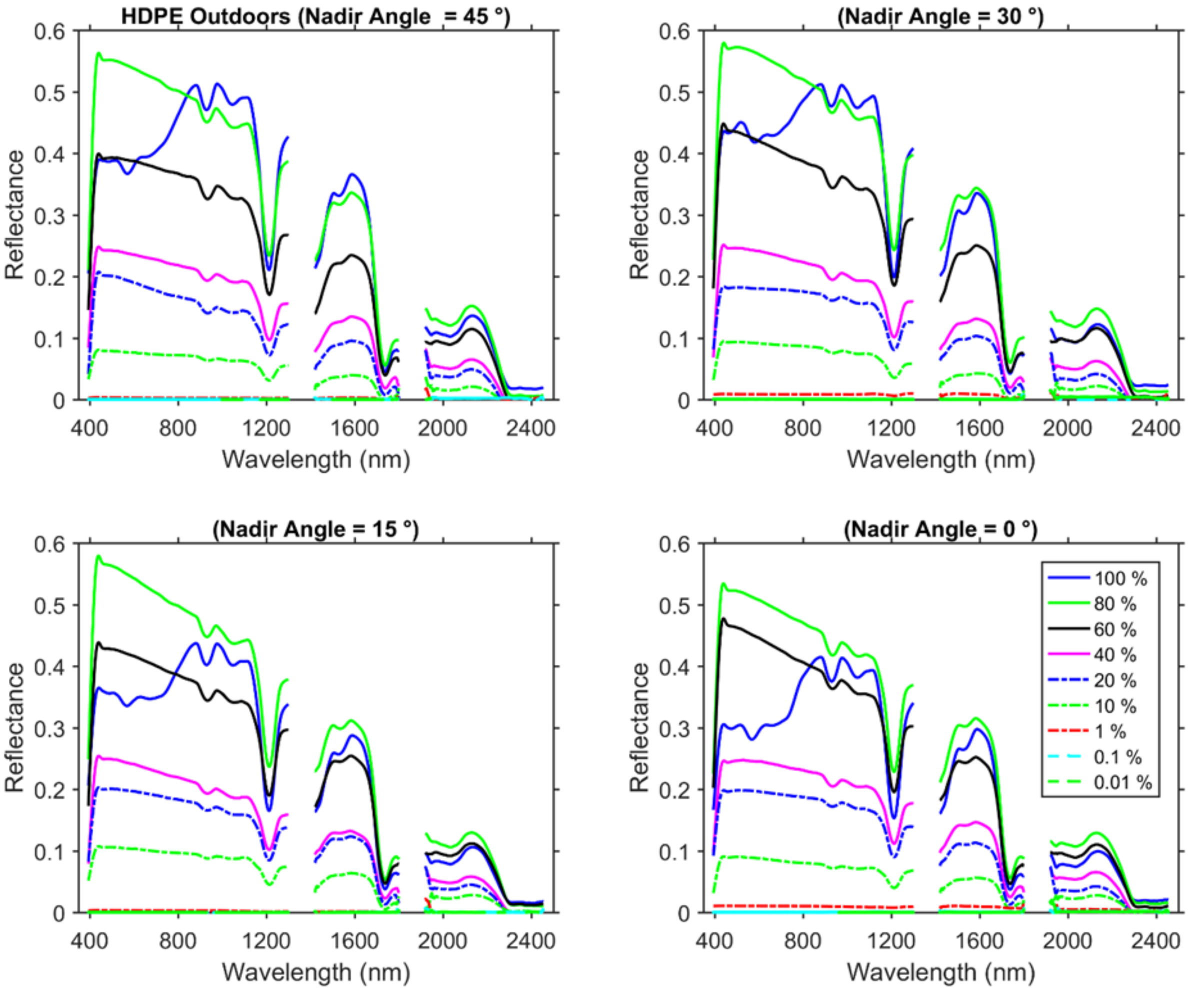

Fig. S3 | Example of reflectance spectra for dry high-density polyethylene (HDPE). Note we accounted for varying nadir viewing angles and HDPE coverages (in percentage of pixel coverage, see legend).

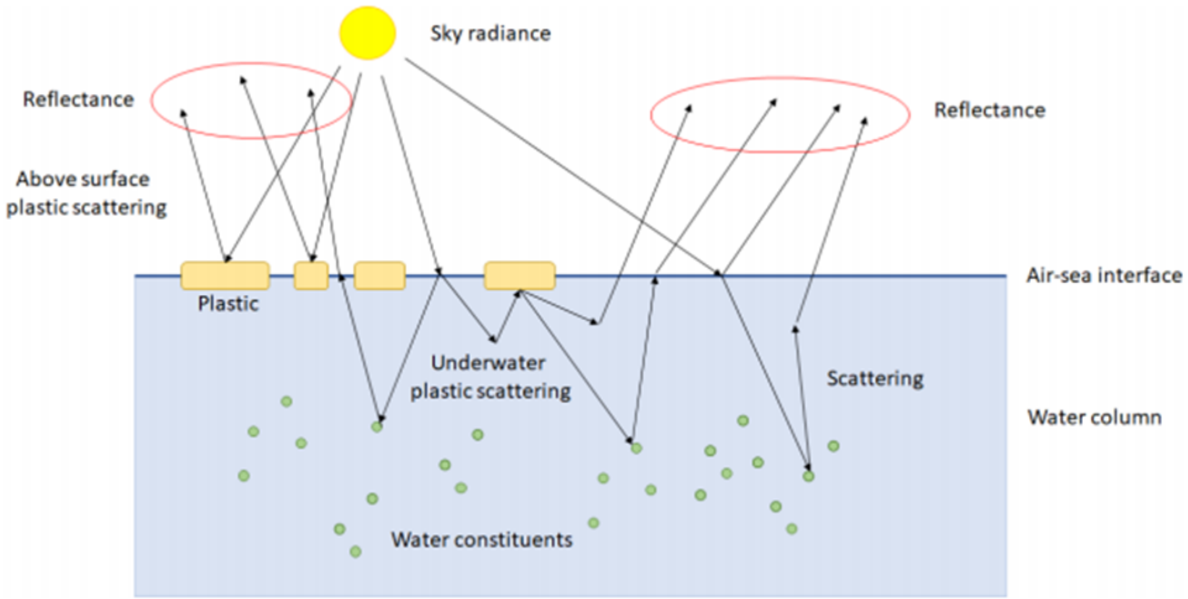

Fig. S4 | Behaviour of the photons interacting with a seawater mass of infinite depth and a fraction of its surface covered by plastic debris.

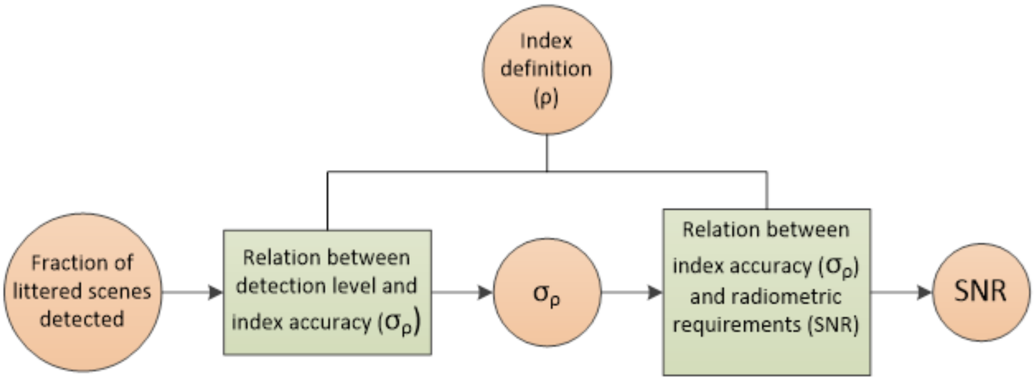

Fig. S5 | General approach followed to derive radiometric requirements for target spectral bands.

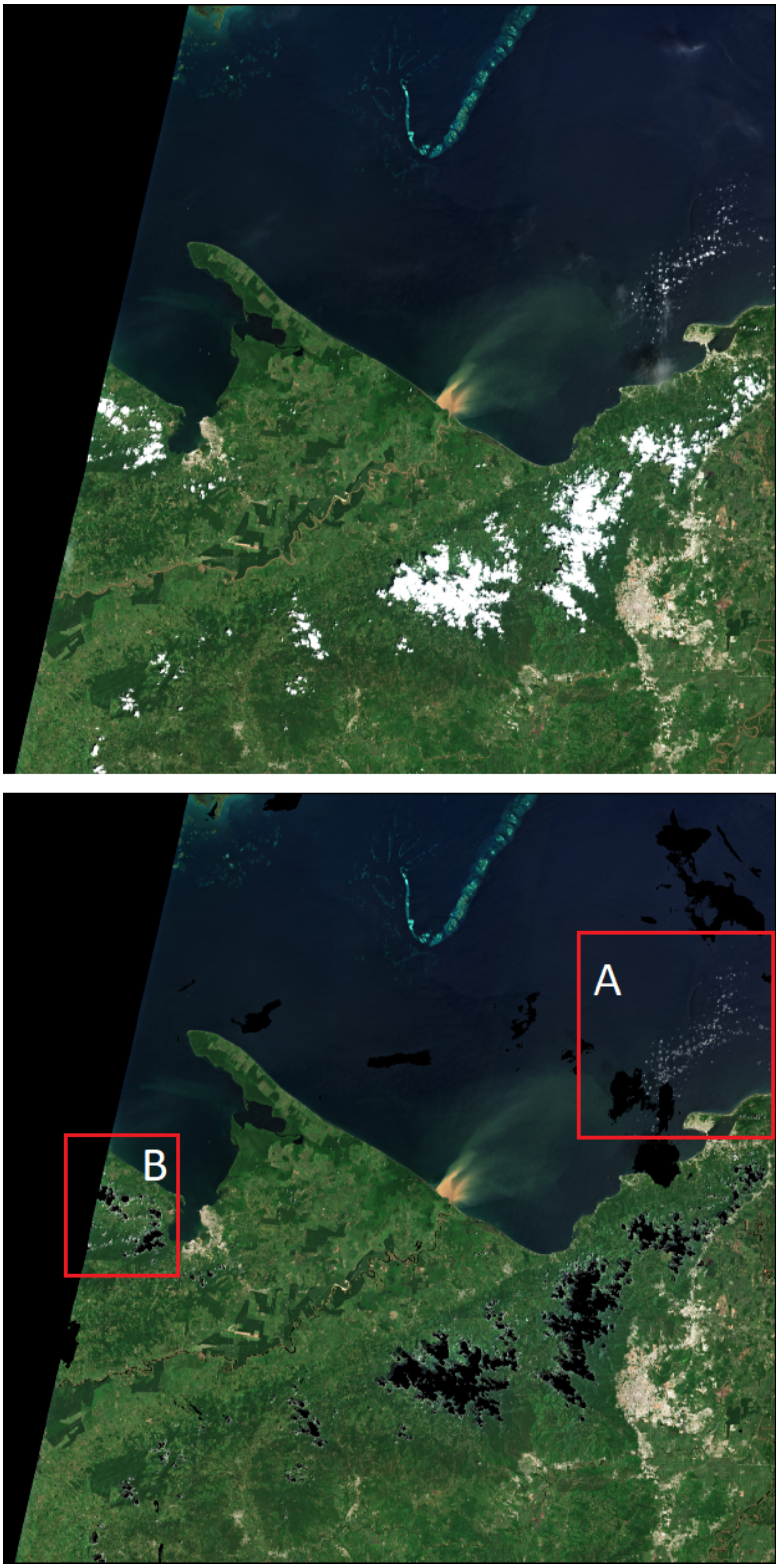

Fig. S6 | Example of performance issues for Sen2Cor cloud masking. Top, Raw True Colour image (generated using B04, B03 and B02, for RGB, respectively). **Bottom**, Same image with SCL cloud masking (values from 7 to 10 marked in black). Red boxes frame relevant issues, including undetected small clouds (box A) and the “halo” issue (box B). Special attention was paid to halos as they can lead to false positives.

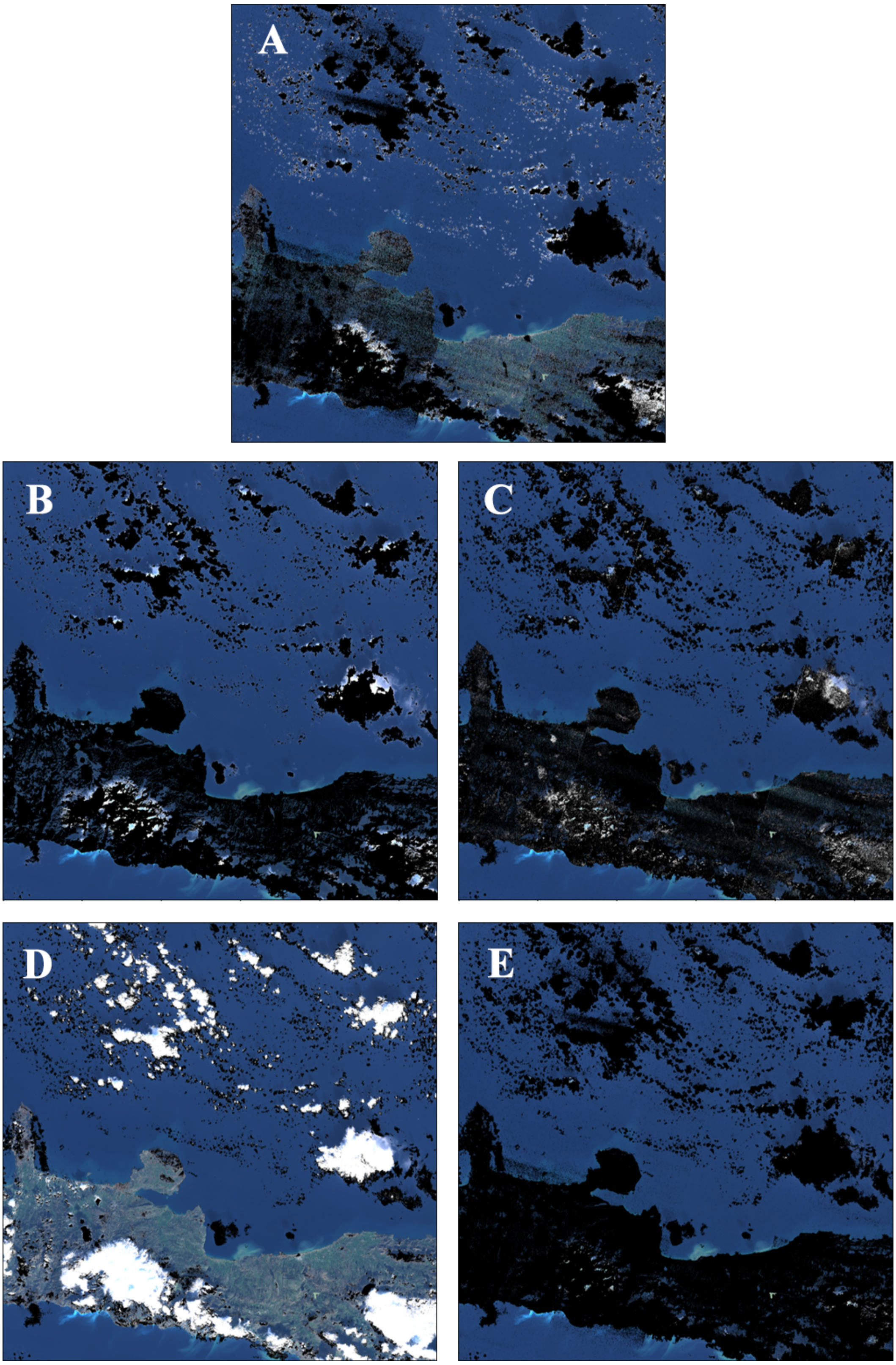

Fig. S7 | Cloud screening strategy. The example S2-MSI image is from Crete (Greece), Sentinel-2 tile 35SKV, 21 Jan 2019. Black pixels are the masked areas by the PoC processor cloud masking, while white pixels indicate potential inaccuracies in different masks. **a**, Sen2Cor cirrus cloud masking. **b**, MEETC2 masking alone. **c** CDI masking alone. **d**, LaSRC masking alone. **e**, Combined masks (A-D) used in the PoC.

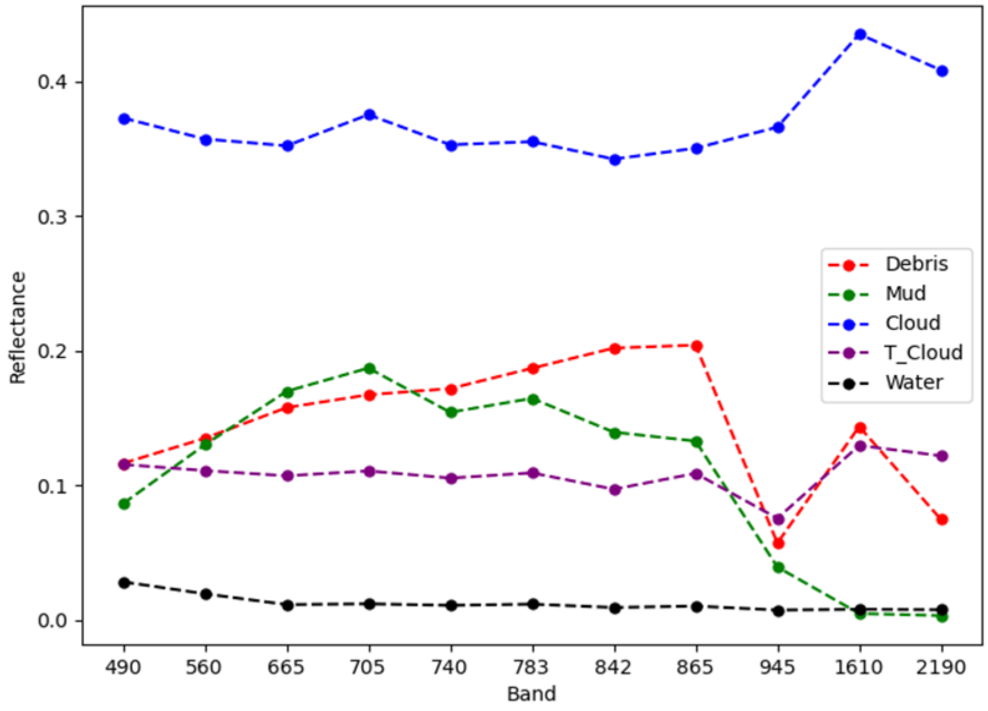

Fig. S8 | S2-MSI reflectance spectra for different pixel classes obtained from MARIDA database[7], i.e. floating marine debris, waters with high sediment load, clouds, thin clouds, seawater. S2-MSI B10 is omitted since it is for cirrus detection (section S2.1.2).

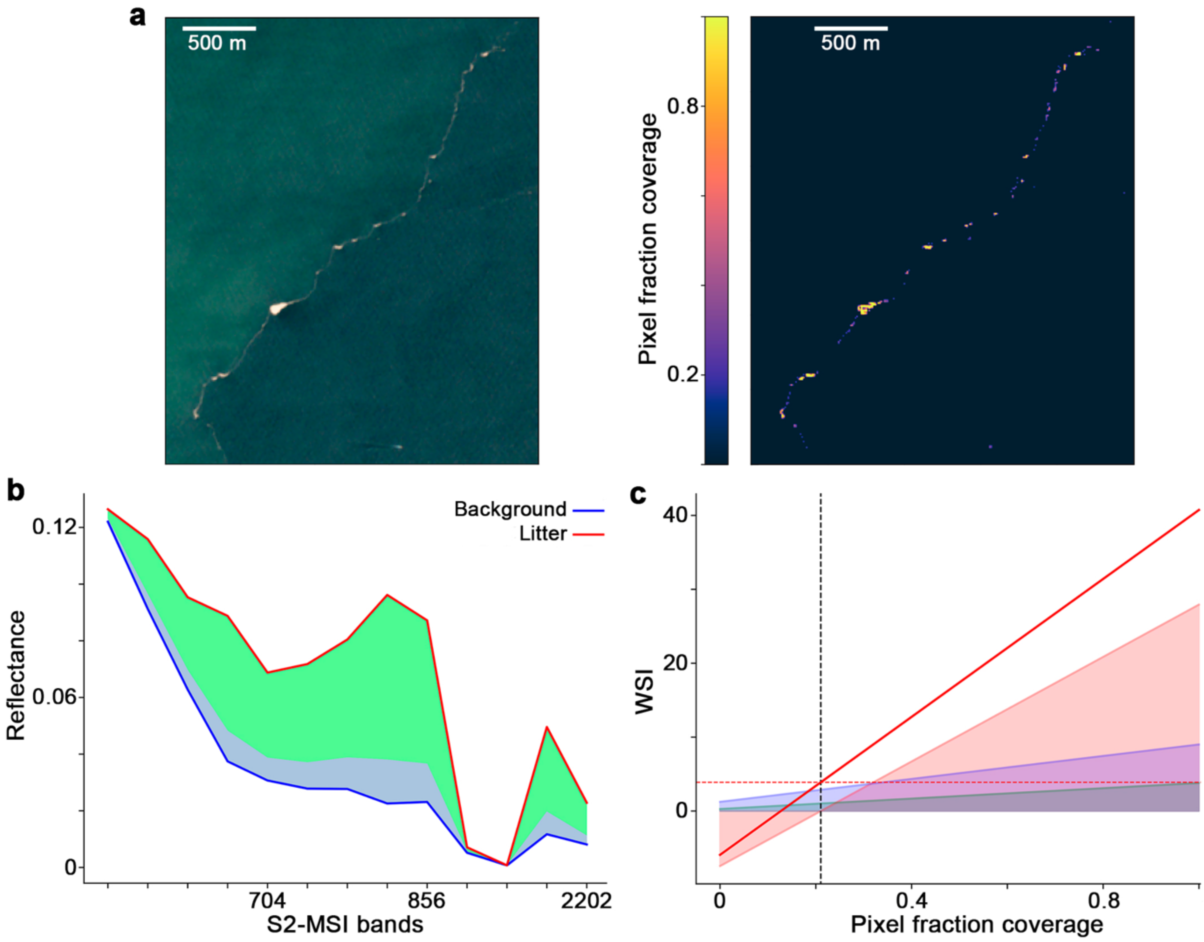

Fig. S9 | Analysis of the PoC spectral index (WSI) on a real LW. a, True-colour snippet of a S2-MSI acquisition showing a LW in Omoa Bay (Honduras, 19 Sept 2020) reported to accumulate large amounts of plastic debris[7] (left); and estimated fraction of the pixel covered by the LW (right). **b**, Mean spectral profile extracted from S2-MSI L1c data (TOA) for a set of pure LW pixels and pure background pixels. Green and grey areas show the range of profiles for which the WSI yields positive (green) and negative (grey) detection. **c**, Relationship between LW fraction coverage in the pixel and WSI (red line). Shaded red, green and blue areas correspond to the wsi_a, wsi_b, and wsi_c components of WSI, respectively (see section S2.1.7). Dashed lines mark the minimum coverage above which the PoC processor detects presence (21%), a threshold coincident with the estimate derived from experiments with artificial plastic targets (20%[5]).

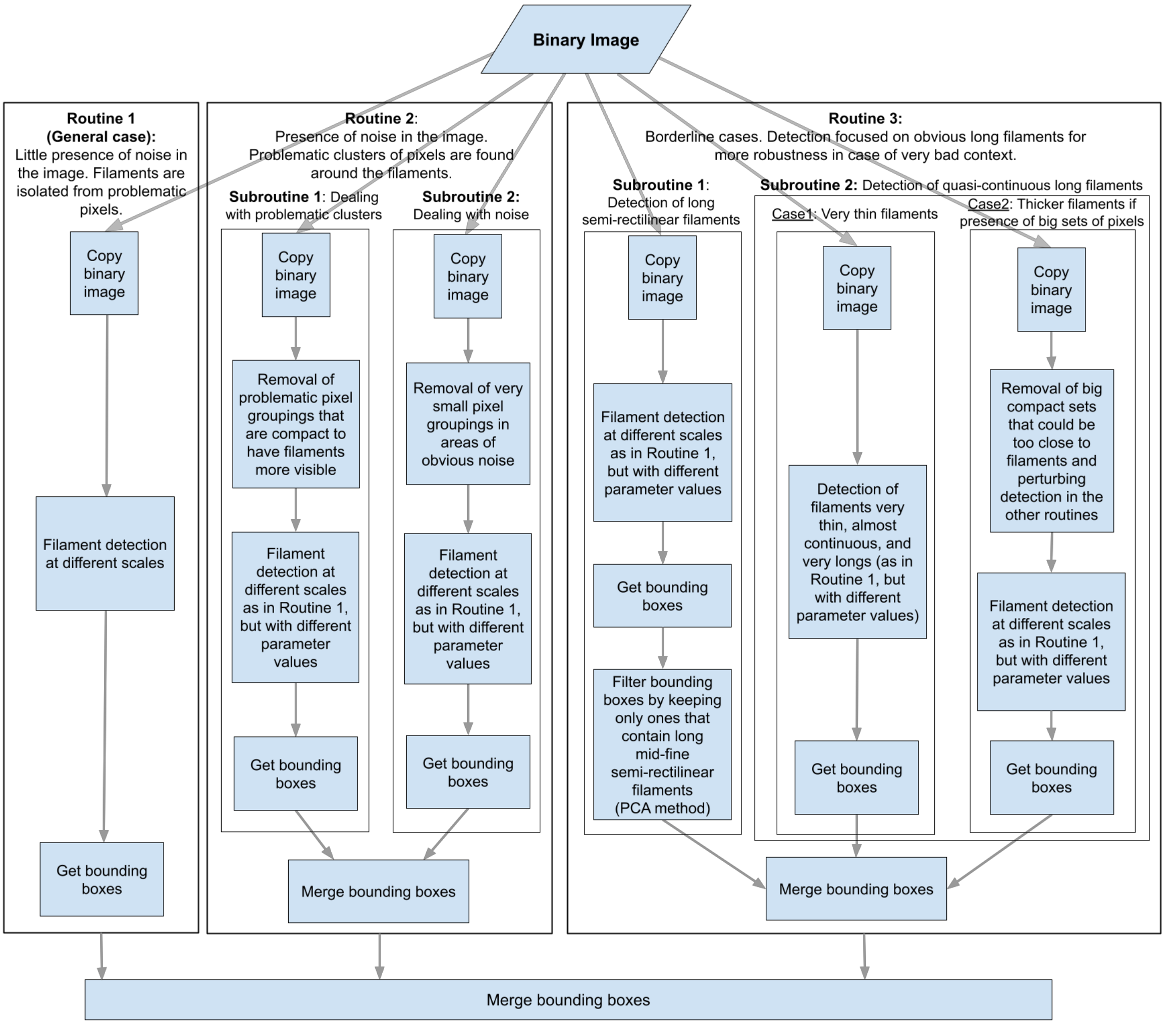

Fig. S10 | Workflow for the contextual filament classifier.

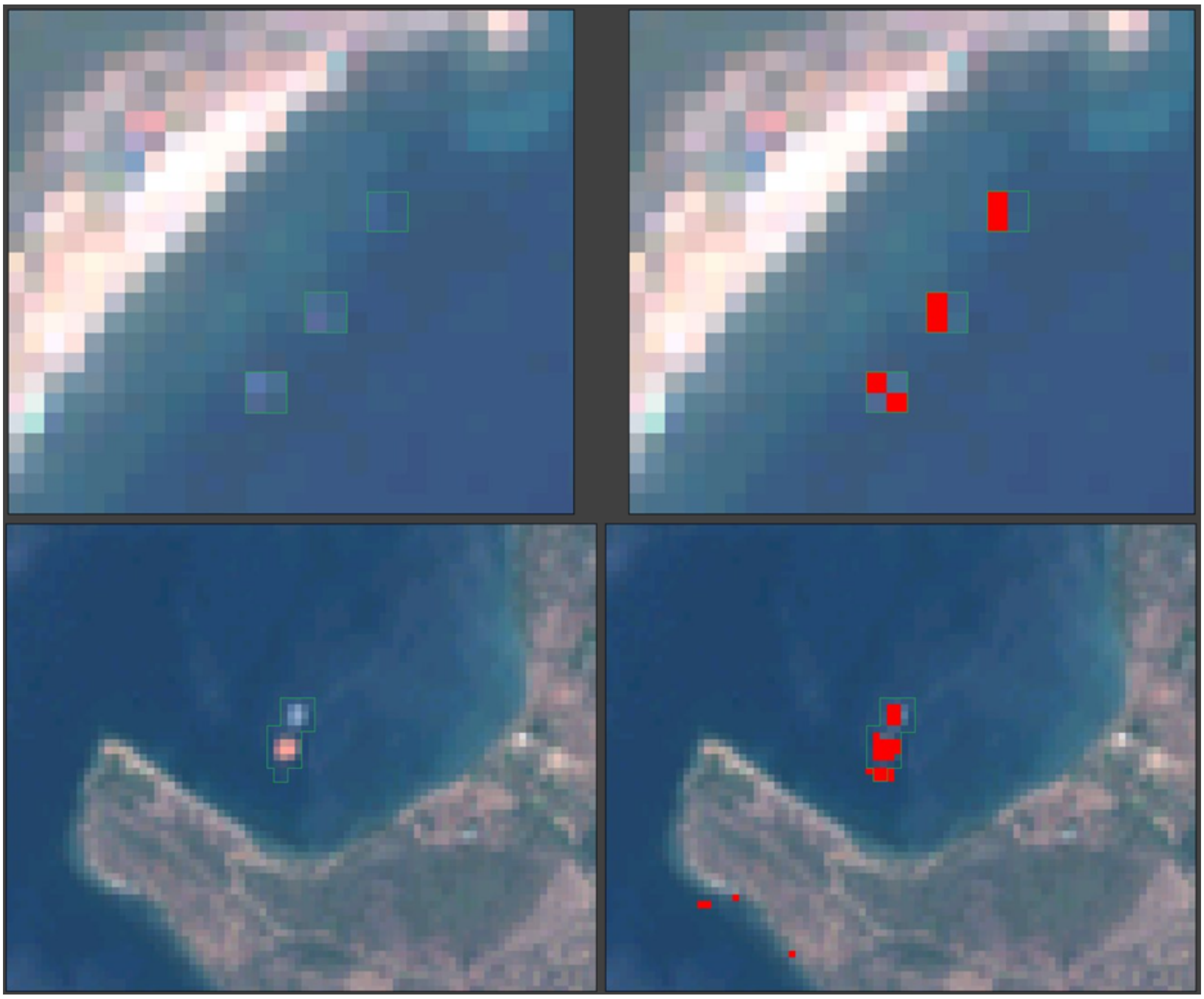

Fig. S11 | Detection performance of the PoC spectral index (WSI) on artificial ML targets. PLP-2018 experiment[6] (upper row) and PLP-2021 experiment[5] (lower row). True-colour S2-MSI images showing artificial ML targets (left panels), and positive pixels identified by the WSI, in red (right panels).

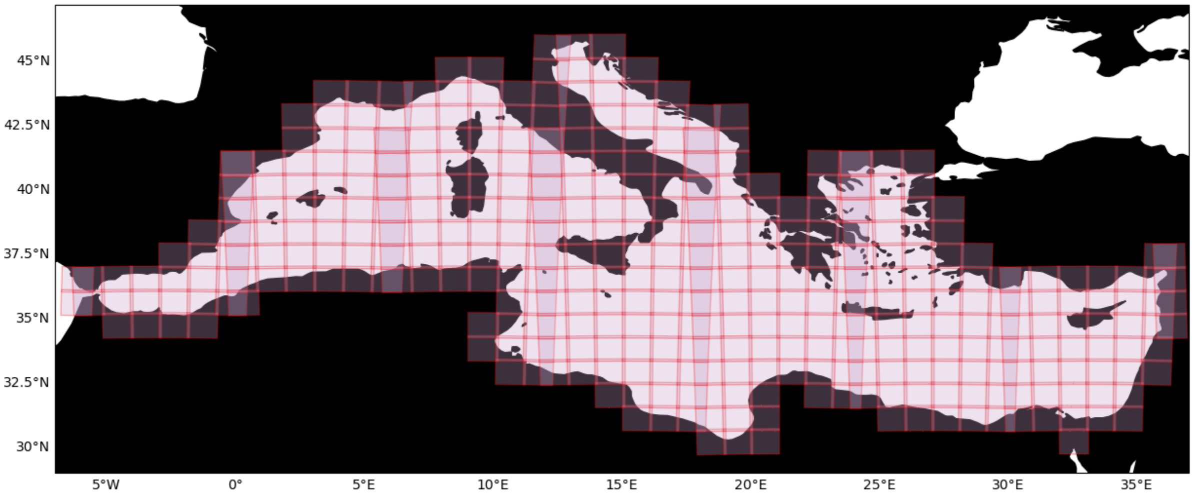

Fig. S12 | Positions of the Sentinel-2 tiles processed for the PoC over the Mediterranean Sea. A total of 288,166 S2-MSI L1c images (150 TB of data) corresponding to 411 tiles over the Mediterranean Sea were processed, covering the period from 4 July 2015 to 21 September 2021.

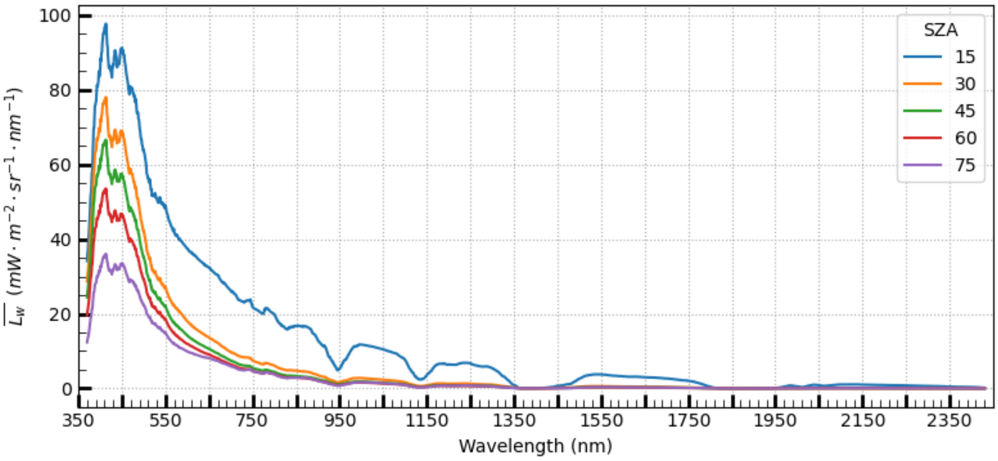

Fig. S13 | Spectral averaged radiance at TOA obtained for one of the reference scenarios (no plastic, *Chl-a* concentration = 0.01 mg m^{-3}, wind speed = 1 m s^{-1}). Different SZA are shown, namely 15°, 30°, 45°, 60° and 75°.

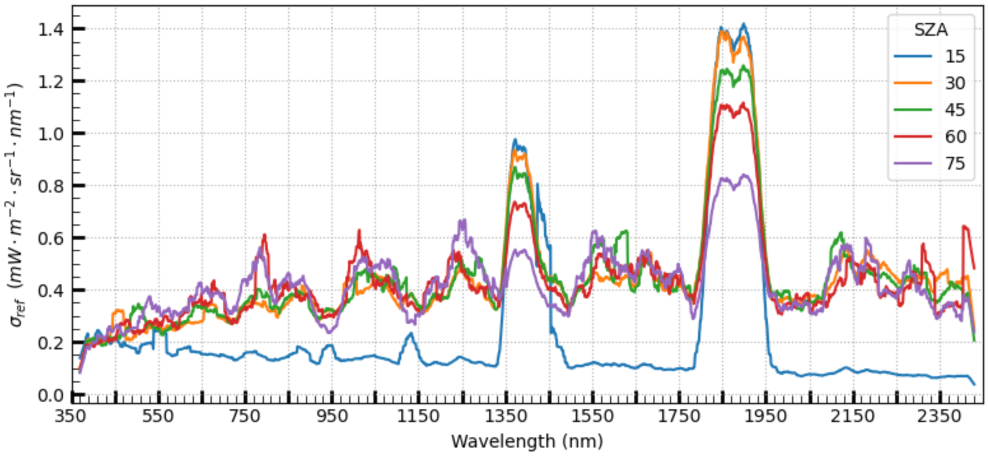

Fig. S14 | Simulated radiance uncertainties at TOA obtained for one of the reference scenarios (no plastic, *Chl-a* concentration = 0.01 mg m^{-3}, wind speed = 1 m s^{-1}). SZA is set at 15°, 30°, 45°, 60° and 75°.

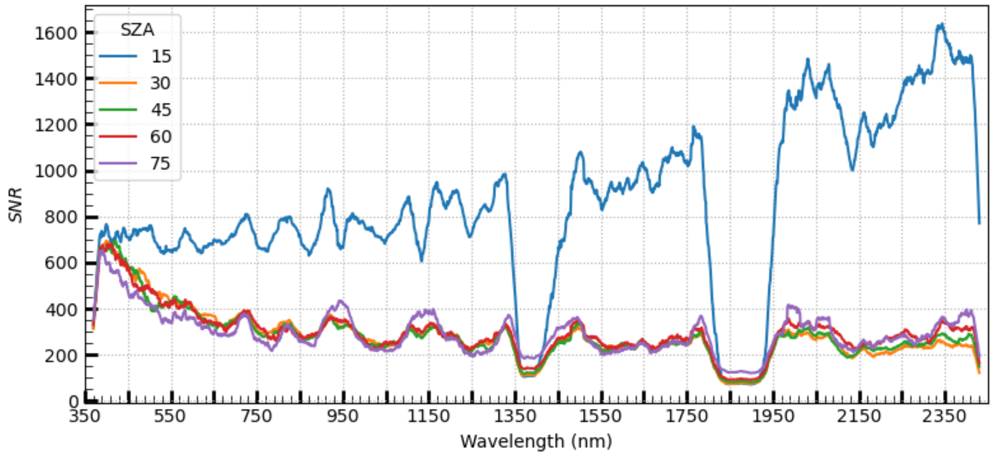

Fig. S15 | SNR at TOA obtained for a reference scenario (no plastic, *Chl-a* concentration = 0.01 mg m^{-3}, wind speed = 1 m s^{-1}). SZA is set at 15°, 30°, 45°, 60° and 75°, radiances correspond to those shown at Fig. S13 and the photon noise is yielded by MYSTIC at each wavelength.

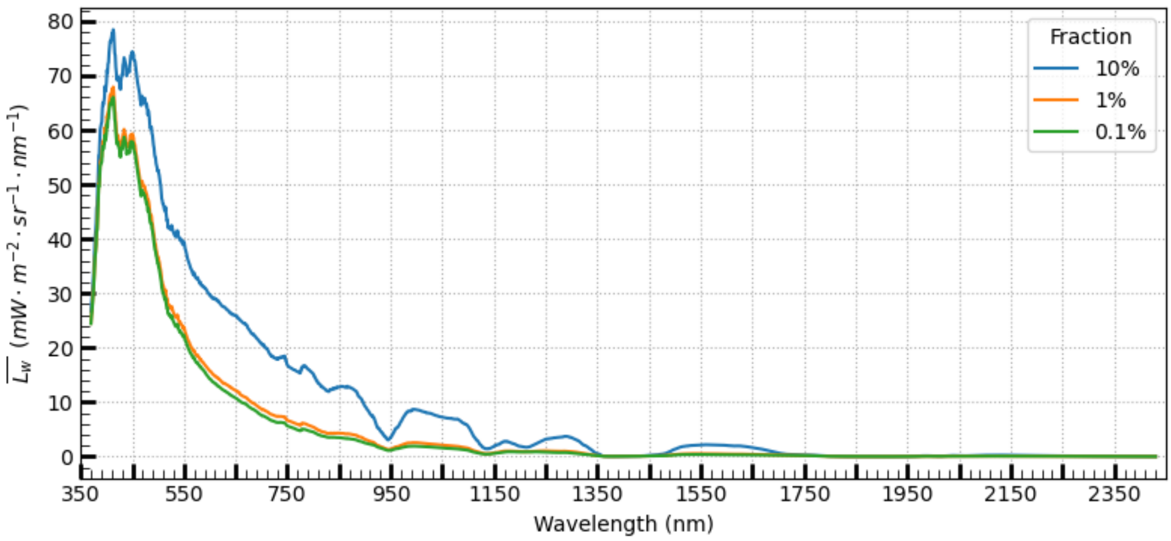

Fig. S16 | Spectral averaged radiance Lw for low-density polyethylene (LDPE) polymer in a given observation scenario (SZA = 45°, *Chl-a* concentration = 0.01 mg m^{-3}, wind speed = 1 m s^{-1}). The fraction of pixel covered by LDPE is set at 10%, 1%, and 0.1%.

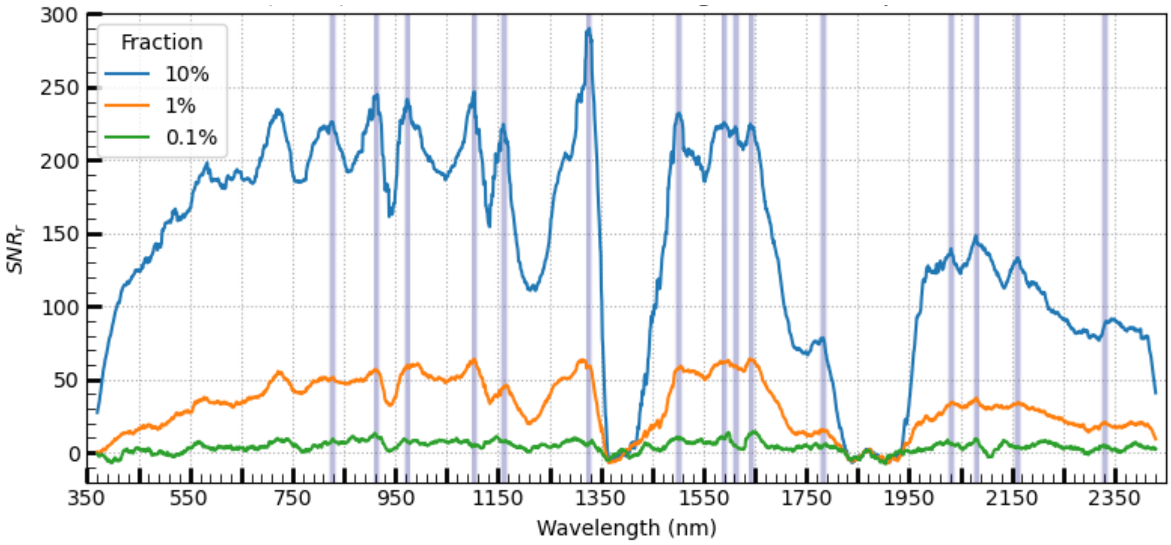

Fig. S17 | SNRr spectra for low-density polyethylene (LDPE) polymer in a given observation scenario (SZA = 45°, *Chl-a* concentration = 0.01 mg m^{-3}, WS = 1 m s^{-1}). The fraction of pixel covered by LDPE is set at 10%, 1% and 0.1% (see legend). Gray vertical lines represent local maximums that identify NIR-SWIR bands useful for detection of floating LDPE based on spectral anomalies.

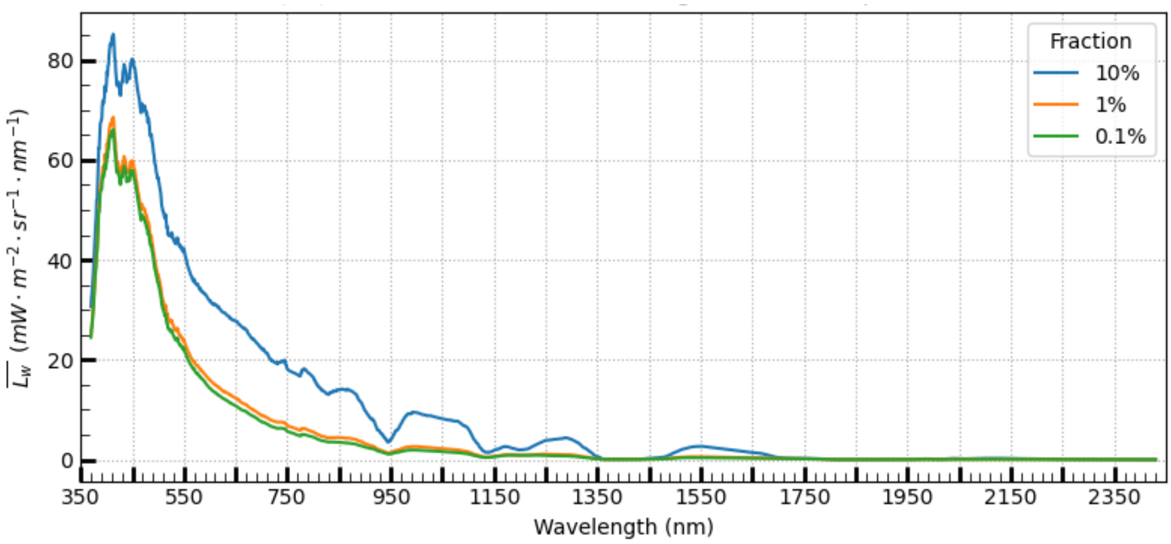

Fig. S18 | Spectral averaged radiance Lw for polypropylene (PP) polymer in a given observation scenario (SZA = 45°, *Chl-a* concentration = 0.01 mg m^{-3}, WS = 1 m s^{-1}). The fraction of pixel covered by PP is set at 10%, 1%, and 0.1% (see legend).

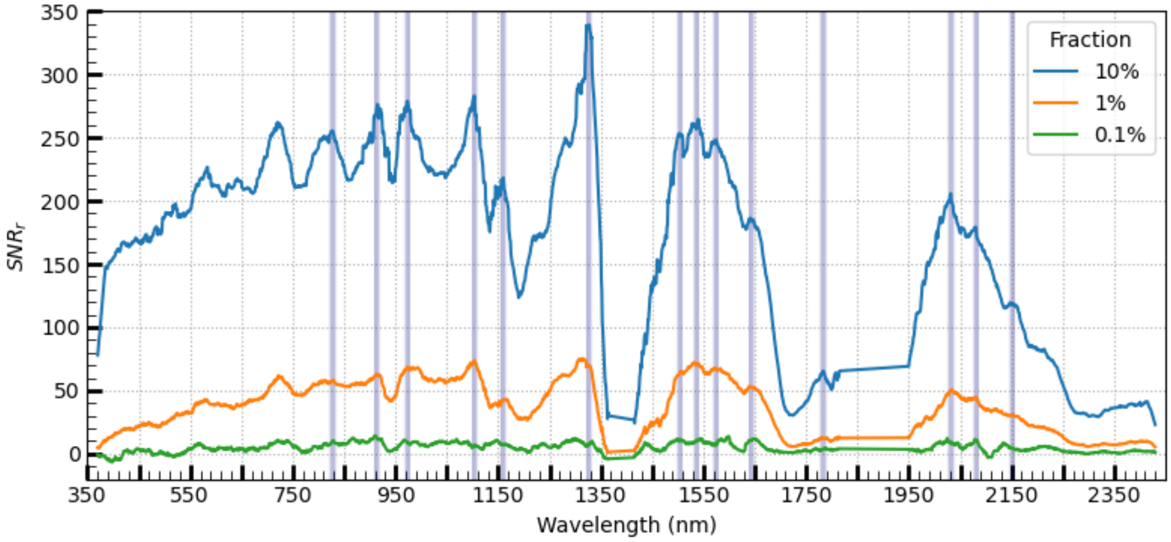

Fig. S19 | SNRr spectra for polypropylene (PP) polymer in a given observation scenario (SZA = 45°, *Chl-a* concentration = 0.01 mg m^{-3}, WS = 1 m s^{-1}). The fraction of pixel covered by PP is set at 10%, 1% and 0.1% of pixel coverage (see legend). Gray vertical lines represent local maximums that identify bands useful for detection of floating PP based on spectral anomalies.

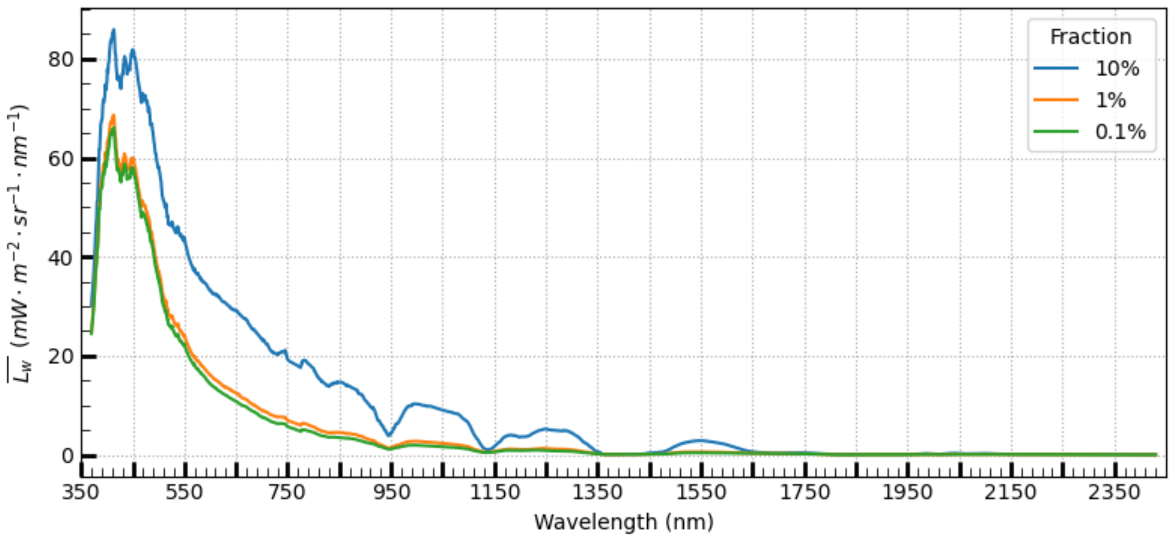

Fig. S20 | Spectral averaged radiance Lw for polystyrene (PS) polymer in a given observation scenario (SZA = 45°, *Chl-a* concentration = 0.01 mg m^{-3}, WS = 1 m s^{-1}). The fraction of pixel covered by PS is set at 10%, 1%, and 0.1% (see legend).

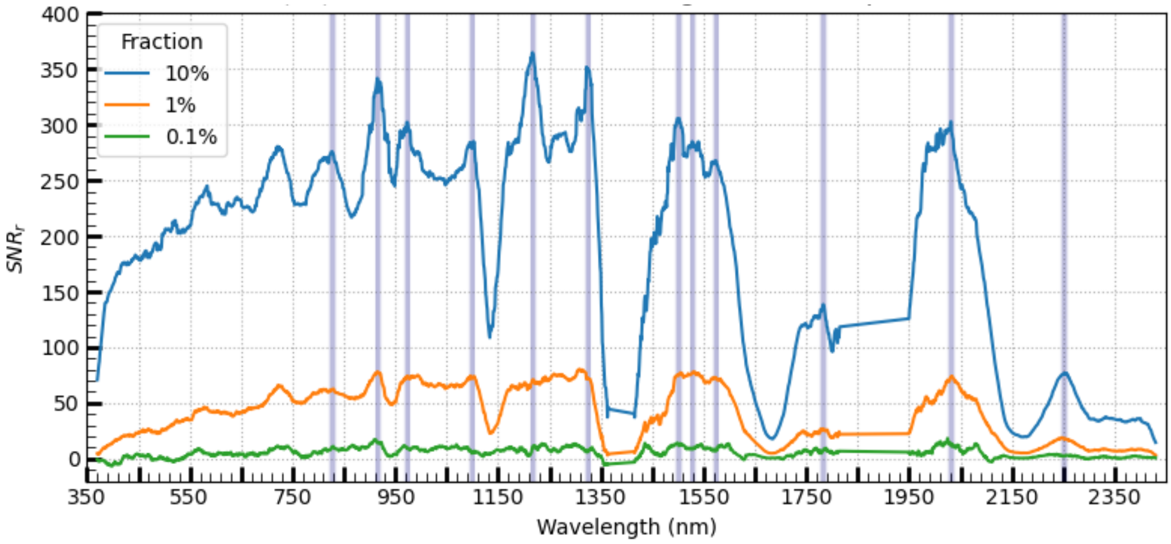

Fig. S21 | SNRr spectra for polystyrene in a given observation scenario (SZA = 45°, *Chl-a* concentration = 0.01 mg m^{-3}, WS = 1 m s^{-1}). The fraction of pixel covered by PS is set at 10%, 1% and 0.1% (see legend). Gray vertical lines represent local maximums that identify bands useful for detection of floating PS based on spectral anomalies.

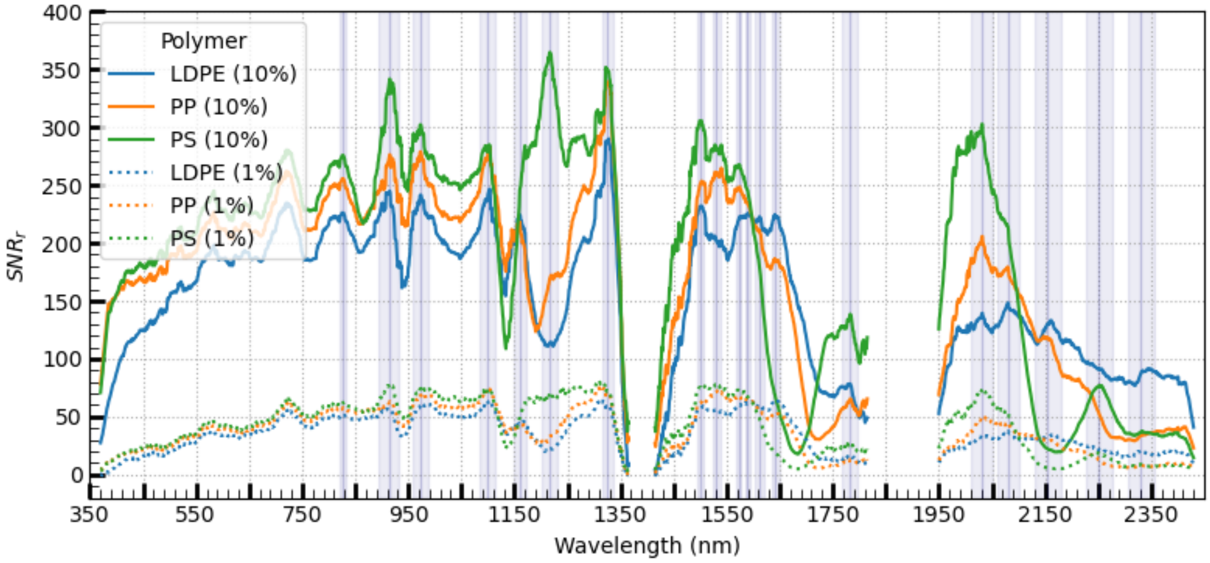

Fig. S22 | Candidate narrow bands for the fine solution in a given observation scenario (SZA = 45°, *Chl-a* concentration = 0.01 mg m^{-3}, WS = 1 m s^{-1}). A total of 19 bands are shown as grey vertical lines. SNRr for SZA=45° for 10%- and 1%-pixel coverage are included for reference. Gaps are associated with the atmospheric absorption windows, which have been discarded.

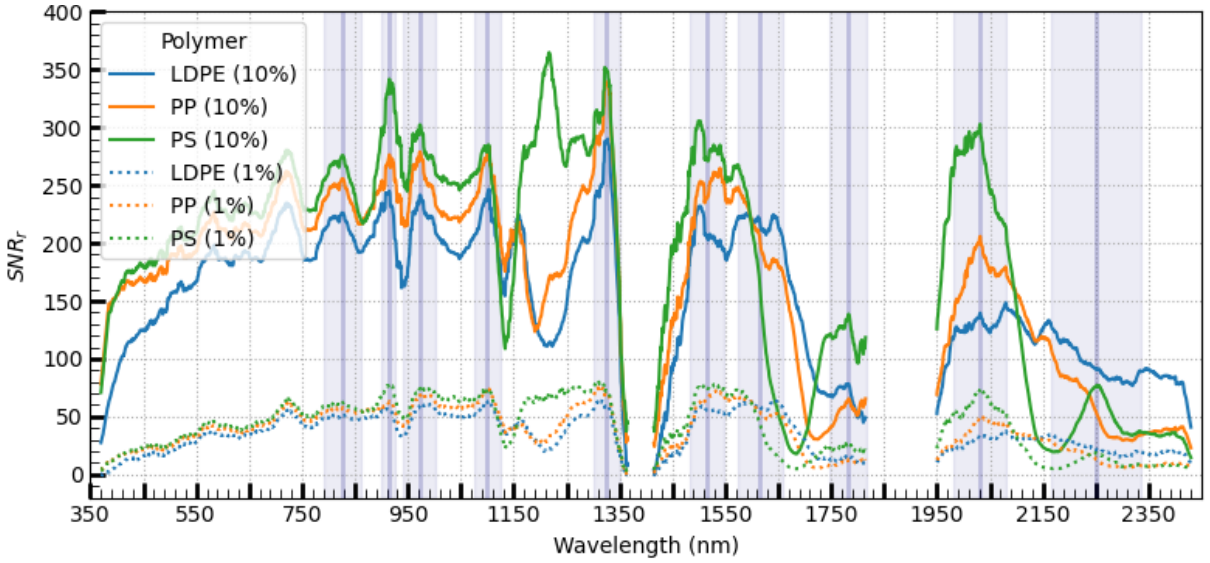

Fig. S23 | Candidate wide bands for the coarse solution in a given observation scenario (SZA = 45°, *Chl-a* concentration = 0.01 mg m^{-3}, WS = 1 m s^{-1}). A total of 10 bands are shown as grey vertical lines. SNRr for SZA=45° for 10%- and 1%-pixel coverage are included for reference. Gaps are associated with the atmospheric absorption windows, which have been discarded.

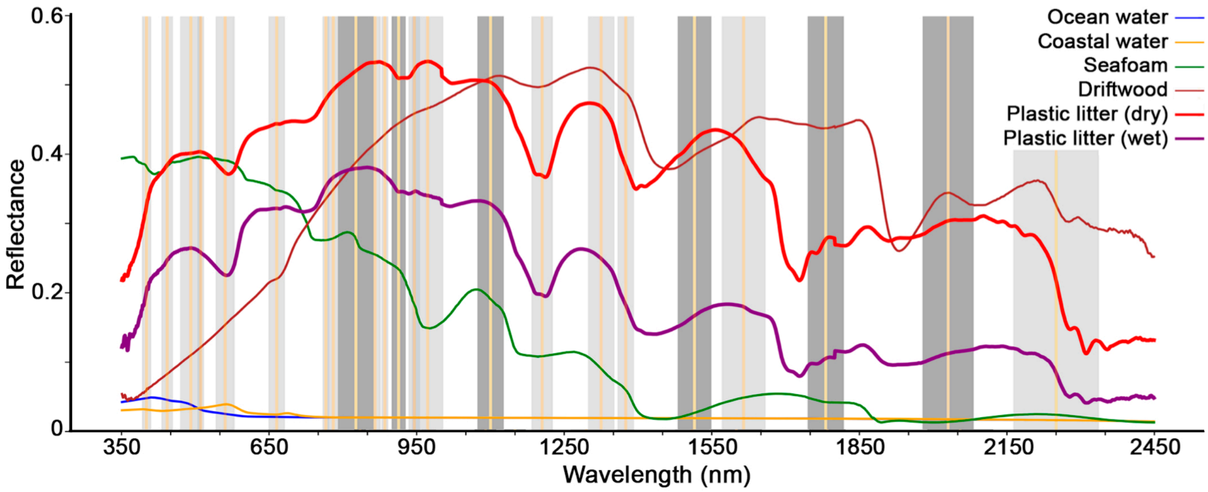

Fig. S24 | Spectral reflectance for plastic and other major components of floating litter, together with the final selection of candidate bands for the EO4ML mission. Optimal bandwidths and band-centres for the EO4ML mission are shown with grey bands and yellow lines, respectively. Dark grey indicates the minimum set of bands needed to achieve a detection threshold of 1%. Light-grey bands could improve plastic detection and spectral unmixing of LW components, as well as atmospheric correction and cloud detection (functionality per band is described in Table S23).

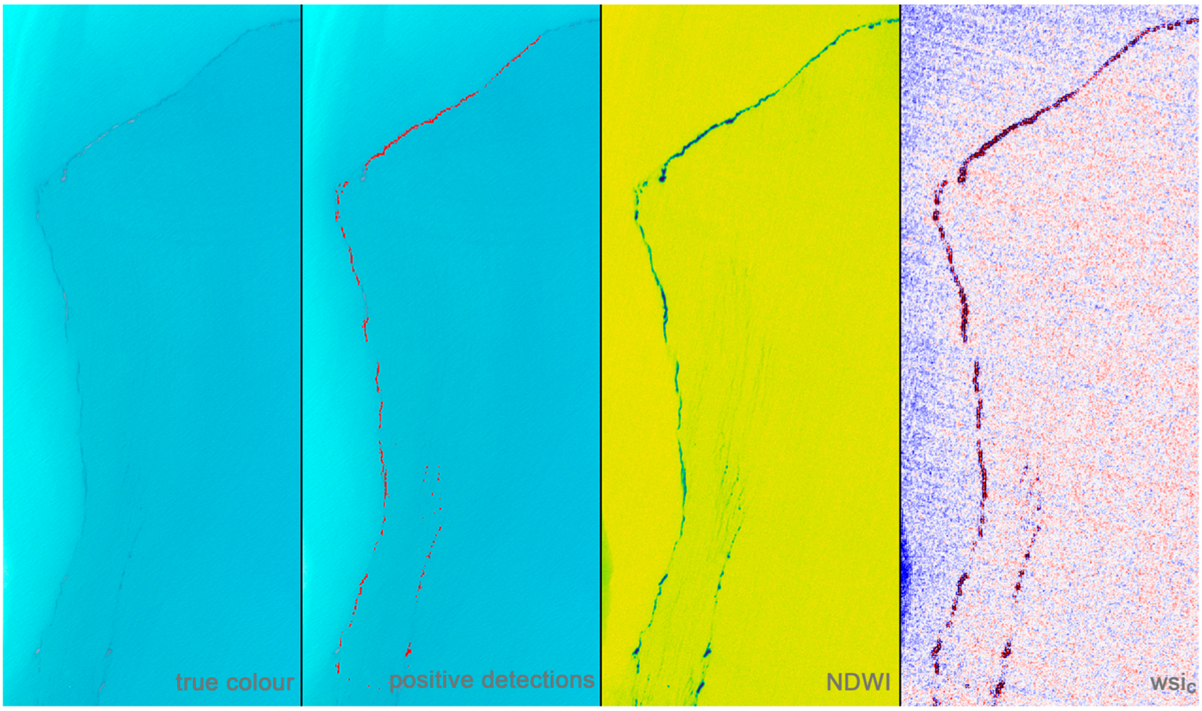

Fig. S25 | Example of the set of four images automatically provided by the PoC detection processor for each potential LW, based on radiometric and contextual features. From left to right: Sentinel-2 L1c true colour, same with an overlay of image pixels with positive detection (in red), NDWI, and wsi$_c$ component of WSI (see section S2.1.7). The filament shown in the example corresponds to a 7-km long LW found on 31 October 2018 off Piave River (Fig. 2).

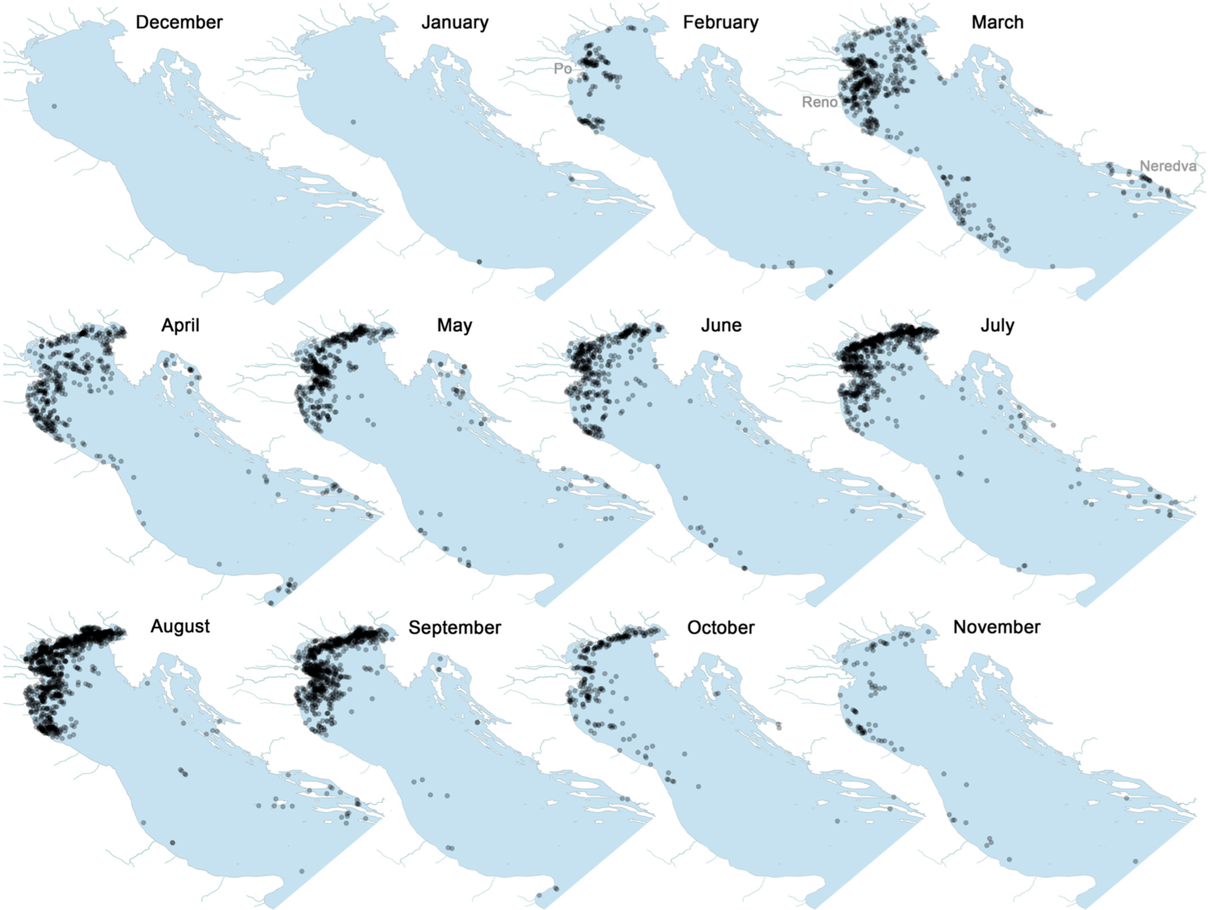

Fig. S26 | LW detections per month accumulated over the whole observation period (2015-2021) in the North Adriatic Sea. LWs are plotted as semi-transparent black dots proportional to their size. Main rivers are outlined.

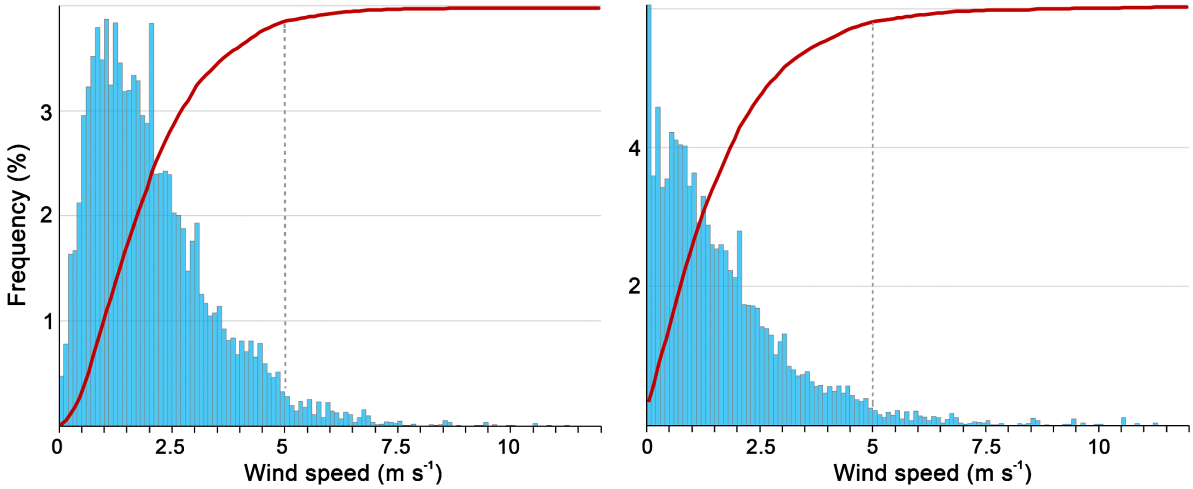

Fig. S27 | Wind conditions for the LWs. Frequency histograms of LW detections (in relative abundance) is shown as a function of the concurrent wind speed (left), and after normalising with the wind-speed histogram over the entire Mediterranean Sea during the observation period (right). Dashed line at 5 m·s^{-1} marks the upper wind limit here considered suitable for LW formation. Note the apparent presence of two domes in the wind histograms, a major dome at low speeds, and a second dome between 3.5 and 5 m·s^{-1}, which might be related to the range of activation of wind-induced Langmuir circulation[80], a proposition that requires further quantitative testing. Red lines represent the cumulative frequency up to 100%.

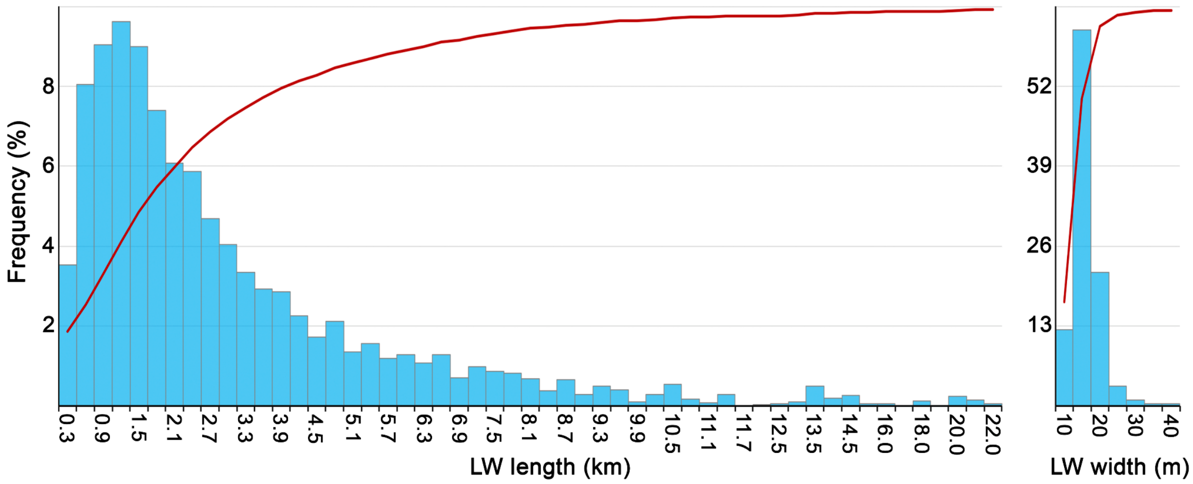

Fig. S28 | Size of the LWs. Frequency histograms for length and width of LWs. Relative frequencies were calculated in terms of area in relation to the total area covered by the 14,374 LWs detected over the study period. Red lines represent the cumulative frequency up to 100%.

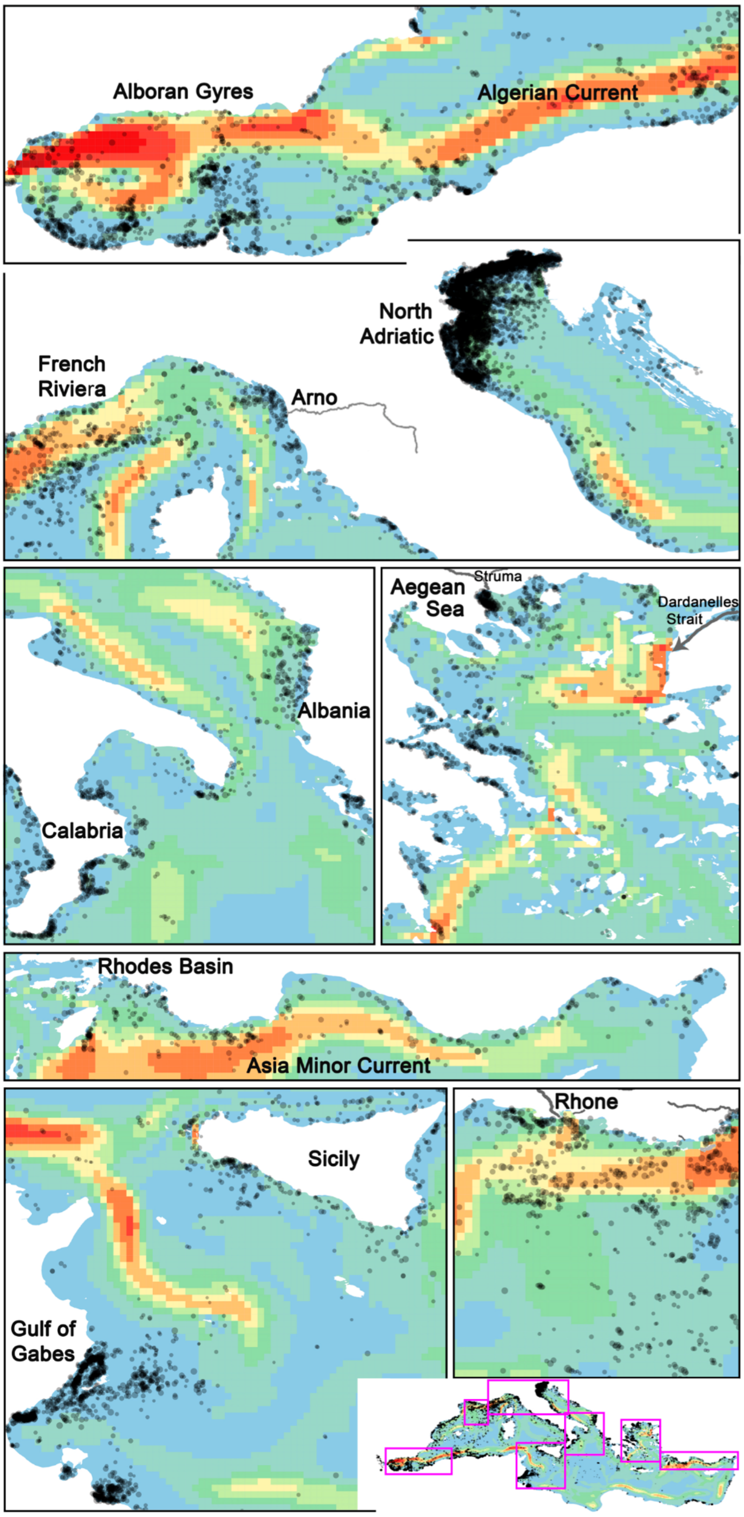

Fig. S29 | LW detections in different regions of the Mediterranean Sea. Zoomed areas are marked with rectangles on the lower right map. Symbols for LW size and colour codes for MKE are equivalent to those used in Fig. 3. Boundary currents may act as barriers to the propagation of litter both from land to sea and from sea to land. This blocking effect has been previously described in waters off the French Riviera[81]. On some cases, detections drew gyres at different scales, as in the large gyres in the Alboran Sea or the small gyre found in the Gulf of Squillace (Calabria, Italy).

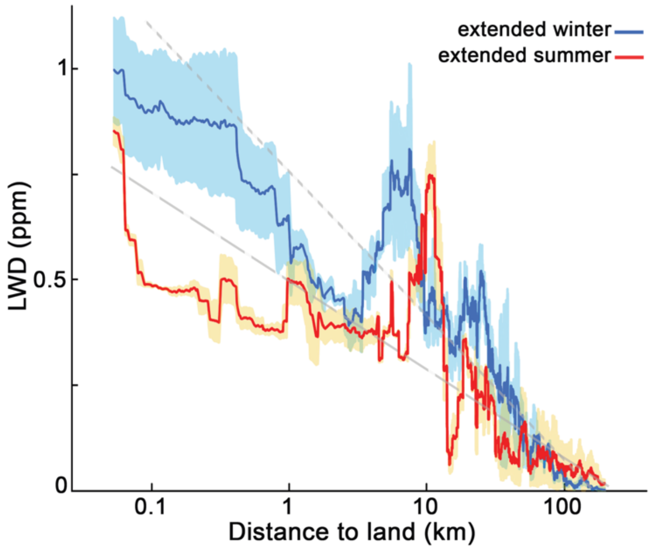

Fig. S30 | Seasonal patterns of LWD in relation to distance to land. Extended summer encompasses spring and summer, and extended winter comprises autumn and winter. Shaded areas with each line show the range between spring and summer in extended summer, and between autumn and winter in extended winter. Dashed lines correspond to the exponential decay fit for extended winter ($r = 0.928$, $p < 0.01$) and extended summer ($r = 0.816$, $p < 0.01$). Note that the litter build-up around 10 km from land was maintained seasonally, with small shifts likely associated with changes in intensity and position of boundary currents[82].

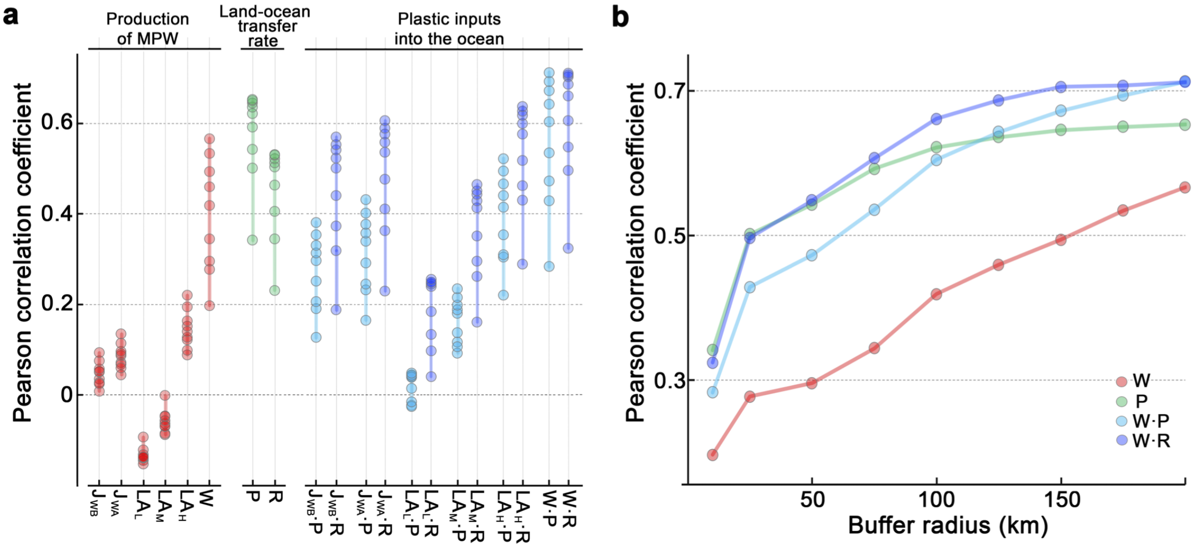

Fig. S31 | Pearson correlation test between nearshore LWD and different models related to land-based sources of marine litter. a, Pearson correlation test between nearshore LW and three different metrics related to land-based sources of ML: land-based production of MPW (*MPWP*, in red), land-to-ocean transfer rate (η, in green), and plastic inputs from land into the ocean (*PI*, in blue). Different models were used to estimate each of these three metrics. Acronyms for the *MPWP* models (JWB, JWA,LAL, LAM, LAH,W) are defined in Table S32. η was estimated as a function of precipitation (*P*) and surface runoff (*R*), while *PI* was derived from the combinations of *MPWP* and η. Models are described in "Spatio-temporal drivers" subsection in Methods (main text). Dots in each model correspond to different buffer radius, *i.e.* 10, 25, 50, 75, 100, 125, 150, 175 and 200 km. **b**, Pearson coefficients in relation to buffer radius are shown for the best models in each metric.

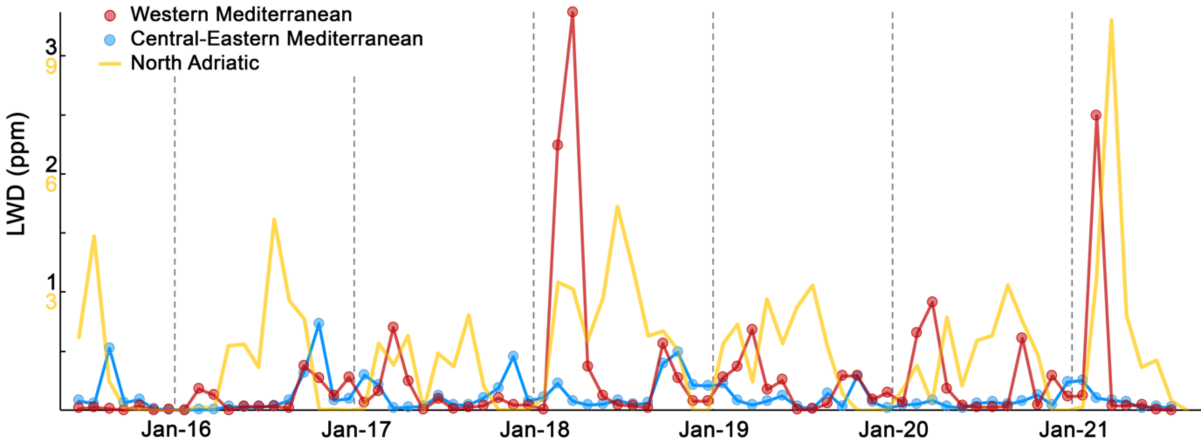

Fig. S32 | Monthly series of LWD in large covarying regions, Western Mediterranean and Central-Eastern Mediterranean. North Adriatic was treated separately since it showed a particular variability, with recurrent summer maxima decoupled from the spring and autumn maxima in the Western and Central-Eastern regions. A specific LWD scale (yellow numbers) is used for North Adriatic**.**

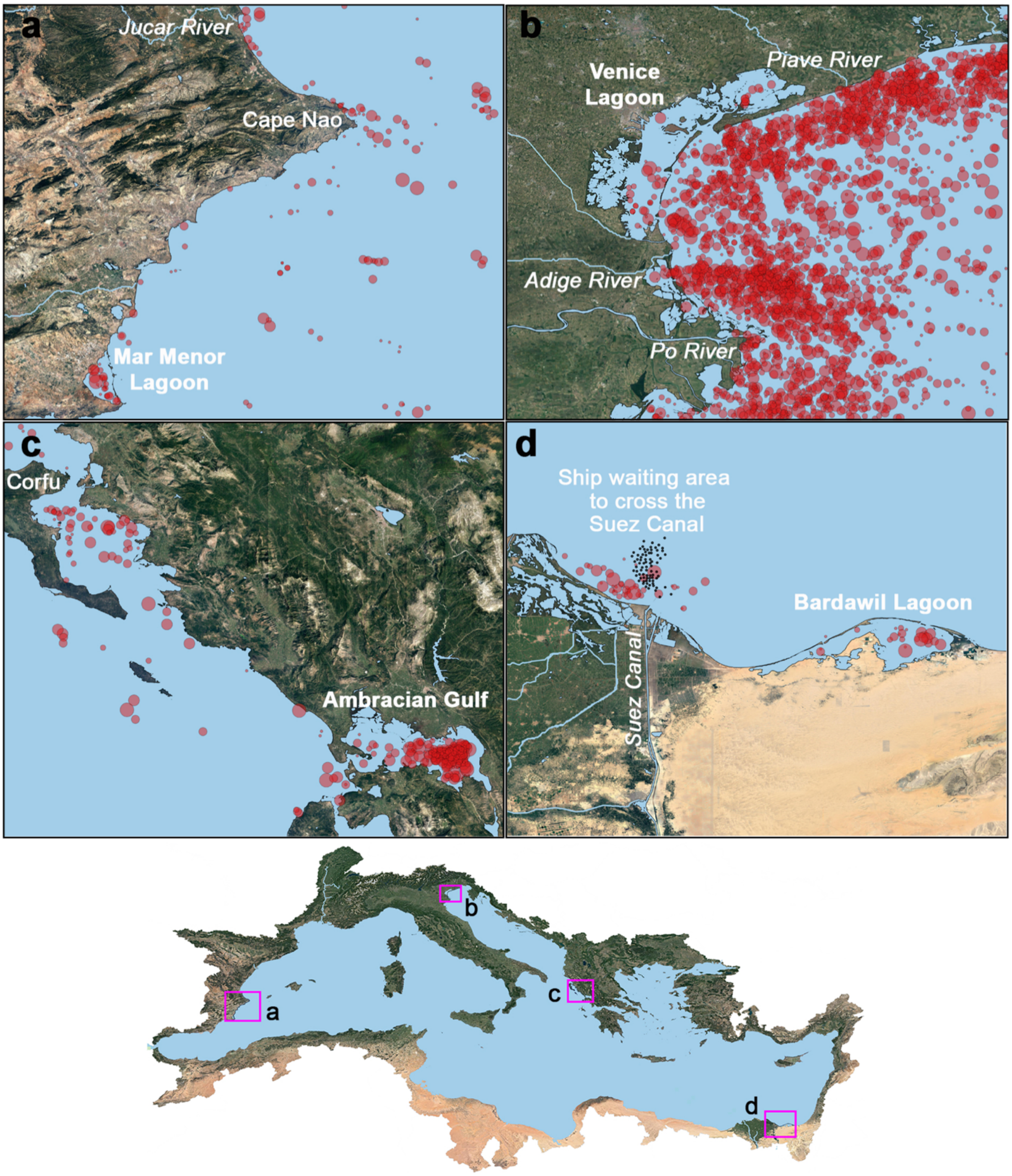

Fig. S33 | LW detections in lagoons and other coastal singularities. **a**, Mar Menor lagoon, Jucar River outlet and Cape Nao (Spain). **b**, Venice Lagoon and outlets of alpine rivers (Italy). **c**, Ambracian Gulf and Corfu island (Greece). **d**, Bardawil Lagoon and ship waiting area of Suez Canal (Egypt). The semi-transparent red dots represent LW detections accumulated during the observation period. Black dots in d correspond to the distribution of waiting ships on 25 March 2021. Zoomed areas are marked with pink rectangles on the lower map.

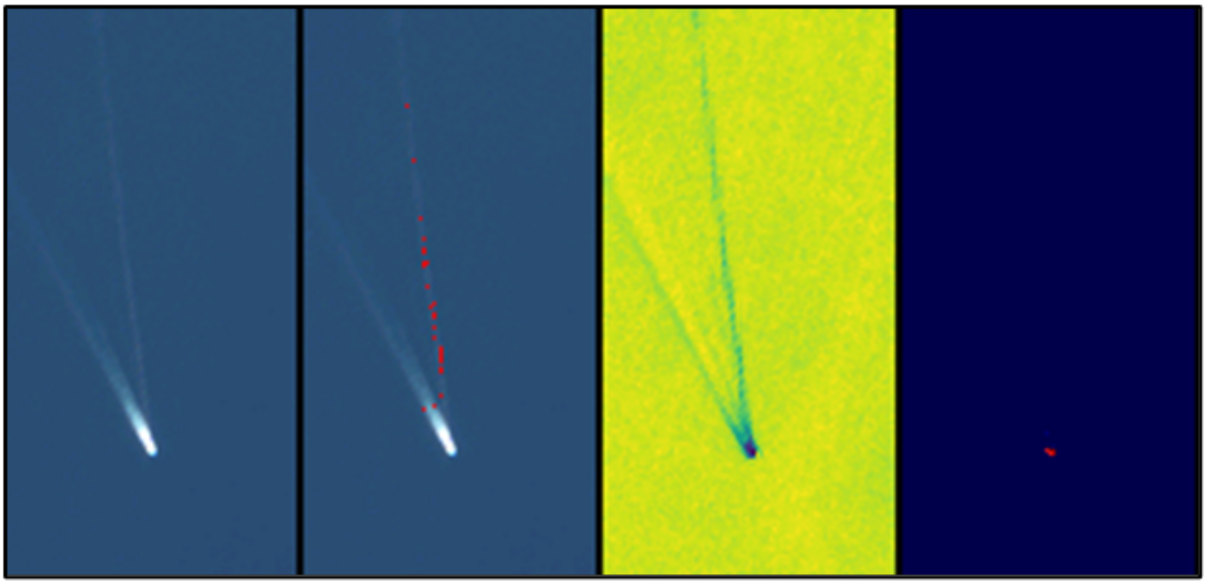

Fig. S34 | Example of a false positive caused by ship trailing waves. From left to right: Sentinel-2 L1c true colour, same with an overlay of image pixels with positive detection (in red), NDWI, and wsi_c component of WSI.

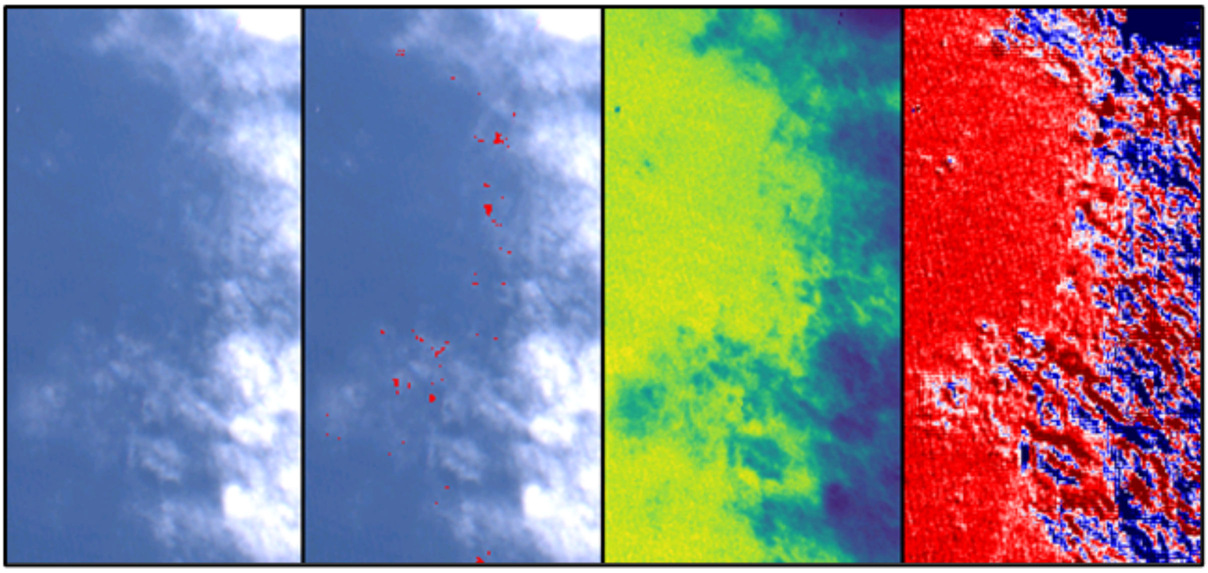

Fig. S35 | Example of a false positive caused by cloud edges. From left to right: Sentinel-2 L1c true colour, same with an overlay of image pixels with positive detection (in red), NDWI, and wsi_c component of WSI.

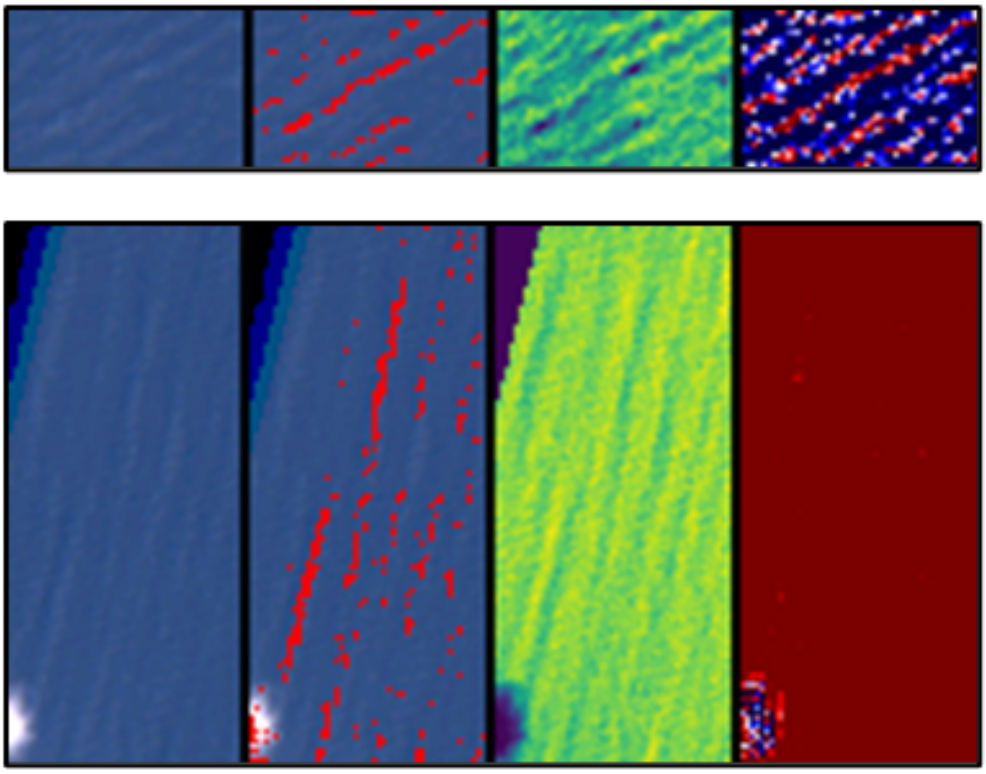

Fig. S36 | Example of a false positive due to wave glint. From left to right: Sentinel-2 L1c true colour, same with an overlay of image pixels with positive detection (in red), NDWI, and wsi_c component of WSI.

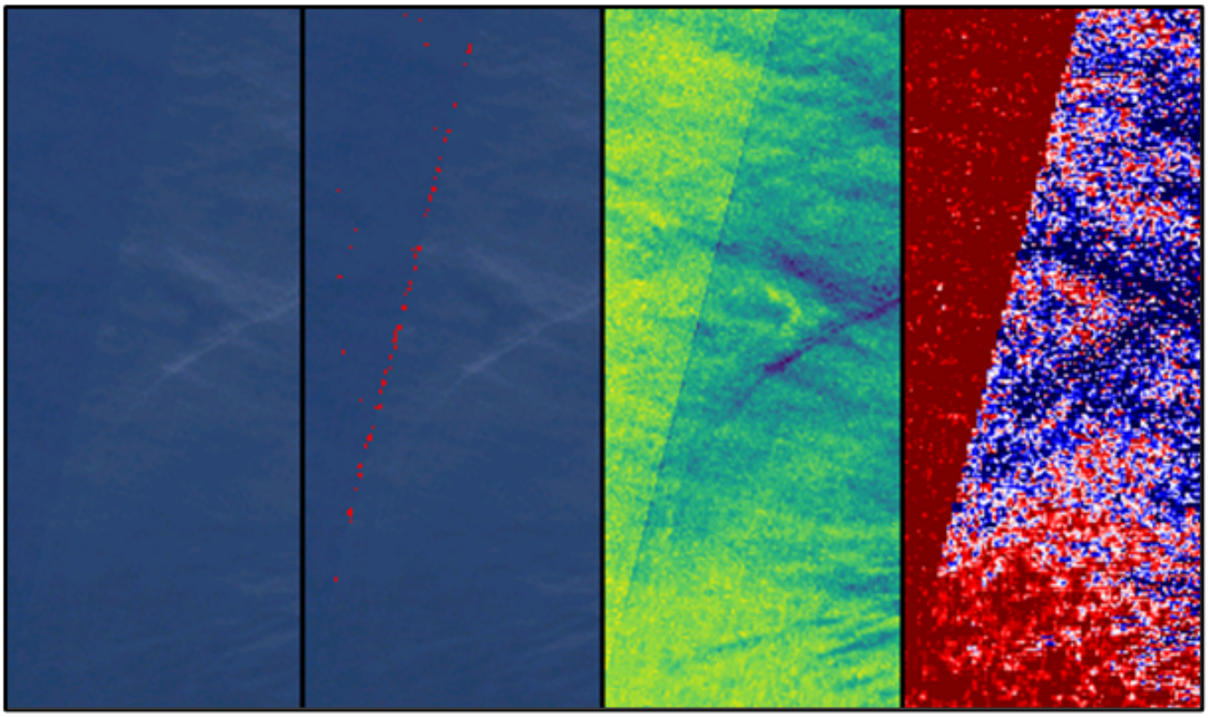

Fig. S37 | Example of a false positive caused by detector footprint. From left to right: Sentinel-2 L1c true colour, same with an overlay of image pixels with positive detection (in red), NDWI, and wsi_c component of WSI.

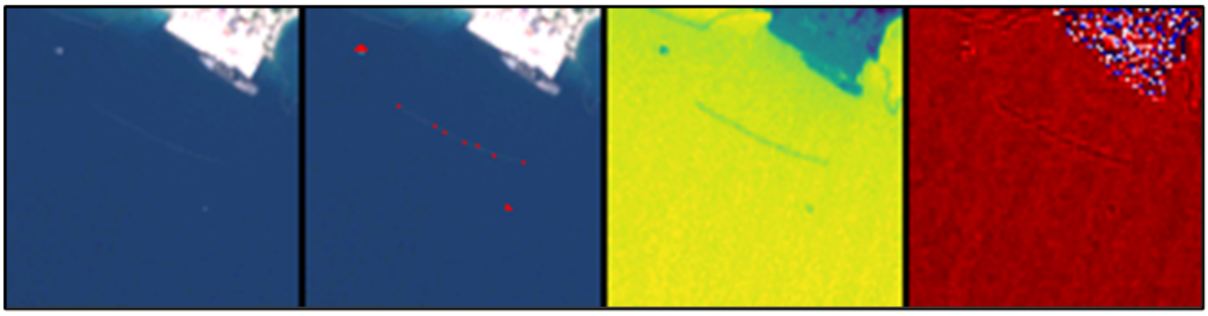

Fig. S38 | Example of a false positive due to man-made structures. From left to right: Sentinel-2 L1c true colour, same with an overlay of image pixels with positive detection (in red), NDWI, and wsi_c component of WSI.

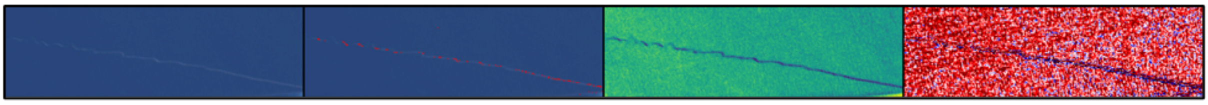

Fig. S39 | Example of a false positive caused by airplane exhausts. From left to right: Sentinel-2 L1c true colour, same with an overlay of image pixels with positive detection (in red), NDWI, and wsi_c component of WSI.

SUPPLEMENTARY TABLES

Mission need	Observational need	Requirement
Coverage of the global ocean (main gyres, Mediterranean Sea, Sea of Japan)	Field of access	Sun-synchronous circular orbit<br>Altitude TBD
Large areas of interest (eddies, 1 Mkm^2)	Swath	(G) 200 km<br>(T) 20 km
Slow variability at global scale	Revisit on coverage	A few months
High variability at mesoscale	Revisit on areas of interest	(G) day<br>(T) week
Detection of large aggregates	GSD	(G) 20 m<br>(T) 200 m
Detection of concentrations of small debris	SNR (SWIR)	(G) 800<br>(T) 400
Detection and characterisation of plastic polymers	Spectral bands<br>(= centre of band, width ≈10 nm TBC)	{1197, 1216, 1235 nm}<br>{1702, 1732, 1742 nm}

Table S1 | Preliminary EO4ML requirements based on analysis results.
G: Goal value; T: Threshold value.

	MODIS	MERIS	OLCI
Agency	NASA	ESA	ESA
Launch	Terra 1999<br>Aqua 2002	2002	2016
Lifetime-end	Terra 15<br>Aqua 13	2012	7.5 years
Platform / Mission	EOS/AM-1 (Lockheed Martin) (Terra)<br>EOS/PM-1 (TRW / Northrop Grumman) (Aqua)	ENVISAT	Sentinel-3
Orbit Altitude	705 km	800 km	814.5 km
Orbit Type	SSO, 10:30 AM LTDN (Terra)<br>SSO, 01:30 PM LTAN (Aqua)	SSO	SSO, 10 :00 AM LTDN
Orbit Inclination	98.5° (Terra)<br>98.2° (Aqua)		98.65º
Detector	Silicon (VNIR)<br>Photovoltaic MCT (SWIR/MWIR)<br>Photoconductive MCT (LWIR)	Back-illuminated CCD55-20<br>780 x 576 @22.5 µm	MERIS heritage<br>Back-illuminated CCD55-20<br>780 x 576 @22.5 µm
Imaging Principle	Whisk-broom	Push-broom imaging spectrometer	Push-broom imaging spectrometer
Spatial Resolution	250 m (bands 1 and 2)<br>500 m (bands 3-7)<br>1 km (bands 8-36)	RR: 1040 m x 1200 m<br>FR: 260 m x 300 m	300 m
Swath	2330 km	1150 km	1270 km
Spectral Resolution	(cf. tables below)	1.25 nm sampling interval<br>1.5 nm (FWHM)	1.25 nm sampling interval
Spectral Band	(cf. tables below)	390 – 1040 nm	390 – 1040 nm
Number of bands	36	15 (programmable in position (390-1040 nm) and width (1.25 nm-30 nm)	21 (nominal Earth view)<br>45 (spectral campaigns)
SNR	(cf. tables below)	150 – 1500	190 – 2000
Radiometric Accuracy		< 2% in reflectance<br>0.05% relative spectral accuracy	< 2% w.r.t Sun<br>0.1% stability / orbit
MTF		> 0.3	0.28
Dynamic Range	12 bits		
Data Rate	10.6 Mbps (peak daytime)<br>6.1 Mbps (orbital average)	1.6 Mbit/s (RR)<br>24 Mbit/s (FR)	
Dimensions	1.0 x 1.6 x 1.0 m	1.8 x 0.9 x 1.0 m	1.3 m^3
Mass	228.7 kg	200 kg	150 kg

Table S2 | Inventory of some past and on-going ocean colour missions considered in this study.

Primary Use	Band	Bandwidth (nm)	Spectral Radiance (W/m²/µm/sr)	SNR
Land/Cloud/Aerosols Boundaries	1	620 - 670	21.8	128
	2	841 - 876	24.7	201
Land/Cloud/Aerosols Properties	3	459 - 479	35.3	243
	4	545 - 565	29.0	228
	5	1230 - 1250	5.4	74
	6	1628 - 1652	7.3	275
	7	2105 - 2155	1.0	110
Ocean Colour/ Phytoplankton/ Biogeochemistry	8	405 - 420	44.9	880
	9	438 - 448	41.9	838
	10	483 - 493	32.1	802
	11	526 - 536	27.9	754
	12	546 - 556	21.0	750
	13	662 - 672	9.5	910
	14	673 - 683	8.7	1087
	15	743 - 753	10.2	586
	16	862 - 877	6.2	516
Atmospheric Water Vapor	17	890 - 920	10.0	167
	18	931 - 941	3.6	57
	19	915 - 965	15.0	250
Surface/Cloud Temperature	20	3660 - 3840	0.45 (300K)	0.05
	21	3929 - 3989	2.38 (335K)	2.00
	22	3929 - 3989	0.67 (300K)	0.07
	23	4020 - 4080	0.79 (300K)	0.07
Atmospheric Temperature	24	4433 - 4498	0.17 (250K)	0.25
	25	4482 - 4549	0.59 (275K)	0.25
Cirrus Clouds Water Vapor	26	1360 - 1390	6.00	150
	27	6535 - 6895	1.16 (240K)	0.25
	28	7175 - 7475	2.18 (250K)	0.25
Cloud Properties	29	8400 - 8700	9.58 (300K)	0.05
Ozone	30	9580 - 9880	3.69 (250K)	0.25
Surface/Cloud Temperature	31	10780 - 11280	9.55 (300K)	0.05
	32	11770 - 12270	8.94 (300K)	0.05
Cloud Top Altitude	33	13185 - 13485	4.52 (260K)	0.25
	34	13485 - 13785	3.76 (250K)	0.25
	35	13785 - 14085	3.11 (240K)	0.25
	36	14085 - 14385	2.08 (220K)	0.35

Table S3 | List of spectral band characteristics and uses from optical missions.

	HYPERION	CHRIS	HySI	HICO
Agency	NASA	BNSC / ESA	ISRO	ONR / NRL / NASA
Launch	2000	2001	2008	2009
Lifetime-end	16 years	End of 2017	4.5 years	2014
Platform	EO-1<br>(ATK)	Microsat PROBA1<br>(Verhaert)	Microsat IMS-1	ISS
Orbit Altitude	705 km	542 - 657 km	632 km	350 - 420 km
Orbit Type	SSO 10:15 AM<br>LTDN	SSO elliptical polar<br>10:30 AM LTDN	SSO 09:30 AM<br>LTDN	Near circular
Orbit Inclination	98.7º	97.9º	97.94º	51.6º
Detector	CCD 60 (spectral) x 250 (spatial) (VNIR)<br>MCT 160 (spectral) x 250 (spatial) (SWIR)	e2v CCD25-20 1152 x 780<br>Useful 748 x 748	CMOS 512x250	e2v CCD97 512x512 CCD
Spatial Resolution	30 m	34 m	505 m	100m (at 20 km)
Swath	7.5 km	13 - 15 km	130 km	51 km (at 420 km)
Spectral Resolution	≈10 nm [FWHM]<br>(VNIR/SWIR)	1.25 nm (at 400 nm)<br>11 nm (at 1000 nm)	8.4 nm (sampling)<br>≈20 nm (bandwidth)	3.6 nm [FWHM]<br>5.73 nm 3-pixel binning
Spectral Band	400-1000 nm<br>(VNIR)<br>900-2500 nm<br>(SWIR)	400-1050 nm	410 – 965 nm	350-1080 nm
Number of bands	220	63 at 34 m GSD	64	128
SNR			820 (Lsat)	200 (400-600 nm)
MTF			Square Wave Response at Nyquist 35%	
Dynamic Range	12 bits	12 bits	15 bits	14 bits
Data Rate			32 Mbit/s	
Data Volume	75 Mo / image 7.5 x 19.8 km			
Daily Coverage			130 x 130 km images	9700 km² / image (nadir)
Revisit				
Dimensions	39 x 75 x 66 cm	20 x 26 x 79 cm	15 x 20 x 17 cm	≈0.8 x 0.8 x 0.8 m
Mass	49 kg	14 kg	4 kg	41 kg
Applications	Mining (remote mineral exploration), geology, forestry, agriculture (crop yield), and environmental management	General understanding of spectral reflectance through BRDF data. Vegetation mapping, water quality, air quality and pollution monitoring. Coastal waters: suspended particulates, *Chl-a*	Survey and monitoring of India's natural resources: agriculture, forestry and ocean monitoring	Observation of coastal ocean, estuaries and adjacent landforms: river plumes, algal blooms, bathymetry, benthic bottom types

Table S4 | List of ongoing and past hyperspectral missions considered in this study.

	PRISMA	HISUI	EnMAP	SHALOM
Agency	ASI	METI	DLR	ISA / ASI
Launch	2018	2019	2020	2021
Lifetime-end	5 years	3 years	5 years	
Platform	Type MITA/AGILE (OHB-Italia)	ISS	LEOBus-1000 (OHB-Germany)	OPTSAT-3000
Orbit Altitude	615 km	400 km	652 km	640 km
Orbit Type	SSO, 10:30 AM LTDN	Near circular	SSO, 11:00 AM LTDN	SSO, 10:30 AM LTDN
Orbit Inclination	97.851º	51.6º	97.9655º	97.96º
Detector	1000x256	CMOS (VNIR) MCT (SWIR)	Fairchild CMOS (VNIR), AIM MCT (SWIR)	SOFRADIR 1000x256 MCT (VNIR, SWIR)
Spatial Resolution	30 m	30 m	30 m	5 - 10 m
Swath	30 km	30 km	30 km	5 - 10 km
Spectral Resolution	12 nm (VNIR, SWIR)	10 nm (VNIR)<br>12.5 nm (SWIR)	6.5 nm (VNIR)<br>10 nm (SWIR)	5-10 nm (VNIR)<br>10 nm (SWIR) [FWHM]
Spectral Band	0.40-2.50 μm	0.40-2.50 μm	0.42-2.45 μm	0.40-2.50 μm
Number of bands	66 (VNIR)<br>171 (SWIR)	185	88 (VNIR)<br>154 (SWIR)	256-275
SNR	200 (400-1000 nm)<br>500 (at 650 nm)<br>200 (1000-1750 nm)<br>400 (at 1550 nm)<br>100 (1950-2350 nm)<br>200 (at 2100 nm)	450 (at 620 nm)<br>300 (at 2100 nm)	500 (at 495 nm)<br>150 (at 2200 nm)	200-300 (400-1000 nm)<br>600-1000 (at 650 nm)<br>200-300 (1000-1750 nm)<br>400-600 (at 1550 nm)<br>100-200 (1950-2350 nm)<br>200-500 (at 2100 nm)
MTF	0.18 (along track)<br>0.34 (across track)	0.2	25%	
Dynamic Range	12 bits	12 bits	14 bits	12 - 16 bits
Data Rate	0.6 Gbps	0.4 Gbps<br>(70% compression)	866 Mbps	(62.5% compression)
Data Volume		690 GB/day	400 GB/day	6.6 TB/day
Daily Coverage	200,000 km²		5000 km swath length / day	200,000 km²
Revisit	6 days		4 days	4 days
Dimensions	770 x 590 x 780 mm<br>210 mm telescope	1485 x 950 x 1380 mm	1.8x1.2x0.7 m	650 mm telescope
Mass	90 kg	168 kg	≈350 kg	
Applications	Pre-operational mission<br>Technology demonstration<br>Validation of the end-to-end processing chain to enable the development of applications based on hyperspectral data	Energy and resources exploration<br>Environmental monitoring (corals, carbon dynamics)<br>Agriculture (yield, quality, growth)<br>Forestry	Climate change impacts and management. Land-cover. Biodiversity and ecosystems. Water availability and quality. Natural resources. Geohazard, risk assessment	Commercial applications in the field of:<br>Environment quality<br>Crisis monitoring<br>Resources exploration<br>Monitoring water bodies

Table S5 | Ongoing and future hyperspectral missions considered in the present study.

	GLAS	CALIOP	ALADIN	ATLAS	ATLID	MERLIN
Agency	NASA	NASA / CNES	ESA	NASA	ESA	CNES / DLR
Launch	2003	2006	2018	2018	2019	2021
Lifetime-end	2010	End of 2017	3 years	5 -7 years	3 -4 years	3 years
Mission	ICESat	CALIPSO	AEOLUS	ICESat-2	EarthCare	MERLIN
Orbit Altitude	600 km	705 km	320 km	496 km	393.14 km	506 km
Orbit Type	Near-polar	SSO 13:30 LTAN	SSO 18:00 LTAN	Near-polar frozen	SSO 14:00 LTDN	SSO 06:00 AM/PM
Orbit inclination	94º	98.05º	96.97º	92º	97.05º	97.4º
Measurement Concept	Altimeter	Orthogonal polarization	Doppler	Photon-counting altimeter	High Spectral Resolution	Integrated Path Diff.-Absorption
Laser Type	Doubled Nd:YAG	Doubled Nd:YAG	Tripled Nd:YAG	Doubled	Tripled Nd:YAG	Converted Nd:YAG
Wavelength	1064 nm / 532 nm	1064 nm / 532 nm	355 nm	532 nm	355 nm	1645 nm
Pulse Energy	75 mJ / 35 mJ	110 mJ	60 mJ	2 mJ	38 mJ	9 mJ
Pulse Rep. Frequency	40 Hz	20.16 Hz	100 Hz	10kHz	51 Hz	20 Hz
Pulse Width	4 ns	20 ns	15 ns	1.5 ns	25 ns	
Laser Footprint	66 m (@1064nm)	70 m		10 m		100 m
Horizontal Resolution	170 m	333 m	3.5 km mean over 50 km	0.7 m	285 m mean over 10 km	350 m mean over 50 km
Telescope Aperture	1 m	1 m	1,5 m	0.8 m	620 mm	690 mm
Telescope iFOV	375 µrad	130 µrad	22 µrad		25 µrad	
Mass	298 kg	156 kg	460 kg	298 kg	389 kg	119 kg
Power	330 W	124 W	840 W	300 W	310 W	150 W
Main Objectives	Ice, topography, clouds, aerosols	Clouds and aerosols	Wind	Ice sheet, sea ice, vegetation	Clouds and aerosols	Atmospheric methane

Table S6 | List of ongoing and future LiDAR missions considered in this study.

Sensor Type	Pros	Cons
**Visible Imager**	Very-high resolution commercially available<br><br>For big objects (majority of mass, future microplastics)<br><br>Detection by image processing	Debris < GSD detectable as an anomaly in the pixel; not identifiable in such a situation.<br><br>Need to cover very large areas vs. swath<br><br>Deluge of data except if on-board detection or pre-detection
**Multispectral**	Heritage on ocean colour missions<br><br>Detection of concentrations + very large aggregates > GSD<br><br>Large bands => more signal (better SNR)<br><br>GEO mission to increase revisit on regional areas (intraday @250 m GSD for GOCI-2)	Needs a precise knowledge of the spectral bands<br><br>GSD limited to a few (tens of) metres in LEO<br><br>Limited spectral characterisation
**Hyperspectral**	Opportunity to use data from the several missions to be launched in the coming years<br><br>Detection of concentrations + very large aggregates<br><br>Extended spectral characterisation (better separation capability in coastal zones)<br><br>Could even be band-agnostic	Limited heritage (incl. for processing) and technology more complex than multispectral<br><br>Detection limited to very high concentrations (SN
**LiDAR**	Opportunity to use data from coming missions.<br><br>Underwater measurement (retrieval of concentrations vs. depth) + Surface.<br><br>Day/night acquisitions	High complexity and cost<br><br>Difficult to disentangle useful signal from other sources (in particular | spectral analysis using fluorescence or Raman)<br><br>Low spatial resolution (few hundreds of metres)

Table S7 | Pros and cons of the candidate techniques for EO4ML.

Scenario	Compatible	Comments
Detection and identification of large ML items at the ocean	Poorly	Only significantly large items
Detection and quantification of concentrations of ML at global scale	Fully	If enough SNR is possible at the level of the expected concentrations of ML
Detection and monitoring of hot spots and accumulation zones	Fully	Provided that revisiting time requirement is met
Monitoring of river mouths as main input flow for ML	Partially	Only threshold criteria could be met, and with limitations to retrieval
Detection, monitoring and quantification of ML at shores and beaches	Poorly	Texture mixing in the pixel will be very challenging at the proposed spatial scales

Table S8 | Compatibility of ML scenarios with the proposed requirements.

Band	Band Center (nm)	Bandwidth (nm)
B1	1615	85
B2	1782	70
B3	2031	100

Table S9 | Bands used for T_1 and T_2 indices.

Index Component	LDPE	PP	PS	Reference
		f = 10%		
T1	0.780	0.797	0.717	0.407
T2	0.264	0.365	0.292	0.029
**Total (PMLI)**	**1.045**	**1.162**	**1.009**	**0.436**
		f = 1%		
T1	0.530	0.537	0.512	0.407
T2	0.076	0.099	0.098	0.029
**Total (PMLI)**	**0.606**	**0.636**	**0.610**	**0.436**
		f = 0.1%		
T1	0.421	0.424	0.421	0.407
T2	0.037	0.043	0.045	0.029
**Total (PMLI)**	**0.459**	**0.467**	**0.466**	**0.436**

Table S10 | Value of the indices T_1, T_2 and PMLI for three plastic polymers (LDPE, PP and PS) and the reference (*i.e.*, plastic-free seawater).

Index Component	LDPE	PP	PS	Reference
		f = 10%		
T1	1.92	1.96	1.76	1
T2	9.08	12.54	10.04	1
**Total**	**2.40**	**2.67**	**2.31**	**1**
		f = 1%		
T1	1.30	1.32	1.26	1
T2	2.61	3.41	3.36	1
**Total**	**1.39**	**1.46**	**1.40**	**1**
		f = 0.1%		
T1	1.04	1.04	1.04	1
T2	1.28	1.47	1.53	1
**Total**	**1.05**	**1.07**	**1.07**	**1**

Table S11 | Normalized indices T_1 and T_2 for three plastic polymers (LDPE, PP and PS) against the reference of plastic-free seawater.

Band name	Band centre (nm)	Bandwidth (nm)
Blue	490	40
Green	560	35
Red	665	30

Table S12 | Candidate auxiliary spectral bands in the visible.

Band	Band Centre (nm)	Bandwidth (nm)
A	913	25
B	972	60

Table S13 | Additional candidate spectral bands for ML detection.

Concentration	False Negative	Accuracy on ρ
f = 1%	10%	$\sigma_\rho = 0.0481$
	5%	$\sigma_\rho = 0.0375$
f = 0.1%	10%	$\sigma_\rho = 0.0056$
	5%	$\sigma_\rho = 0.0044$

Table S14 | Required accuracy on ρ function of LDPE concentration and false negative rate.

Concentration	False Negative	Accuracy on ρ	Required SNR
f = 1%	10%	0.0481	21
	5%	0.0375	27
f = 0.1%	10%	0.0056	179
	5%	0.0044	227

Table S15 | Required SNR for given false negative rates and two LDPE concentrations.

Concentration	False Negative	Spatial Sampling	Accuracy on ρ	Required SNR	Compliance MSI	Compliance Improved Instrument
f = 1%	10%	10 m	0.0481	21	no	yes (no margin)
		20 m			yes	yes
	5%	10 m	0.0375	27	no	yes (no margin)
		20 m			yes (no margin)	yes
f = 0.1%	10%		0.0056	179	no	no
	5%		0.0044	227	no	no

Table S16 | Comparison of performance vs mission requirements for S2-MSI and expected future generation of medium resolution spectral sensors (with double SNR compared to S2-MSI).

Band #	Band Centre (nm)	Spectral Width (nm)	Spatial Resolution (m)
1	443	20	60
2	490	65	10
3	560	35	10
4	665	30	10
5	705	15	20
6	740	15	20
7	775	20	20
8	842	115	10
8a	865	20	20
9	940	20	20
10	1375	20	60
11	1610	90	20
12	2190	180	20

Table S17 | Copernicus S2-MSI spectral bands.

Image	Ranking				SZA	SAA	Water Vapour (kg / m^3)	Surface Pressure (HPa)	Ozone (kg / m^3)
	MEETC2	ACOLITE	SEN2COR	L1c					
Bali, Indonesia<br>50LRR<br>03/04/2018	1	2	4	3	28.64	61.88	41.49	1009.84	0.0054
Calabria, Italy<br>33SWC<br>22/10/2018	1	3	2	4	50.91	163.50			
Crete, Greece<br>35SKV<br>21/01/2019	1	3	2	4	58.20	159.60	15	1016.92	0.006
Omoa Bay, Honduras<br>16PCC<br>18/09/2020	1	4	2	3	23.96	124.65			
Marbella, Spain<br>30SVF<br>26/10/2017	1	2	2	3	50.36	165.08	11.92	1025.95	0.006
Po River, Italy<br>32TQQ<br>26/10/2017	1	4	4	3	41.17	156.75	31.38	1018.48	0.006

Table S18 | Summary of scores for different atmospheric corrections. L1C results were added for comparison purposes. Additionally, ancillary information was also considered in order to better understand results and potential correlation with specific observational conditions. Information under Image indicates site, corresponding S2-MSI tile and observation date.

Scale	Minimum Distance	Maximum Width
1	6	6
2	7	5
3	5	4
4	5	4
5 to 34	4	4

Table S19 | Routine 1 minimum distances and maximum widths at different scales.

Scale	Minimum Distance	Maximum Width
5 to 19	4	7
20 to 34	4	8

Table S20 | Routine 2 minimum distance and maximum widths for scale numbers. Only these scales (5 to 19; 20 to 34) were processed in this step.

Scale	Minimum Distance	Maximum Width
4 to 13	0	6

Table S21 | Routine 3.1 minimum distance and maximum widths for scale numbers. Only the scales from 4 to 13 was processed in this step.

Scale	Minimum Distance	Maximum Width
1	0	15
2	0	15
3	0	20

Table S22 | Routine 3.2 minimum distance and maximum widths for scale numbers. Only three scales were processed at this step.

Band	λ (nm)	Bandwidth (nm)	Regime	Purpose
B01	400	15	VIS	Aerosol correction, improved water constituent retrieval
B02	442	20	VIS	Chl absorption maximum, sediments, vegetation
B03	490	40	VIS	High Chlorophyll
B04	510	10	VIS	Chl, sediment, turbidity, red tide, seafoam max.
B05	560	35	VIS	Chlorophyll reference
B06	665	30	VIS	Chlorophyll (2nd Chl absorption max.), sediment, yellow substance/vegetation
B07	765	10	VIS	O2 absorption band, aerosol correction, atmospheric correction
B08	780	20	VIS/NIR	Atmospheric correction, aerosol correction
B09	826	70	NIR	Plastic polymers
B10	865	20	NIR	Atmospheric correction/aerosol correction, clouds
B11	885	10	NIR	Water vapor absorption reference band
B12	913	25	NIR	Plastic polymers
B13	945	20	NIR	Water vapor absorption, atmospheric correction and aerosol correction
B14	972	60	NIR	Plastic polymers, seafoam reference
B15	1100	50	NIR	Plastic polymers
B16	1205	40	NIR	Plastic polymers reference, driftwood
B17	1325	50	NIR	Plastic polymers, driftwood
B18	1375	30	NIR	Cirrus and cloud detection
B19	1515	65	NIR	Plastic polymers
B20	1615	85	SWIR	Plastic polymers
B21	1782	70	SWIR	Plastic polymers, driftwood reference
B22	2031	100	SWIR	Plastic polymers
B23	2251	170	SWIR	Plastic polymers, driftwood

Table S23 | Optimal bandset of an optical EO4ML mission. The minimum set of 6 bands required to achieve a plastic detection threshold of about 1%-pixel coverage is marked with thicker lines. The rest of the bands are dedicated to improve plastic detection and spectral unmixing at pixel level, as well as to support atmospheric correction and cloud detection. The 23 bandwidths and band-centres are graphed in Fig. S24.

		PoC Processor		
		True	False	Total
Human Operator	Positive	481	3,857	4,338
	Negative	4,001	67	4,068
	Total	4,482	3,924	8,406

Table S24 | Confusion matrix for the results of the Test Run 1. Human operators checked individually all automatically identified filaments to perform a human-driven classification.

		PoC Processor		
		True	False	Total
Human Operator	Positive	691	2,122	2,813
	Negative	9,329	141	9,470
	Total	10,020	2,263	12,283

Table S25 | Confusion matrix for the results of the Test Run 2. Human operators checked individually all automatically identified filaments to perform a human-driven classification.

		PoC Processor		
		True	False	Total
Human Operator	Positive	704	1,062	1,766
	Negative	7,652	110	7,762
	Total	8,356	1,172	9,528

Table S26 | Confusion matrix for the results of the Test Run 3. Human operators checked individually all automatically identified filaments to perform a human-driven classification.

Metric	Formulae	Test Run 1	Test Run 2	Test Run 3
Sensitivity	TP / P	87.77%	83.05%	86.47%
Selectivity	TN / N	50.92%	81.47%	87.81%
Precision	TP / (TP + FP)	11.09%	24.56%	39.86%
Miss Rate	FN / P	12.23%	16.95%	13.51%
Fall-out	FP / (FP + TN)	49.08%	18.53%	12.19%
Accuracy	(TP + TN) / (P + N)	53.32%	81.58%	87.72%

Table S27 | ROC metrics for the Test Run 1, 2 and 3.

Name	Data Type	Dimensions	Units
bands	Char	(n_bands, 3)	n/a
Id	Int16 (short)	n_filaments	counter
Limits	Int16 (short)	(n_filaments, 4)	(X, Y) coordinates
x_centroid	Int16 (short)	n_filaments	X-coordinate
y_centroid	Int16 (short)	n_filaments	Y-coordinate
lat_centroid	Float64 (double)	n_filaments	Degrees North
lon_centroid	Float64 (double)	n_filaments	Degrees East
est_surface	Int32 (integer)	n_filaments	m^2
est_length	Float32 (float)	n_filaments	m
est_width	Float32 (float)	n_filaments	m
vacance_ratio	Float32 (float)	n_filaments	%
pixels_per_filament	Int16 (short)	n_filaments	counter
flag	Int16 (short)	n_filaments	Boolean (0, 1)
mean_spec	Float32 (float)	(n_filaments, n_bands)	Reflectance
std_spec	Float32 (float)	(n_filaments, n_bands)	Reflectance
pixel_x	Int16 (short)	(n_filaments, n_max_pixels)	X-coordinate
pixel_y	Int16 (short)	(n_filaments, n_max_pixels)	Y-coordinate
pixel_lat	Float64 (double)	(n_filaments, n_max_pixels)	Degrees North
pixel_lon	Float64 (double)	(n_filaments, n_max_pixels)	Degrees East
pixel_spec	Float32 (float)	(n_filaments, n_max_pixels, n_bands)	Reflectance

Table S28 | Data values recorded for each filament in the netCDF output.

		PoC Processor		
		**True**	**False**	**Total**
**Human Operator**	**Positive**	11,759	74,328	86,087
	**Negative**	619,648	2,623	622,271
	**Total**	631,407	76,951	708,358

Table S29 | Confusion matrix for the results of the full run of the PoC processor over the S2-MSI imagery in the Mediterranean Sea from 4 July 2015 to 21 September 2021 (75 months). Human operators checked individually all automatically identified filaments to perform a human-driven classification.

Metric	Formulae	Results
Sensitivity	TP / P	81.76%
Selectivity	TN / N	89.30%
Precision	TP / (TP + FP)	13.66%
Miss Rate	FN / P	18.24%
Fall-out	FP / (FP + TN)	10.71%
Accuracy	(TP + TN) / (P + N)	89.14%

Table S30 | ROC metrics for the results of the full run of the processor over the S2-MSI time series in the Mediterranean Sea (from 4 July 2015 to 21 September 2021).

		PoC Processor			
		Run	True	False	Total
Human Operator	Positive	Test	7.39%	11,14%	18.53%
		Full	1.65%	10.49%	12.14%
	Negative	Test	80.32%	1.15%	81.47%
		Full	87.49%	0.37%	87.86%
	Total	Test	87.71%	12.29%	100.00%
		Full	89.14%	10.86%	100.00%

Table S31 | Proportion of filaments classified in each category from the Processor validation test and the full processing run over all tiles in the Mediterranean Sea.

MPWP model	Source	p_i	g_c, f_c, in_c	l
J_{WB}	Jambeck *et al.*[83]	50-km band from the coastline, Landscan dataset[86]	World Bank[87], International Monetary Fund[88]	2%
J_{WA}	Jambeck *et al.* [83]; Lebreton & Andrady[84]	50-km band from the coastline, Landscan dataset[86]	Waste Atlas[89], International Monetary Fund[88]	2%
LA_L	low-rage model in Lebreton & Andrady[84]	whole basin, Landscan dataset[86]	Waste Atlas[89], International Monetary Fund[88]	0.1%
LA_M	mid-range model in Lebreton & Andrady[84]	whole basin, Landscan dataset[86]	Waste Atlas[89], International Monetary Fund[88]	2%
LA_H	high-range model in Lebreton & Andrady[84]	whole basin, Landscan dataset[86]	Waste Atlas[89], International Monetary Fund[88]	10%
W	Weiss *et al.*[85]	whole basin, Landscan dataset[86]	13.5 kg inhab^{-1} y^{-1} [52]	N.A.

Table S32 | Characteristics and data sources of the six models explored for estimating production of MPW (*MPWP*). Models are described in "Spatio-temporal drivers" sub-section, in Methods (main text).

λ (nm)	LDPE	PP	PS	Bandwidth (nm)
826	Yes	Yes	Yes	10
913	Yes	Yes	Yes	40
972	Yes	Yes	Yes	30
1100	Yes	Yes	Yes	30
1160	Yes	Yes	No	25
1217	No	No	Yes	30
1325	Yes	Yes	Yes	20
1500	Yes	Yes	Yes	10
1531	No	Yes	Yes	15
1573	No	Yes	Yes	10
1589	Yes	No	No	10
1612	Yes	No	No	15
1642	Yes	Yes	No	15
1782	Yes	Yes	Yes	30
2031	Yes	Yes	Yes	40
2080	Yes	Yes	No	40
2155	Yes	Yes	No	50
2251	No	No	Yes	50
2330	Yes	No	No	50

Table S33 | Band centres and widths identified as potentially useful for a fine approach.

λ (nm)	LDPE	PP	PS	Bandwidth (nm)
826	Yes	Yes	Yes	70
913	Yes	Yes	Yes	70
972	Yes	Yes	Yes	60
1100	Yes	Yes	Yes	50
1325	Yes	Yes	Yes	50
1515	Yes	Yes	Yes	65
1615	Yes	Yes	Yes	85
1782	Yes	Yes	Yes	70
2031	Yes	Yes	Yes	100
2251	Yes	No	Yes	210

Table S34 | Band centres and widths identified as potentially useful for a broad approach.

Parameter	Description
Instrument	Super Spectrometer Instrument (SSI)
Field of View	20.6º (290 km projected over surface according to orbit)
Maximum Viewing Zenith Angle	15º
Orbit	Sun-synchronous at altitude 786 km (potentially reducible to improve SNR and reduce revisiting times)<br><br>Mean Local Solar Time at descending node: 10:30 (optimum Sun illumination for image acquisition)<br><br>Inclination of 98.62º<br><br>Period of 100.6 min
Geometric revisiting time	Five days from two-satellite constellation (at Equator)
Spectral range	[0.4 µm -2.4 µm] (VIS + VNIR + SWIR)
# of spectral bands	23 bands
Spatial Resolution	20 m (with current technology)<br><br>10 m (with next generation of sensors)
Minimum plastic content	1% of the pixel

Table S35 | Proposed mission specifications for the EO4ML mission concept.

Application	Parameters	20 m	10 m	5 m
Monitoring of large ML items at the ocean	GSD	No	No	Threshold
	Revisiting time	(Threshold)	(Threshold)	(Threshold)
	Coverage	Goal	Goal	Goal
	AOIs	Goal	Goal	Goal
	Objectives	No	No	Threshold
Monitoring of concentrations of ML at global scale	GSD	Goal	Goal	Goal
	Revisiting time	Goal	Goal	Goal
	Coverage	Goal	Goal	Goal
	AOIs	Goal	Goal	Goal
	Objectives	No	No	No
Monitoring of hot spots and accumulation zones	GSD	Goal	Goal	Goal
	Revisiting time	(Threshold)	(Threshold)	(Threshold)
	Coverage	Goal	Goal	Goal
	AOIs	Threshold (Goal)	Threshold (Goal)	Threshold (Goal)
	Objectives	Threshold (Goal)	Threshold (Goal)	Threshold (Goal)
Monitoring of river mouths as main input flow for ML	GSD	Threshold	Threshold	Threshold (Goal)
	Revisiting time	No (Threshold)	No (Threshold)	No (Threshold)
	Coverage	Goal	Goal	Goal
	AOIs	Goal	Goal	Goal
	Objectives	Threshold	Threshold	Threshold (Goal)
Monitoring of ML at shores and beaches	GSD	No	Threshold	Threshold (Goal)
	Revisiting time	No	No	No
	Coverage	Goal	Goal	Goal
	AOIs	Goal	Goal	Goal
	Objectives	Unlikely	Unlikely	Unlikely

Table S36 | Applications vs. Mission requirements and Mission Specifications. The term "Goal" means that the proposed mission specifications comply with the most restrictive MRs of the targeted application; "Threshold" indicates that the mission specifications comply with the minimum MRs; while "No" means that the proposed mission cannot comply with them. Terms in parenthesis indicate that they could be achieved by revisiting some of the mission parameters or by improving the technology.

Supplementary References

1. Martínez-Vicente, V. et al. Measuring marine plastic debris from space: initial assessment of observation requirements. *Remote Sens.* **11**, 2443 (2019).
2. Maximenko, N. et al. Towards the integrated marine debris observing system. *Front. Mar. Sci.* **6**, 447 (2019).
3. Garaba, S.P. et al. Concentration, anisotropic and apparent colour effects on optical reflectance properties of virgin and ocean-harvested plastics. *J. Hazard. Mater.* **406**, 124290 (2021).
4. deVries, R.V., Garaba, S.P. & Royer, S.J. Hyperspectral reflectance of pristine, ocean weathered and biofouled plastics from dry to wet and submerged state. *Earth Syst. Sci. Data Discuss*, essd-2023-209 (2023).
5. Papageorgiou, D., Topouzelis, K., Suaria, G., Aliani, S. & Corradi, P. Sentinel-2 detection of floating marine litter targets with partial spectral unmixing and spectral comparison with other floating materials (Plastic Litter Project 2021). *Remote Sens.* **14**, 5997 (2022).
6. Topouzelis, K., Papageorgiou, D., Karagaitanakis, A., Papakonstantinou, A., & Arias, M. Remote sensing of sea surface artificial floating plastic targets with Sentinel-2 and unmanned aerial systems (Plastic litter project 2019). *Remote Sens.* **12,** 2013 (2020).
7. Kikaki, K., Kakogeorgiou, I., Mikeli, P., Raitsos, D.E. & Karantzalos, K. MARIDA: a benchmark for marine debris detection from Sentinel-2 remote sensing data. *PLoS One* **17**, e0262247 (2022).
8. Biermann, L., Clewley, D., Martinez-Vicente, V. & Topouzelis, K. Finding plastic patches in coastal waters using optical satellite data. *Sci. Rep.* **10**(1), 5364. (2020)
9. Duarte, M. M. & Azevedo, L. Automatic detection and identification of flaoting marine debris using multispectral satellite imagery. *IEEE Trans. Geosci. and Remote Sens.* **61***,* 1-15 (2023).
10. S. Aliani et al. Marine Litter Windrows. *Front. Mar. Sci. **8**, 827907* (2022).
11. Cózar, A. et al. Marine litter windrows: a strategic target to understand and manage the ocean plastic pollution. *Front. Mar. Sci.* **8**, 571796 (2021).
12. Ruiz, I. et al. (2020). Litter windrows in the south-east coast of the Bay of Biscay: an ocean process enabling effective active fishing for litter. *Front. Mar. Sci.* **7**, 308 (2020).
13. Shanks, A.L. Observational evidence and open questions on the role of internal tidal waves on the concentration and transport of floating plastic debris. *Front. Mar. Sci.* **8**, 621062 (2021).
14. Gallardo, C. et al. Sea-surface slicks and their effect on the concentration of plastics and zooplankton in the coastal waters of Rapa Nui (Easter Island). *Front. Mar. Sci.* **8**, 688224 (2021).
15. Morales-Caselles, C. et al. An inshore-offshore sorting system revealed from global classification of ocean litter. *Nat. Sustain.* **4,** 484-493 (2021).
16. Cózar, A. et al. Plastic debris in the open ocean. *Proc. Natl. Acad. Sci. USA* **111**, 10239-10244 (2014).
17. Lebreton, L. et al. Evidence that the great pacific garbage patch is rapidly accumulating plastic. *Sci. Rep.* **8**, 4666 (2018).
18. Eriksen, M. et al. Plastic pollution in the world's oceans: more than 5 trillion plastic pieces weighing over 250,000 tons afloat at sea. *PLoS One* **9**, 1-15 (2014).
19. Goldstein, M. C., Titmus, A. J. & Ford, M. Scales of spatial heterogeneity of plastic marine debris in the Northeast Pacific Ocean. *PLoS One* **8**, e80020 (2013).
20. Pedrotti, M.L et al. An integrative assessment of the plastic debris load in the Mediterranean Sea. *Sci. Total Environ.* **838**, 155958 (2022).
21. Garaba, S. P. et al. Sensing ocean plastics with an airborne hyperspectral shortwave infrared imager. *Environ. Sci. Technol.* **52**, 11699-11707 (2018)
22. Garaba, S. P. & Dierssen, H. M. An airborne remote sensing case study of synthetic hydrocarbon detection using short wave infrared absorption features identified from marine-harvested macro- and microplastics: *Remote Sens. Environ.* **205**, 224-235 (2018).
23. Mobley, C. D. (1994). Light and water: Radiative transfer in natural waters. Academic press.
24. Kooi M. et al. The effect of particle properties on the depth profile of buoyant plastics in the ocean. *Sci. Rep.* **6**, 33882 (2016).
25. Song, Y. K. et al. Horizontal and vertical distribution of microplastics in Korean coastal waters. *Environ. Sci. Technol.* **52**, 12188-12197 (2018).

26. Cox, C. & Munk, W. Measurement of the roughness of the sea surface from photographs of the sun glitter. *Josa* **44**, 838-850 (1954).
27. Nakajima, T. & Tanaka, M. Effect of wind-generated waves on the transfer of solar radiation in the atmosphere-ocean system. *J. Quant. Spectrosc. Radiat. Transf.* **29**, 521-537 (1983).
28. Jin, Z., Charlock, T. P., Rutledge, K., Stamnes, K. & Wang, Y. Analytical solution of radiative transfer in the coupled atmosphere-ocean system with a rough surface. *Appl. Opt.* **45**, 7443-7455 (2006).
29. Mobley, C. D. Optical modeling of ocean waters: Is the case 1- case 2 classification still useful?. *Oceanogr.* **17**, 60–67 (2004).
30. Lee, Z. & Hu, C. Global distribution of case-1 waters: An analysis from Seawifs measurements. *Remote Sens. Environ.* **101**, 270–276 (2006).
31. Voss, K. J. & Zhang, H. Bidirectional reflectance of dry and submerged labsphere spectralon plaque. *Appl. Opt.* **45**, 7924-7927 (2016).
32. Goddijn-Murphy, L. & Dufaur, J. Proof of concept for a model of light reflectance of plastics floating on natural waters. *Mar. Pollut. Bull.* **135**, 1145-1157 (2018).
33. Berger, K., Reshetouski, I., Magnor, M.A. & Ihrke, I. (2011) Measuring BRDFs of Immersed Materials. In VMV, pp. 325-330.
34. Gasteiger, J. et al. Representative wavelengths absorption parameterization applied to satellite channels and spectral bands. *J. Quant. Spectrosc. Radiat. Transf.* **148**, 99-115 (2014).
35. Hess, M., Koepke, P. & Schult, I. Optical properties of aerosols and clouds: The software package OPAC. *Bull. Am. Meteorol. Soc.* **79**, 831-844 (1998).
36. Hess, M., Koepke, P., Schreier, F. & Trautmann, T. (2014). The aerosol model opac-contents and new software. GEISA Workshop: Towards a new vision of spectroscopic databases, 3 - 4 June 2014, Paris.
37. Emde, C., Buras, R., Mayer, B. & Blumthaler, M. The impact of aerosols on polarized sky radiance: Model development, validation, and applications. *Atmos. Chem. Phys.* **10**, 383-396 (2010).
38. Buras, R. & Mayer, B. (2011). Efficient unbiased variance reduction techniques for monte carlo simulations of radiative transfer in cloudy atmospheres: the solution. *J. Quant. Spectrosc. Radiat. Transf.* **112**, 434-447 (2011).
39. Qi, L., Lee, Z., Hu, C. & Wang, M. Requirement of minimal signal-to-noise ratios of ocean color sensors and uncertainties of ocean color products. *J. Geophys. Res. Oceans* **122**, 2595-2611 (2017).
40. Martí, E. et al. The colors of the ocean plastics. *Environ. Sci. Technol.* **54**, 6594-6601 (2020).
41. Dierssen, H.M., Chlus, A., & Russell, B. Hyperspectral discrimination of floating mats of seagrass wrack and the macroalgae Sargassum in coastal waters of Greater Florida Bay using airborne remote sensing. *Remote Sens. Environ*. **167**, 247-258 (2015).
42. Skakun, S., Vermote, E.F., Roger, J.C., Justice, C.O. & Masek, J.G. Validation of the LaSRC cloud detection algorithm for Landsat 8 images. *IEEE J. Sel. Top. Appl. Earth Obs. Remote Sens.* **12**, 2439-2446 (2019).
43. Mifdal, J., Longépé, N. & Rußwurm, M. Towards detecting floating objects on a global scale with learned spatial features using Sentinel 2. *ISPRS Ann. Photogramm. Remote Sens. Spatial Inf. Sci. (2021)*, pp. 285-293, 10.5194/isprs-annals-V-3-2021-285-2021 (2021).
44. Booth, H., Ma, W., & Karakuş, O. High-precision density mapping of marine debris and floating plastics via satellite imagery. *Sci. Rep.* **13**, 6822 (2023).
45. Politikos, D. V., Adamopoulou, A., Petasis, G., & Galgani, F. (2023). Using artificial intelligence to support marine macrolitter research: A content analysis and an online database. *Ocean Coast. Manag.* **233**, 106466 (2023).
46. Rußwurm, M., Venkatesa, S.J. & Tuia, D. Large-scale Detection of Marine Debris in Coastal Areas with Sentinel-2. *arXiv preprint arXiv:2307.02465* (2023).
47. Hu, C. Remote detection of marine debris using Sentinel-2 imagery: a cautious note on spectral interpretations. *Mar. Pollut. Bull.* **183**, 114082 (2022).
48. Hu, C. Remote detection of marine debris using satellite observations in the visible and near infrared spectral range: Challenges and potentials. *Remote Sens. Environ.* **259**, 112414 (2021).
49. Tarrio, K. et al. Comparison of cloud detection algorithms for Sentinel-2 imagery. *Sci. Remote Sens.* **2**, 100010 (2020).
50. Louis, J. et al. (2016) Sentinel-2 Sen2Cor: L2A Processor for users. 'Living Planet Symposium 2016', 9-13 May 2016, Prague, Czech Republic (ESA SP-740, August 2016).

51. Vanhellemont, Q. & Ruddick, K. Atmospheric correction of meter-scale optical satellite data for inland and coastal water applications. *Remote Sens. Environ.* **216**, 586-597 (2018).

52. Saulquin, B., Fablet, R., Bourg, L., Mercier, G. & d'Andon, O. F. MEETC2: Ocean color atmospheric corrections in coastal complex waters using a Bayesian latent class model and potential for the incoming Sentinel 3-OLCI mission. *Remote Sens. Environ.* **172**, 39-49 (2016).

53. Frantz, D., Haß, E., Uhl, A., Stoffels, J. & Hill, J. Improvement of the Fmask algorithm for Sentinel-2 images: Separating clouds from bright surfaces based on parallax effects. *Remote Sens. Environ.* **215**, 466-481 (2018).

54. Gascon, F. & Ramoino, F. (2017). Sentinel-2 data exploitation with ESA's sentinel-2 toolbox. In EGU General Assembly Conference Abstracts (p. 19548).

55. Farr, T. G. et al. The shuttle radar topography mission. *Rev. Geophys.* **45**, RG2004 (2007).

56. Topouzelis, K., Papakonstantinou, A. & Garaba, S. P. (2019). Detection of floating plastics from satellite and unmanned aerial systems (Plastic Litter Project 2018). *Int. J. Appl. Earth Obs. Geoinf.* **79**, 175-183 (2019).

57. Hu, C., Qi, L., Xie, Y., Zhang, S. & Barnes, B. B. Spectral characteristics of sea snot reflectance observed from satellites: Implications for remote sensing of marine debris. *Remote Sens. Environ.* **269**, 112842 (2022).

58. Themistocleous, K., Papoutsa, C., Michaelides, S., & Hadjimitsis, D. Investigating detection of floating plastic litter from space using sentinel-2 imagery. *Remote Sens.* **12**(16), 2648 (2020).

59. Moshtaghi, M., Knaeps, E., Sterckx, S., Garaba, S., & Meire, D. (2021). Spectral reflectance of marine macroplastics in the VNIR and SWIR measured in a controlled environment. *Sci. Rep.* **11**, 5436 (2021).

60. Filella, M. Questions of size and numbers in environmental research on microplastics: Methodological and conceptual aspects. *Environ. Chem.* **12**, 527-538 (2015).

61. Goddijn-Murphy, L., Peters, S., van Sebille, E., James, N. A. & Gibb, S. Concept for a hyperspectral remote sensing algorithm for floating marine macro plastics: *Mar. Pollut. Bull.* **126**, 255-262 (2018).

62. Efimova, I., Bagaeva, M., Bagaev, A., & Chubarenko, I. P. Secondary microplastics generation in the sea swash zone with coarse bottom sediments: laboratory experiments. *Front. Mar. Sci.* **5**, 313 (2018).

63. Pedrotti, M. L. et al. Changes in the floating plastic pollution of the Mediterranean Sea in relation to the distance to land. *Plos One* **11**, e0161581 (2016).

64. Isobe, A. et al. A multilevel dataset of microplastic abundance in the world's upper ocean and the Laurentian Great Lakes. *Micropl. & Nanopl.* **1**, 16 (2021).

65. Suaria, G. et al. The Mediterranean Plastic Soup: synthetic polymers in Mediterranean surface waters. *Sci. Rep.* **6**, 37551 (2016).

66. Wang, M. & Hu, C. Mapping and Quantifying Sargassum Distribution and coverage in the Central West Atlantic Using MODIS Observations. *Remote Sens. Environ.* **183**, 350-367 (2016).

67. Wang, M. et al. The great Atlantic sargassum belt. *Science* **365**, 83-87 (2019).

68. González-Fernández, D. et al. Floating macrolitter leaked from Europe into the ocean. *Nat. Sustain.* **4**, 474-483 (2021).

69. Acuña-Ruz, T. et al. Anthropogenic marine debris over beaches: Spectral characterization for remote sensing applications. *Remote Sens. Environ.* **217**, 309-322 (2018).

70. Montanari, A. Hydrology of the Po River: looking for changing patterns in river discharge *Hydrol. Earth Syst. Sci.* **16**, 3739–3747 (2012).

71. Campanale, C. et al. Visual observations of floating macro litter around Italy (Mediterranean Sea). *Med. Mar. Sci.* **20**, 271-281 (2019).

72. Arcangeli A., et al. Amount, composition, and spatial distribution of floating macro litter along fixed trans-border transects in the Mediterranean basin. *Mar Pollut Bull.* **129**: 545-554 (2018).

73. Harrigan, S. et al. GloFAS-ERA5 operational global river discharge reanalysis 1979–present. *Earth Syst. Sci. Data* **12**, 2043-2060 (2020).

74. Liubartseva, S., Coppini, G., Lecci, R. & Creti, S. Regional approach to modelling the transport of floating plastic debris in the Adriatic Sea. *Mar. Pollut. Bull.* **103**, 115-127 (2016).

75. Orlić, M., Gačić, M. & Laviolette, P.E. The currents and circulation of the Adriatic Sea. *Oceanol. Acta* **15**, 109-124 (1992).

76. IUCN-MMPATF (2017). Gulf of Ambracia IMMA Factsheet. IUCN Joint SSC/WCPA Marine Mammal Protected Areas Task Force, 2017.

77. Roma Today (2021). Stop alla plastica in mare, il bilancio delle barriere acchiappa rifiuti: "In meno di 18 mesi recuperate 6 tonnellate". https://www.romatoday.it/green/stop-plastica-mare-risultati-barriere-tevere-aniene.html. Accessed 4 December 2021.

78. Roma Today (2021) Marconi: sulle sponde del Tevere una baraccopoli più grande di Tiberis. https://www.romatoday.it/zone/arvalia/marconi/marconi-tevere-rifiuti-baraccopoli-lungotevere-pietra-papa-.html. Accessed 4 December 2021.

79. Tramoy, R., Blin, E., Poitou, I., Noûs, C., Tassin, B. and Gasperi, J., 2022. Riverine litter in a small urban river in Marseille, France: Plastic load and management challenges. *Waste Manag.* **140**, 154-163 (2022).

80. van Sebille, E. et al. The physical oceanography of the transport of floating marine debris. *Environ. Res. Lett.* **15**, e023003 (2020).

81. Ourmieres, Y., Mansui, J., Molcard, A., Galgani, F. & Poitou, I. The boundary current role on the transport and stranding of floating marine litter: the French Riviera case. *Cont. Shelf Res.* **155,** 11-20 (2018).

82. Macías, D., Cózar, A., Garcia-Gorriz, E., González-Fernández, D. & Stips, A. Surface water circulation develops seasonally changing patterns of floating litter accumulation in the Mediterranean Sea: a modelling approach. *Mar. Pollut. Bull.* **149**, e110619 (2019).

83. Jambeck, J.R. et al. Plastic waste inputs from land into the ocean. *Science* **347**, 768-771 (2015).

84. Lebreton, L., & Andrady, A. Future scenarios of global plastic waste generation and disposal. *Palgrave Commun.* **5**, 6 (2019).

85. Weiss, L., et al. The missing ocean plastic sink: gone with the rivers. *Science* **373**, 107-111 (2021).

86. Rose, A., McKee, J., Urban, M., & Bright, E. (2018). LandScan Global 2017 Data set. Oak Ridge National Laboratory. https://doi.org/10.48690/1524212. Accessed 5 November 2021.

87. Hoornweg, D. & Bhada-Tata, P. (2012) What a waste: a global review of solid waste management. World Bank. http://hdl.handle.net/10986/17388. Accessed 28 Feb 2016.

88. IMF (2016) World Economic Outlook Database. International Monetary Fund. https://www.imf.org/external/pubs/ft/weo/2016/02/weodata/index.aspx. Accessed 28 Nov 2016.

89. Waste Atlas (2016) D-waste. Waste Atlas. http://www.atlas.d-waste.com/. Accessed 4 Aug 2016.

Acronyms

AC	Atmospheric Correction
ADT	Absolute Dynamic Topography
AER	Atmospheric & Environmental Research
AOP	Apparent Optical Property
AOT	Aerosol Optical Thickness
AOI	Area of Interest
APD	Absolute Percentage Difference
ASD	Analytical Spectral Device
ATLAS	Advanced Topographic Laser Altimeter System
BAT	Best Available Technology
BOA	Bottom of the Atmosphere
BRDF	Bi-Directional Reflectance Density Function
CALIOP	Cloud-Aerosol Lidar with Orthogonal Polarization
CDI	Cloud Displacement Index
CDOM	Colored Dissolved Organic Matter
CEOS	Committee on Earth Observation Satellites
Chl-a	Chlorophyll-*a*
CHIME	Copernicus Hyperspectral Imaging Mission
CZMIL	Coastal Zone Mapping and Imaging Lidar
DESIS	DLR Earth Sensing Imaging Spectrometer
DSI	Drought Severity Index
EO	Earth Observation
EO4ML	Earth Observation for Marine Litter
ESA	European Space Agency
EU	European Union
FOV	Field of View
FT-IR	Fourier Transform Infrared Spectroscopy
GEO	Geostationary Orbit
GHG	Green House effect Gas
GPGP	Great Pacific Garbage Patch
GSD	Ground Sampling Distance
HAB	Harmful Algal Blooms
HAPS	High Altitude Pseudo Satellites
HDPE	High-density Polyethylene
HPC	High-Performance Computer
HRSL	High Spectral Resolution LiDAR
IMDOS	Integrated Marine Debris Observing System
IOP	Inherent Optical Property
IVOS	Infrared and Visible Optical Sensors
LDPE	Low-density polyethylene
LiDAR	Laser Imaging Detection and Ranging
LW	Litter Windrow
LWD	Litter Windrow Density
LWD_{mga}	Maximal gridded average LWD
MC	Mission Conceptualization
MERIS	Medium Resolution Imaging Spectrometer
MOC	Minimum Observable Coverage
MODIS	Moderate-Resolution Imaging Spectroradiometer
MPW	Mismanaged plastic waste on land (in tonnes)
MPWP	Production of mismanaged plastic waste (in tonnes per year)
MR	Mission Requirement
MSI	Multi Spectral Imager
MYSTIC	Montecarlo code for the physically correct tracing of photons in cloudy atmospheres
NASA	National Aeronautics and Space Administration
Nd:YAG	Neodymium-doped Yttrium Aluminium Garnet
NDSI	Normalized Differential Snow Index
NDVI	Normalized Difference Vegetation Index
NDWI	Normalized Difference Water Index

NI	Normalized Spectral Index Confusion Matrix
NIR	Near Infrared
NOAA	National Oceanography and Atmospheric Administration
OC	Ocean Colour
OLCI	Ocean and Land Colour Instrument
OLI	Operational Land Imager
OPAC	Optical Properties for Aerosols and Clouds
PAN	Panchromatic
PE	Polyethylene
PET	Polyethylene Terephthalate
PI	Plastic input from land into the ocean (in tonnes per year)
PMLI	Plastic Marine Debris Index
PLP	Plastic Litter Project
PoC	Proof of Concept
PP	Polypropylene
ppm	Parts Per Million
PRISMA	ASI PRecursore IperSpettrale della Missione Applicativa
PS	Polystyrene
PVC	PolyVinyl Chloride
RAA	Relative Azimuthal Angle
REPTRAN	Representative wavelengths absorption parameterization software
RGB	Red-Green-Blue
ROC	Receiver Operating Characteristics
ROI	Region of Interest
RTM	Radiative Transfer Modeling
S2-MSI	Sentinel-2 Multi Spectral Imager
SAR	Synthetic Aperture Radar
SASI	Shortwave infrared Airborne Spectrographic Imager
SCL	Scene Classification
SeaWiFS	Sea-viewing Wide Field-of-view Sensor
SI	Supplementary Information
SNR	Signal-to-Noise Ratio
SNRr	Relative Signal to Noise
SRTM	Shuttle Radar Topography Mission
SSA	Sea Surface Anomaly
SWH	Significant Wave Height
SWIR	Short-wave Infrared
SZA	Sun Zenithal Angle
TCI	True Colour Image
TOA	Top of the Atmosphere
TRL	Technology Readiness Level
UV	Ultra Violet light
VHR	Very High Resolution
VI	Vegetation Index
VIS	Visible
VZA	Viewing Zenithal Angle
WS	Wind Speed
